# Reversible Photoswitching of Isolated Ionic Hemiindigos with Visible Light

**DOI:** 10.1002/cphc.201900963

**Published:** 2020-01-21

**Authors:** Eduardo Carrascosa, Christian Petermayer, Michael S. Scholz, James N. Bull, Henry Dube, Evan J. Bieske

**Affiliations:** ^1^ School of Chemistry The University of Melbourne 3010 Parkville (VIC) Australia; ^2^ Department für Chemie and Munich Center for Integrated Protein Science CIPSM Ludwig-Maximilians-Universität München 81377 Munich Germany; ^3^ School of Chemistry, Norwich Research Park University of East Anglia Norwich NR4 7TJ United Kingdom

**Keywords:** ion mobility, mass spectrometry, molecular switch, photochemistry, photochromism

## Abstract

Indigoid chromophores have emerged as versatile molecular photoswitches, offering efficient reversible photoisomerization upon exposure to visible light. Here we report synthesis of a new class of permanently charged hemiindigos (HIs) and characterization of photochemical properties in gas phase and solution. Gas‐phase studies, which involve exposing mobility‐selected ions in a tandem ion mobility mass spectrometer to tunable wavelength laser radiation, demonstrate that the isolated HI ions are photochromic and can be reversibly photoswitched between *Z* and *E* isomers. The *Z* and *E* isomers have distinct photoisomerization response spectra with maxima separated by 40–80 nm, consistent with theoretical predictions for their absorption spectra. Solvation of the HI molecules in acetonitrile displaces the absorption bands to lower energy. Together, gas‐phase action spectroscopy and solution NMR and UV/Vis absorption spectroscopy represent a powerful approach for studying the intrinsic photochemical properties of HI molecular switches.

## Graphical abstract

**Charge‐tagged hemiindigo photoswitches** are demonstrated to undergo reversible *Z*/*E* photoisomerization in the gas phase, proving that photoinduced structural rearrangements do not rely on a solvent but rather reflect the intrinsic characteristics of the photoswitches.

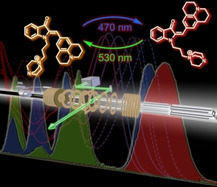

Molecular photoswitches constitute the active core of many light‐responsive molecular machines and motors,^1^, ^2^, ^3^ smart materials, supramolecular systems, and photocontrollable drugs. Many potential applications of photoswitches, particularly in biological contexts require them to respond within the biooptical window, prompting synthetic efforts aimed at shifting their absorptions to longer wavelength.^4^, ^5^, ^6^, ^7^, ^8^, ^9^ Recently, new classes of photoswitches such as DASAs,^10^, ^11^, ^12^ hydrazones,^13^, ^14^ and indigoid chromophores^15^, ^16^, ^17^, ^18^, ^19^ have been developed with strong absorptions in the red region of the spectrum, and with high photoisomerization quantum yields.

Indigoid photoswitches such as hemithioindigo (HTI)^20^, ^21^, ^22^, ^23^ and hemiindigo (HI)^24^, ^25^, ^26^ are chromophores derived from the parent indigo dye.^27^ Although HI molecules were first synthesized over a century ago^24^ and their basic photoswitching characteristics have been known for at least two decades,^25^ interest has recently grown due to the development of new derivatives that respond to red light, have high thermal stability of their switching states, are simple to synthesize and functionalize, and exhibit high photostability.^17^ Recent contributions have reported asymmetric HI systems that exhibit photoswitching of chiroptical properties,^26^ and HTI chromophores that can be deployed as controllable molecular motors,^23^, ^28^, ^29^, ^30^, ^31^, ^32^ receptors^33^, ^34^ or tweezers.^19^, ^35^ Other applications include light controlled peptide structure^36^, ^37^, ^38^ and supramolecular chemistry.^39^ These studies illustrate the broad versatility of indigoid photoswitches and their potential for use in nanotechnological and biomedical applications.^40^, ^41^, ^42^

Developing effective molecular photoswitches requires a detailed understanding of their fundamental photochemical properties and underlying photoisomerization mechanisms, best provided by solid connections between experiment and theory. Although, in principle, comparisons between theory and experiment are best achieved for molecules that are free from the complicating influence of solvent or substrate, there are challenges associated with isolating and characterising the photochemical properties of molecular photoswitches in the gas phase. Therefore, theoretical predictions are often compared with measurements for molecules in solution or the solid state, which are situations where the surrounding medium plays a crucial role.

Recently, we have developed an experimental approach combining ion mobility mass spectrometry and laser spectroscopy to probe the photoisomerization response of photoswitches and biochromophores in the gas phase.^11^, ^43^, ^44^, ^45^, ^46^ In the present work we use this approach to separate and photochemically characterize the *Z* and *E* isomers of three newly developed, charge‐tagged HI photoswitches **1–3** (Figure 1(a)) in the gas phase, with the goal of providing information on their intrinsic photochemical characteristics that can be compared with predictions from quantum chemical calculations and the results from measurements in solution.

**Figure 1:**
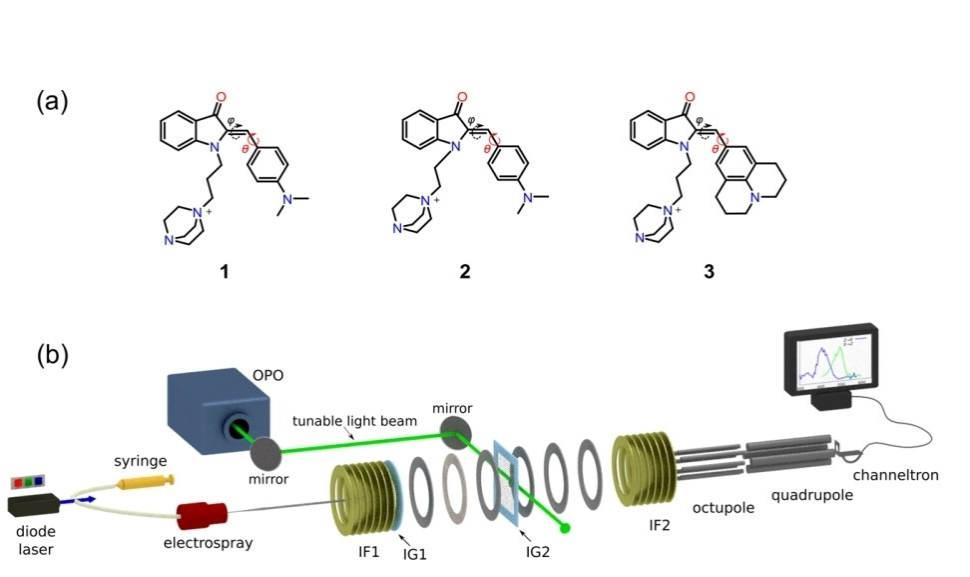
**(a)** *Z*‐isomers of the three investigated HI photoswitches bearing electronically decoupled permanent charge tags. The black dashed arrow indicates rotation of the double bond (*φ*) leading to isomerization, whereas the red arrow shows rotation around the single bond (*θ*) connecting the double bond group to the *N*,*N‐*dimethylaniline/julolidine fragment. **(b)** Schematic representation of the homebuilt tandem ion mobility mass spectrometer (IMS) in which molecular ions are separated according to their collision cross sections with N_2_ buffer gas and exposed to tunable radiation. Electrosprayed ions are collected by an ion funnel (IF1) before being launched through a pulsed ion gate (IG1) into a drift region where they are propelled through N_2_ buffer gas by a 44 V/cm electric field. The drifting ions can be selected by a second ion gate (IG2) and exposed to tunable light from an OPO. Resulting isomer ions are separated in the second stage of the drift region before being collected by a second ion funnel (IF2), directed into an octupole ion guide, quadrupole mass filter, and are finally detected by a Channeltron. Further details can be found in the SI.

We seek to address several key questions. Does reversible *Z/E* photoisomerization occur for isolated HI molecules in the gas phase? Do the *Z* and *E* isomers exhibit distinct photo‐responses? What effect does solvation have on the photo‐response of the HI photoswitches? To some extent, these questions have been explored in a previous study of a related hemithioindigo molecular photoswitch, in which the target species were trapped, tagged with He atoms, and spectroscopically probed in a cryogenic ion trap.^34^ There it was found that *Z*→*E* photoisomerization occurred in the gas phase, but that the reverse process was suppressed, contrasting with the situation in solution where reversible photoisomerization was observed. The hemithioindigo characterized in the earlier study was charge tagged with a trimethyl ammonium group attached at the *para* position of the phenyl ring where it should serve as a strong electron withdrawing group. In contrast, the hemiindigo species investigated in this study have a charge tag attached to the terminus of an alkyl chain, where it should have less influence on the electronic and photochemical properties of the core hemiindigo moiety.

The synthesis and characterization of the HIs **1–3** shown in Figure 1(a) are described in the Supporting Information (SI). In each case, the charge‐tag is peripherally attached to the chromophore via an alkyl chain, minimizing electronic communication to the active section of the molecular photoswitch (Figure 1(a)). Whereas HI **1** and **2** differ in the length of the alkyl chain carrying the charge‐tag, HI **3** incorporates a strong electron donating julolidine moiety. Recent work on the neutral analogues of HIs **1–3** revealed their high thermal bistability, almost perfect reversible photoswitching behaviour in the red region of the spectrum, and strong photo‐ and solvato‐chromism in organic solvents.^17^ The physical and photochemical properties of the neutral HIs were found to depend moderately on the nature of the surrounding solvent.^17^

A primary goal of the current study was to investigate the properties of the target HIs **1–3** in the gas phase. This was accomplished using a tandem ion mobility mass spectrometer (IMS), in which the target *E* or *Z* isomer populations could be separated, and, if desired, selected and exposed to tunable radiation with separation of the product photoisomer. The experimental arrangement is depicted in Figure 1(b) with further details of the tandem IMS and the procedure used to study the photoisomerization of molecular ions is available in the SI and previous publications.^43^, ^46^ Each hemiindigo sample was dissolved in acetonitrile at a concentration of ≈0.1 mm and electrosprayed into the source region of the IMS. Ions were pulse‐injected through an electrostatic ion gate into the first drift region of the IMS, which was filled with N_2_+1 % 2‐propanol buffer gas to a total pressure of ≈6 Torr. The *Z* and *E* isomer ions were separated as they drifted through the buffer gas under the influence of an electric field due to their different collision cross sections and drift velocities, giving rise to individual peaks in the arrival time distribution (ATD). As explained in the SI, addition of the 2‐propanol dopant to the buffer gas resulted in better isomeric separation.

First, we investigated the isomeric composition of the HI solutions by obtaining ATDs whereby the ions were allowed to pass unencumbered through the second ion gate. As shown in Figure 2(a1–a3) it is possible to separate and discriminate the *E* and *Z* isomers for HIs **1–3**. The isomeric composition of the electrosprayed solution could be altered by exposing it to visible light. Exposing the HI solution to blue light prior to electrospray ionization enriched the *E* isomer population, whereas exposure to green or red light enhanced the *Z* isomer population. These changes are clearly apparent in the ATDs shown in Figure 2(a1–a3). Each ATD peak was assigned by considering the change of isomeric abundances following exposure to light of different wavelengths and through consideration of UV/Vis and NMR measurements in solution (See SI).

**Figure 2:**
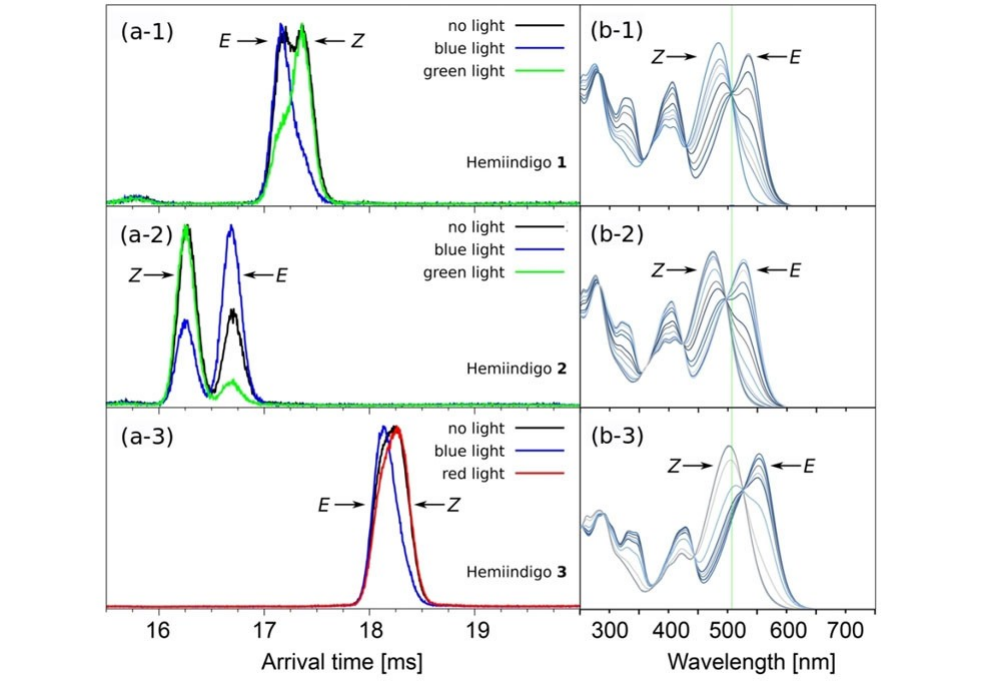
**(a)** Arrival time distributions (ATDs) obtained using N_2_ buffer gas with 1 % 2‐propanol dopant showing the separation of *Z* and *E* isomers of HIs **1–3**. The black traces show ATDs obtained using electrosprayed samples shielded from light, whereas colored traces represent ATDs obtained after exposure of each HI solution to light of the indicated color (blue – 430–480 nm; green – 532 nm; red – 632.8 nm). **(b)** Absorption spectra of an isomeric mixture of HIs **1–3** in acetonitrile solution after irradiation with light of different wavelengths promoting formation of either *Z* or *E* isomers. As a reference, the green line marks the isobestic point for HI 1.

Because rotations around single bonds in the alkyl chain and around the bond adjacent to the isomerizing double bond are energetically feasible at the prevailing effective temperature in the ion mobility spectrometer (T_eff_ ≈300 K), the measured ATDs represent averaged contributions from several conformers. As demonstrated in temperature‐dependent ion mobility measurements, rapid interconversion between different conformations can result in sharp ATD peaks.^47^ To assess the contributions from energetically accessible conformers, a non‐exhaustive molecular mechanics search followed by geometry and energy optimization was carried out using density functional theory (ωB97X‐D/cc‐pVDZ level of theory using the Gaussian16 package^48^). Collision cross sections for the optimized structures were calculated using the MOBCAL package.^49^ Further details of the methodology and results for these calculations are presented in the SI. For each HI molecule, several conformers for each *Z* and *E* isomer were found to have relative energies <10 kcal/mol with respect to the corresponding most stable conformation and may be present under the prevailing experimental conditions (see Figure 3(a–c)). Whereas DFT calculations predict a near planar configuration for *E* isomers of the three HIs, the optimized *Z* isomers show a pronounced twist around the single bond connecting the aniline (julolidine) and indigo planes (torsion angle *θ* in Figure 1(a)). The torsion angles *θ* for the lowest energy conformers of HIs **1**, **2** and **3** are 45°, 52° and 52°, respectively, which are somewhat larger than those calculated at the B3LYP‐GD3BJ/6‐311G(d,p) level of theory with polarizable continuum model for the neutral, solvated Z isomer analogues of **1** (34°) and **3** (37°).^17^

**Figure 3:**
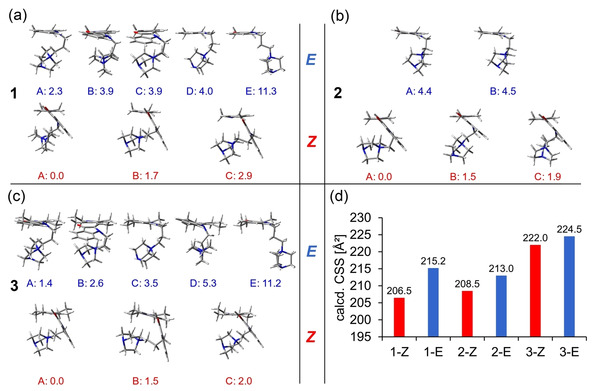
Calculated geometries and relative energies for representative low‐energy *E* and *Z* conformers of HIs **1** (a), **2** (b), and **3** (c). The energies are given in kcal/mol with respect to the most stable conformer in each case. (d) Calculated averaged collision cross section (CCS) for *E* and *Z* isomers in **1**, **2** and **3**, assuming a Boltzmann distribution of the individual conformers shown in (a‐c) and a temperature of 300 K.

The calculated collision cross‐sections in N_2_ buffer gas for the *E* isomers are 1–4 % larger than for the corresponding *Z* isomer for each HI species (see Figure 3(d)), consistent with the observation that for HI **2** the *Z* isomer arrives before the *E* isomer (see Figure 2(a–2)), but at odds with measurements for HI **1** and HI **3**, for both of which the *E* isomer arrives slightly before the *Z* isomer (see Figure 2(a–1), (a–3)). Notwithstanding the discrepancies for the calculated and observed ordering of the arrival times of the *E* and *Z* isomers for HI **1** and HI **3**, which may be influenced by addition of 1 % 2‐propanol dopant added to the N_2_ buffer gas, we are confident of the peak assignments because of the intensity changes provoked by exposure of the solution to light before electrospray ionization (Figures 2(a–1), (a–3)), and because of the wavelength dependence of the photo‐response for the ion populations in the gas phase, as described below.

To study the photoisomerization of HIs **1–3** species in the gas phase, a specific isomer population (either *Z* or *E*) was selected using a pulsed ion gate located halfway along the ion mobility spectrometer drift region (IG2 in Figure 1(b)). Exposure of this isomer population to tunable light from an OPO promoted photoisomerization, with the resulting photoisomer ions separated from the precursor ions in the second drift IMS region. By monitoring the photoisomer ion yield as a function of wavelength in a *laser on‐laser off* arrangement (see Figure 4(a–c)), it was possible to obtain *Z*→*E* and *E*→*Z* photoisomerization action spectra (PISA spectra) as shown in Figures 4(d‐f). All three HI species were found to undergo *Z*→*E* and *E*→*Z* photoisomerization in the gas phase, in contrast to the situation observed in a recent investigation of another charge‐tagged hemithioindigo, where reversible photoisomerization was observed in solution but only *Z*→*E* photoisomerization was observed in the gas phase.^34^ The maxima of the *Z*→*E* PISA spectra of HIs **1, 2** and **3** occur at 450, 410, and 470 nm, respectively, whereas the corresponding *E*→*Z* PISA spectra have maxima at 505, 490, and 520 nm, respectively. The significant red shift observed for the peaks in the PISA spectra of HI **3** compared to HI **1** and **2** can be explained by the presence of the strong electron donating julolidine group, as found recently for the neutral analogue of this compound.^17^ Band maxima for the absorption spectra of HIs in acetonitrile solution are shifted by 30–70 nm to longer wavelength compared to the peaks in the PISA spectra (see Figures 4(g–i)). It is noteworthy that, as shown in Figure 4 (d–f), HIs **1**, **2** and **3** display clear photochromic behavior in the gas phase, with the *Z* isomers responding 40–80 nm to shorter wavelength compared to the *E* isomers, consistent with absorption spectra for the isomers in solution (Figure 4 (g–i)).

**Figure 4:**
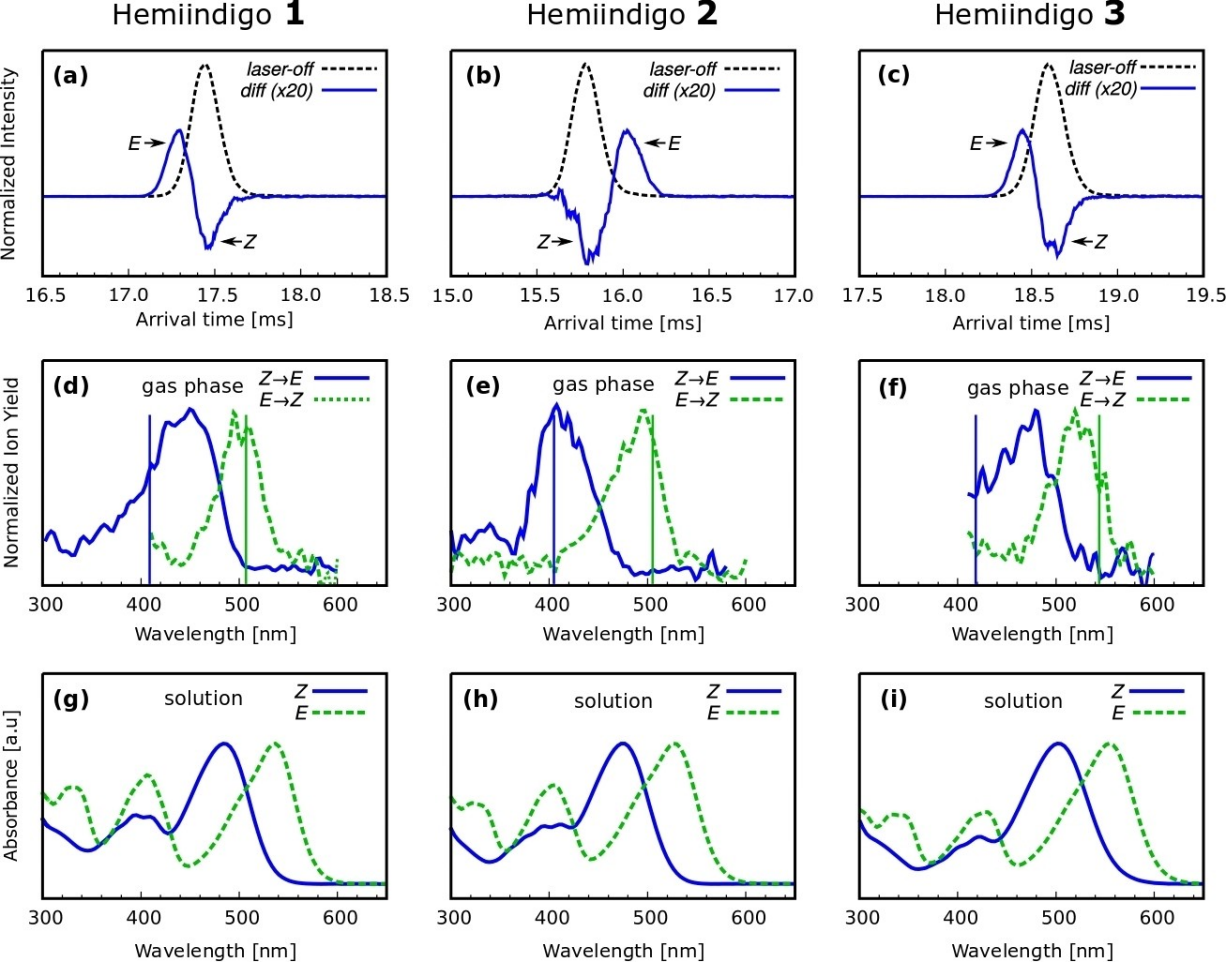
Example gas‐phase *Z* isomer photoisomerization action ATDs **(a–c)**, gas‐phase photoisomerization action spectra (PISA spectra) **(d–f)** and acetonitrile solution absorption spectra **(g–i)** for the *Z* and *E* isomers of HI **1** (left column), **2** (middle column) and **3** (right column). Calculated vertical S_1_←S_0_ excitation wavelengths are marked with vertical lines along with the gas‐phase photoisomerization action spectra **(d–f)**. The photoisomerization action ATDs **(a–c)** were obtained with excitation wavelengths of 450, 420 and 450 nm for HI **1–3**, respectively.

To gain insights into the electronic transitions of HIs **1–3**, we calculated vertical S_1_←S_0_ excitation wavelengths at the df‐CC2/aug‐cc‐pVDZ (aug‐cc‐pVDZ‐RI auxiliary basis set) level of theory using the MRCC software.^50^ There is some variation in the calculated S_1_←S_0_ excitation wavelengths for the various low energy conformers for each HI isomer – generally, conformers with the charge tag adjacent to the donor aniline (HI **1** and **2**) or julolidine (HI **3**) absorb at shorter wavelengths than conformers in which the charge tag is adjacent to the indigo or distant from both the indigo and aniline. This is consistent with expectations based on the donor‐acceptor nature of the electronic transitions whereby a positive charge adjacent to the aniline (julolidine) or indigo stabilizes the S_0_ or S_1_ state, respectively.^19^

Generally, the calculated vertical S_1_←S_0_ transitions for the *Z* and *E* isomers of HIs **1–3** lie in the wavelength range of the measured photoisomerization response (see Figure 4(d–f)). For the *E* isomers, the calculated vertical absorption wavelengths (506, 504, and 543 nm for **1**, **2**, and **3**) correspond closely with the maxima of the PISA spectra (see Figure 4(d–f)). The calculated vertical S_1_←S_0_ excitation wavelength (405 nm) agrees with the peak photo‐response for the *Z* isomer of HI **2**, but the peak photo‐responses for the *Z* isomers of HIs **1** and **3** are shifted to longer wavelength by ≈50 nm compared to the calculated values (409 and 419 nm). This may indicate that for the *Z* isomers of HIs **1** and **3**, which have longer alkyl chains, the conformers probed in the drift tube, have, on average, the charge tag either adjacent the indigo or distant from both the indigo and aniline (julolidine), whereas for the *Z* isomer of HI **2** (shorter alkyl chain), the probed conformers are more likely to have the charge tag adjacent to the aniline. This situation appears to prevail in solution where the ^1^H NMR signals of the charge‐tag protons are shifted significantly upfield for the *Z* isomer of HI **2** compared to HIs **1** and **3**, signifying its closer proximity to the aniline aromatic ring (see SI for details).

In conclusion, we have characterized three permanently charged thermally bistable HIs and reported on their photochemical properties in the gas phase and in solution. For the gas‐phase measurements, the *Z* and *E* isomers were separated by virtue of their different collision cross sections with N_2_ buffer gas allowing measurement of isomer‐specific photoisomerization responses, thereby obviating the need for deconvolution of spectra for isomeric mixtures in solution. All three HIs undergo reversible photoisomerization upon exposure to visible light, exhibiting clear photochromic behaviour in the gas phase and in solution. The gas‐phase photoisomerization action spectra are broadly consistent with calculations which capture their intrinsic molecular properties, particularly the vertical excitation wavelengths. The length of the charge‐bearing alkyl chain affects the photo‐response of the *Z* isomers, conceivably because it dictates the average distance between the charge tag and the donor dimethyl aniline or julolidine. Overall, the tandem IMS approach offers unique insights into the intrinsic photochemical properties of photo‐active molecules free from environment influences. Future investigations of other selected hemiindigo and hemithioindigo isomers in the gas phase should help benchmark theoretical models and build foundations for the rational design of effective molecular photoswitches.

## Conflict of interest

The authors declare no conflict of interest.

## Supporting information

As a service to our authors and readers, this journal provides supporting information supplied by the authors. Such materials are peer reviewed and may be re‐organized for online delivery, but are not copy‐edited or typeset. Technical support issues arising from supporting information (other than missing files) should be addressed to the authors.

Supplementary

## References

[1] W. R. Browne , B. L. Feringa , Annu. Rev. Phys. Chem. 2009, 60, 407–428. 18999995 10.1146/annurev.physchem.040808.090423

[2] J. Andréasson , U. Pischel , Chem. Soc. Rev. 2018, 47, 2266–2279. 29487931 10.1039/c7cs00287d

[3] B. L. Feringa , Angew. Chem. Int. Ed. 2017, 56, 11060–11078; 10.1002/anie.201702979 28851050

[4] M. Dong , A. Babalhavaeji , S. Samanta , A. A. Beharry , G. A. Woolley , Acc. Chem. Res. 2015, 48, 2662–2670. 26415024 10.1021/acs.accounts.5b00270

[5] M. Hammerich , C. Schütt , C. Stähler , P. Lentes , F. Röhricht , R. Höppner , R. Herges , J. Am. Chem. Soc. 2016, 138, 13111–13114. 27685503 10.1021/jacs.6b05846

[6] N. Ankenbruck , T. Courtney , Y. Naro , A. Deiters , Angew. Chem. Int. Ed. 2018, 57, 2768–2798; 10.1002/anie.201700171 PMC6026863 28521066

[7] A. S. Lubbe , Q. Liu , S. J. Smith , J. W. de Vries , J. C. M. Kistemaker , A. H. de Vries , I. Faustino , Z. Meng , W. Szymanski , A. Herrmann , B. L. Feringa , J. Am. Chem. Soc. 2018, 140, 5069–5076. 29551069 10.1021/jacs.7b09476 PMC5909178

[8] D. Bleger , J. Schwarz , A. M. Brouwer , S. Hecht , J. Am. Chem. Soc. 2012, 134, 20597–20600. 23236950 10.1021/ja310323y

[9] Y. Yang , R. P. Hughes , I. Aprahamian , J. Am. Chem. Soc. 2014, 136, 13190–13193. 25222380 10.1021/ja508125n

[10] M. Di Donato , M. M. Lerch , A. Lapini , A. D. Laurent , A. Iagatti , L. Bussotti , S. P. Ihrig , M. Medved′ , D. Jacquemin , W. Szymanski , W. J. Buma , P. Foggi , B. L. Feringa , J. Am. Chem. Soc. 2017, 139, 15596–15599. 29039920 10.1021/jacs.7b09081 PMC5680540

[11] J. N. Bull , E. Carrascosa , N. Mallo , M. S. Scholz , G. da Silva , J. E. Beves , E. J. Bieske , J. Phys. Chem. Lett. 2018, 9, 665–671. 29356541 10.1021/acs.jpclett.7b03402

[12] M. M. Lerch , W. Szymanski , B. L. Feringa , Chem. Soc. Rev. 2018, 47, 1910–1937. 29468232 10.1039/c7cs00772h

[13] Y. Yang , R. P. Hughes , I. Aprahamian , J. Am. Chem. Soc. 2014, 136, 13190–13193. 25222380 10.1021/ja508125n

[14] J. D. Harris , M. J. Moran , I. Aprahamian , Proc. Natl. Acad. Sci. USA 2018, 115, 9414–9422. 30012601 10.1073/pnas.1714499115 PMC6156620

[15] S. Wiedbrauk , H. Dube , Tetrahedron Lett. 2015, 56, 4266–4274.

[16] F. Kink , M. Polo Collado , S. Wiedbrauk , P. Mayer , H. Dube , Chem. Eur. J. 2017, 23, 6237–6243. 28235134 10.1002/chem.201700826

[17] C. Petermayer , S. Thumser , F. Kink , P. Mayer , H. Dube , J. Am. Chem. Soc. 2017, 139, 15060–15067. 28944664 10.1021/jacs.7b07531

[18] C.-Y. Huang , A. Bonasera , L. Hristov , Y. Garmshausen , B. M. Schmidt , D. Jacquemin , S. Hecht , J. Am. Chem. Soc. 2017, 139, 15205–15211. 29019401 10.1021/jacs.7b08726

[19] C. Petermayer , H. Dube , Acc. Chem. Res. 2018, 51, 1153–1163. 29694014 10.1021/acs.accounts.7b00638

[20] P. Friedländer , Chem. Ber. 1906, 39, 1060–1066.

[21] V. A. Izmail′skii , M. A. Mostoslavskii , Ukr. Khem. Zh. 1961, 27, 234–237.

[22] S. Kitzig , M. Thilemann , T. Cordes , K. Ruck-Braun , ChemPhysChem 2016, 17, 1252–1263. 26789782 10.1002/cphc.201501050

[23] M. Guentner , M. Schildhauer , S. Thumser , P. Mayer , D. Stephenson , P. J. Mayer , H. Dube , Nat. Commun. 2015, 6, 8406. 26411883 10.1038/ncomms9406 PMC4598625

[24] A. Baeyer , Ber. Dtsch. Chem. Ges. 1882, 15, 50–56.

[25] T. Arai , M. Ikegami , Chem. Lett. 1999, 28, 965–966.

[26] C. Petermayer , H. Dube , J. Am. Chem. Soc. 2018, 140, 13558–13561. 30303001 10.1021/jacs.8b07839

[27] A. Baeyer , Ber. Dtsch. Chem. Ges. 1883, 16, 2188–2204.

[28] L. A. Huber , K. Hoffmann , S. Thumser , N. Böcher , P. Mayer , H. Dube , Angew. Chem. Int. Ed. 2017, 56, 14536–14539; 10.1002/anie.201708178 28892243

[29] R. Wilcken , M. Schildhauer , F. Rott , L. A. Huber , M. Guentner , S. Thumser , K. Hoffmann , S. Oesterling , R. de Vivie-Riedle , E. Riedle , H. Dube , J. Am. Chem. Soc. 2018, 140, 5311–5318. 29578704 10.1021/jacs.8b02349

[30] E. Uhl , S. Thumser , P. Mayer , H. Dube , Angew. Chem. 2018, 130, 11231–11235; 10.1002/anie.201804716 29932486

[31] E. Uhl , S. Thumser , P. Mayer , H. Dube , Angew. Chem. Int. Ed. 2018, 57, 11064–11068; 10.1002/anie.201804716 29932486

[32] A. Gerwien , P. Mayer , H. Dube , J. Am. Chem. Soc. 2018, 140, 16442–16445. 30449088 10.1021/jacs.8b10660

[33] M. Guentner , E. Uhl , P. Mayer , H. Dube , Chem. Eur. J. 2016, 22, 16433–16436. 27644003 10.1002/chem.201604237

[34] R. Navratil , S. Wiedbrauk , J. Jasik , H. Dube , J. Roithova , Phys. Chem. Chem. Phys. 2018, 20, 6868–6876. 29485646 10.1039/c8cp00096d

[35] S. Wiedbrauk , T. Bartelmann , S. Thumser , P. Mayer , H. Dube , Nat. Commun. 2018, 9, 1456. 29654233 10.1038/s41467-018-03912-7 PMC5899155

[36] T. Cordes , D. Weinrich , S. Kempa , K. Riesselmann , S. Herre , C. Hoppmann , K. Rück-Braun , W. Zinth , Chem. Phys. Lett. 2006, 428, 167–173.

[37] T. Cordes , C. Elsner , T. T. Herzog , C. Hoppmann , T. Schadendorf , W. Summerer , K. Rück-Braun , W. Zinth , Chem. Phys. 2009, 358, 103–110.

[38] S. Kitzig , M. Thilemann , T. Cordes , K. Ruck-Braun , ChemPhysChem 2016, 17, 1252–1263. 26789782 10.1002/cphc.201501050

[39] K. Tanaka , K. Kohayakawa , S. Iwata , T. Irie , J. Org. Chem. 2008, 73, 3768–3774. 18429634 10.1021/jo800091d

[40] T. Lougheed , V. Borisenko , T. Hennig , K. Rück-Braun , G. A. Woolley , Org. Biomol. Chem. 2004, 2, 2798–2801. 15455152 10.1039/B408485C

[41] S. Herre , T. Schadendorf , I. Ivanov , C. Herrberger , W. Steinle , K. Rück-Braun , R. Preissner , H. Kuhn , ChemBioChem 2006, 7, 1089–1095. 16755628 10.1002/cbic.200600082

[42] A. Sailer , F. Ermer , Y. Kraus , F. Lutter , C. Donau , M. Bremerich , J. Ahlfeld , O. Thorn-Seshold , ChemBioChem 2019, 20, 1305–1314. 30633427 10.1002/cbic.201800752

[43] B. D. Adamson , N. J. A. Coughlan , P. B. Markworth , R. E. Continetti , E. J. Bieske , Rev. Sci. Instrum. 2014, 85, 123109. 25554274 10.1063/1.4903753

[44] M. S. Scholz , J. N. Bull , E. Carrascosa , B. D. Adamson , G. K. Kosgei , J. J. Rack , E. J. Bieske , Inorg. Chem. 2018, 57, 5701–5706. 29663799 10.1021/acs.inorgchem.8b00871

[45] E. Carrascosa , J. N. Bull , M. S. Scholz , N. J. A. Coughlan , S. Olsen , U. Wille , E. J. Bieske , J. Phys. Chem. Lett. 2018, 9, 2647–2651. 29724104 10.1021/acs.jpclett.8b01201

[46] P. B. Markworth , B. D. Adamson , N. J. A. Coughlan , L. Goerigk , E. J. Bieske , Phys. Chem. Chem. Phys. 2015, 17, 25676–25688. 26032622 10.1039/c5cp01567g

[47] J. Gidden , M. T. Bowers , J. Phys. Chem. B 2003, 107, 12829–12837.

[48] Gaussian 16, Revision B.01, M. J. Frisch, G. W. Trucks, H. B. Schlegel, G. E. Scuseria, M. A. Robb, J. R. Cheeseman, G. Scalmani, V. Barone, G. A. Petersson, H. Nakatsuji, X. Li, M. Caricato, A. V. Marenich, J. Bloino, B. G. Janesko, R. Gomperts, B. Mennucci, H. P. Hratchian, J. V. Ortiz, A. F. Izmaylov, J. L. Sonnenberg, Williams, F. Ding, F. Lipparini, F. Egidi, J. Goings, B. Peng, A. Petrone, T. Henderson, D. Ranasinghe, V. G. Zakrzewski, J. Gao, N. Rega, G. Zheng, W. Liang, M. Hada, M. Ehara, K. Toyota, R. Fukuda, J. Hasegawa, M. Ishida, T. Nakajima, Y. Honda, O. Kitao, H. Nakai, T. Vreven, K. Throssell, J. A. Montgomery Jr., J. E. Peralta, F. Ogliaro, M. J. Bearpark, J. J. Heyd, E. N. Brothers, K. N. Kudin, V. N. Staroverov, T. A. Keith, R. Kobayashi, J. Normand, K. Raghavachari, A. P. Rendell, J. C. Burant, S. S. Iyengar, J. Tomasi, M. Cossi, J. M. Millam, M. Klene, C. Adamo, R. Cammi, J. W. Ochterski, R. L. Martin, K. Morokuma, O. Farkas, J. B. Foresman, D. J. Fox, Wallingford, CT, 2016.

[49] A. A. Shvartsburg , M. F. Jarrold , Chem. Phys. Lett. 1996, 261, 86–91.

[50] MRCC, a Quantum Chemical Program Suite. Kállay, Z. Rolik, J. Csontos, P. Nagy, G. Samu, D. Mester, J. Csóka, B. Szabó, I. Ladjánszki, L. Szegedy, B. Ladóczki, K. Petrov, M. Farkas, P. D. Mezei, B. Hégely, www.mrcc.hu. 10.1063/1.5142048 32087669

