# Supplementary material for: Reversible Photoswitching of Isolated Ionic Hemiindigos with Visible Light
Source: Chemphyschem. 2020 Jan 21;21(7):680–5. doi: 10.1002/cphc.201900963 (PMC7277040; doi:10.1002/cphc.201900963)
Supplement: Supplementary file 1 — Supplementary [file CPHC-21-680-s001.pdf]

CHEMPHYSCHEM

Supporting Information

Reversible Photoswitching of Isolated Ionic Hemiindigos with Visible Light

Eduardo Carrascosa[+], Christian Petermayer[+], Michael S. Scholz, James N. Bull, Henry Dube,* and Evan J. Bieske*© 2019 The Authors. Published by Wiley-VCH Verlag GmbH & Co. KGaA. This is an open access article under the terms of the Creative Commons Attribution Non-Commercial License, which permits use, distribution and reproduction in any medium, provided the original work is properly cited and is not used for commercial purposes.

Table of Contents

Synthesis 2

General experimental 2

Synthesis of hemiindigos 6 - 8 3

(Z/E)-1-(3-bromopropyl)-2-(4-(dimethylamino)benzylidene)indolin-3-one (6) 4

(*Z*/*E*)-1-(2-bromoethyl)-2-(4-(dimethylamino)benzylidene)indolin-3-one (7) 5

(*Z*/*E*)-1-(3-bromopropyl)-2-((2,3,6,7-tetrahydro-1*H*,5*H*-pyrido[3,2,1-ij]quinolin-9-yl)methylene)indolin-3-one (8) 6

Synthesis of charge-tagged hemiindigos 1 - 3 8

(*Z*/*E*)-1-(3-(2-(4-(dimethylamino)benzylidene)-3-oxoindolin-1-yl)propyl)-1,4-diazabicyclo[2.2.2]octan-1-ium bromide (1) 9

(*Z*/*E*)-1-(2-(2-(4-(dimethylamino)benzylidene)-3-oxoindolin-1-yl)ethyl)-1,4-diazabicyclo[2.2.2]octan-1-ium hexafluorophosphate (2) 10

(*Z*/*E*)-1-(3-(3-oxo-2-((2,3,6,7-tetrahydro-1*H*,5*H*-pyrido[3,2,1-ij]quinolin-9-yl)methylene)indolin-1-yl)propyl)-1,4-diazabicyclo[2.2.2]octan-1-ium bromide(3) 11

Theoretical methods and calculated conformer structures and energies 13

Gas phase photoisomerization experiments 16

ATD peak assignments and determination of isomer yields upon irradiation in solution 18

Photoisomerization action spectroscopy experiments 19

Power dependence of the photoisomerization yield 19

Solution photoisomerization experiments 20

Determination of the UV-vis absorption spectra of *Z* and *E* isomers 20

PSS UV-Vis spectra 25

Comparison of chain length effects via NMR 27

Conformational analysis in solution 28

NMR Spectra 32

References 39

Appendix: Cartesian coordinates for calculated structures 40

Synthesis

General experimental

Reagents and solvents were obtained from *Acros*, *Aldrich*, *Fluka*, *Merck*, or *Sigma-Aldrich* in the qualities *puriss*., *p.a*., or *purum* and used as received. Technical solvents were distilled before use for column chromatography and extraction on a rotary evaporator (*vacuubrand* CVC 3000). Reactions were monitored on *Merck* Silica 60 F_{254} or Aluminium oxide 60 F_{254} neutral TLC plates and irradiation with UV light (254 nm or 366 nm).

Column chromatography was performed with silica gel 60 (*Merck*, particle size 0.063 - 0.200 mm) or aluminium oxide (*Sigma-Aldrich*, pore size 58 Å, Brockmann III, 6 % water w/w) and distilled technical solvents.

^{1}H NMR and ^{13}C NMR spectra were measured on a *Varian Mercury 200 VX*, *Varian 300, Inova 400*, *Varian 600 NMR*, or *Bruker Avance III HD 800 MHz* spectrometer. Chemical shifts (δ) are given relative to tetramethylsilane as external standard. Deuterated solvents were obtained from *Cambridge Isotope Laboratories* and used without further purification. Residual solvent signals in the ^{1}H and ^{13}C NMR spectra were used as internal reference: For ^{1}H NMR: CD_2Cl_2 = 5.32 ppm, CD_3CN = 1.94 ppm. For ^{13}C NMR: CD_2Cl_2 = 53.84 ppm, CD_3CN = 118.26, 1.32 ppm. The resonance multiplicity is indicated as *s* (singlet), *d* (doublet), *t* (triplet), *q* (quartet), *quin* (quintet), *sext* (sextet), *sept* (septett), *m* (multiplet) and *br* (broad signal). The chemical shifts are given in parts per million (ppm) on the delta scale (δ) and the coupling constant values (J) are given in hertz (Hz). Signal assignments are given in the experimental part using the arbitrary numbering indicated.

Electron Impact (EI) mass spectra were measured on a *Finnigan MAT95Q* or on a *Finnigan MAT90* mass spectrometer.

Electrospray ionization (ESI) mass spectra were measured on a *Thermo Finnigan LTQ-FT*. The most important signals are reported in *m*/*z* units with *M* as the molecular ion.

UV-vis spectra were measured on a *Varian Cary 5000* spectrophotometer. The spectra were recorded in quartz cuvettes (10 mm path length). Solvents for spectroscopy were obtained from *VWR* and *Merck*. Absorption wavelengths (λ) are reported in nm.

High performance liquid chromatography (HPLC) was performed on a *Merck-Hitachi LaChrom* Series HPLC system consisting of a D-7000 interface, a L-7150 solvent delivery module, a L-7350 column oven, a L-7420 UV-vis detector and a L-7455 diode array detector using a preparative *Machery-Nagel* VP 250/21 *NUCLEODUR Sphinx* RP 5 µm column (acetonitrile/water 7:3-1:0 v/v, 12-15 mL/min, 35 °C column temperature) from *Sigma-Aldrich* and *ROTH*.

Synthesis of hemiindigos 6 - 8

Hemiindigos **4** and **5** were prepared according to literature procedures [1]. Sodium hydride, 1,2-dibromoethane and 1,3-dibromopropane were purchased from *Sigma-Aldrich* and used as received. Hemiindigos **6** - **8** were synthesized according to *V. Velezheva* et *al.* with changes to addition order and addition speed [2].

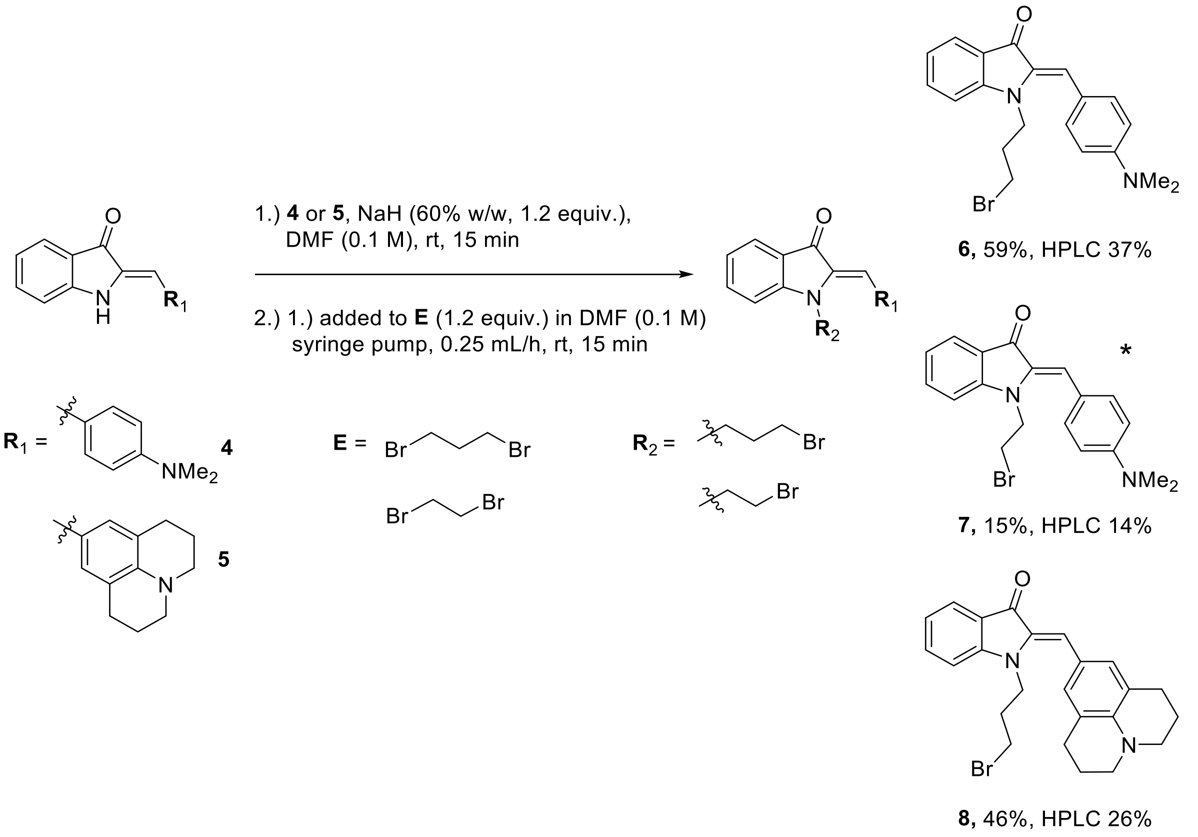

Scheme S1: Preparation of terminally brominated photoswitches. Hemiindigos **6** - **7** were prepared by deprotonation with sodium hydride and a subsequent S_N2 reaction with terminal dibromoalkanes via syringe pump at room temperature. Excess of base tends to eliminate bromine for entries **6** and **8** via E1cB mechanism leaving hemiindigo switches with 1-propene attached to the indoxyl nitrogen as side product (approx. 10 - 20 %).

* 1.5 equivalents sodium hydride and 5.0 equivalents of 1,2-dibromoethane were used. Fast addition of 1,2-dibromoethane to basified mixture of **4** in DMF without syringe pump.

(Z/E)-1-(3-bromopropyl)-2-(4-(dimethylamino)benzylidene)indolin-3-one (6)

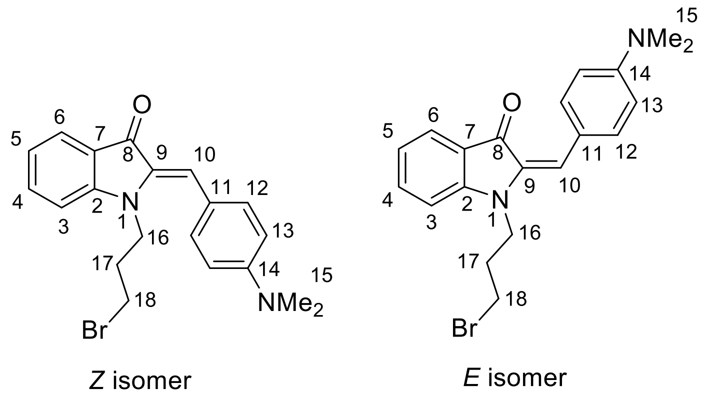

To a sealable, N_2 purged round bottom flask, equipped with a magnetic stirring bar, (*Z*)-2-(4-(dimethylamino)benzylidene)indolin-3-one **4** (0.100 g, 0.378 mmol, 1.0 equiv.) sodium hydride (60% w / w, 0.018 g, 0.454 mmol, 1.2 equiv.) and dimethylformamide (4 mL, 0.1 M) were added and the deep green solution was stirred for 15 min at 23 °C. The mixture was taken up in a syringe and added via syringe pump (0.25 mL/h) to another N_2 purged round bottom flask, equipped with a magnetic stirring bar, filled with 1,3-dibromopropane (0.092 mg, 0.454 mmol, 1.2 equiv.) and dimethylformamide (4 mL, 0.1 M) at 23 °C. After completion of the addition, the mixture was neutralized with aq. sat. ammonium chloride solution, extracted with ethyl acetate, washed ten times with water and treated with brine once. The combined organic phases were dried over sodium sulfate and the volatiles were removed *in vacuo*. Subsequent purification by flash column chromatography (aluminium(III) oxide, *Brockmann*(III), hexanes / ethyl acetate, 4 / 1, v / v) yielded (*Z*/*E*)-1-(3-bromopropyl)-2-(4-(dimethylamino)-benzylidene)indolin-3-one **6** (0.086 g, 0.22 mmol, 59%) as deep red solid. Further purification was carried out via preparative HPLC (*Machery-Nagel* VP 250/21 *NUCLEODUR Sphinx* RP 5 µm column, acetonitrile / water, 8 / 2, v / v, 15 mL/min, 35 °C column temperature, retention times: 9.8 min, 53 mg (37%) of an *E* / *Z* mixture was obtained.

***R*$_f$** = 0.37 (silica, hexanes / ethyl acetate, 4 / 1, v / v); ***Z* isomer: ^{1}H-NMR (600 MHz, CD_2Cl_2):** δ (ppm) = 7.68 (dd, ^3J = 7.6 Hz, ^4J = 1.4 Hz, 1H, H-C(6)), 7.52 (ddd, ^3J = 8.4 Hz, ^3J = 7.1 Hz, ^4J = 1.4 Hz, 1H, H-C(4)), 7.37 - 7.34 (m, 2H, H-C(12)), 7.14 (dd, ^3J = 8.2 Hz, ^4J = 0.8 Hz, 1H, H-C(3)), 7.02 (s, 1H, H-C(10)), 6.97 (ddd, ^3J = 7.7 Hz, ^3J = 7.3 Hz, ^4J = 0.8 Hz, 1H, H-C(5)), 6.76 - 6.73 (m, 2H, H-C(13)), 3.96 (t, ^3J = 7.1 Hz, 2H, H-C(16)), 3.04 (t, ^3J = 6.7 Hz, 2H, H-C(18)), 3.03 (s, 6H, H-C(15)), 1.80 (*quin*, ^3J = 6.9 Hz, 2H, H-C(17)); **^{13}C-NMR (101 MHz, CD_2Cl_2):** δ (ppm) = 186.98 (C(8)), 155.87 (C(2)), 150.97 (C(14)), 135.90 (C(4)), 134.29 (C(9)), 132.71 (2C, C(12)), 124.79 (C(6)), 123.76 (C(7)), 121.47 (C(11)), 118.78 (C(5)), 116.54 (C(10)), 112.11 (C(3)), 112.11 (2C, C(13)), 44.07 (C(16)), 40.51 (2C, C(15)), 31.17 (C(18)), 30.17 (C(17)); ***E* isomer: ^{1}H-NMR (600 MHz, CD_2Cl_2):** δ (ppm) = 8.20 - 8.17 (m, 2H, H-C(12)), 7.65 (dd, ^3J = 7.5 Hz, ^4J = 1.3 Hz, 1H, H-C(6)), 7.46 (ddd, ^3J = 8.4 Hz, ^3J = 7.1 Hz, ^4J = 1.4 Hz, 1H, H-C(4)), 7.01 (dd, ^3J = 8.4 Hz, ^4J = 0.7 Hz, 1H, H-C(3)), 6.85 (ddd, ^3J = 7.7 Hz, ^3J = 7.2 Hz,

^3J = 0.8 Hz, 1H, H-C(5)), 6.73 - 6.71 (m, 2H, H-C(13)), 6.48 (s, 1H, H-C(10)), 4.00 (t, ^3J = 6.9 Hz, 2H, H-C(16)), 3.50 (t, ^3J = 6.2 Hz, 2H, H-C(18)), 3.04 (s, 6H, H-C(15)), 2.30 (*quin*, ^3J = 6.7 Hz, 2H, H-C(17)); **^{13}C-NMR (151 MHz, CD_2Cl_2):** δ (ppm) = 183.85 (C(8)), 151.82 (C(14)), 151.57 (C(2)), 135.56 (C(4)), 132.93 (2C, C(12)), 132.71 (C(9)), 124.66 (C(6)), 122.71 (C(11)), 122.06 (C(7)), 120.62 (C(5)), 120.54 (C(10)), 111.67 (2C, C(13)), 109.20 (C(3)), 40.92 (C(16)), 40.46 (2C, C(15)), 31.85 (C(18)), 31.24 (C(17)); **MS (EI+, 70 eV):** *m*/*z* (%) = 386.1 (20), 384.1 (22), 305.2 (24), 304.2 (100), 303.1 (16), 277.1 (17), 264.1 (17), 263.1 (83), 248.1 (30), 247.1 (20), 232.1 (14), 220.1 (19), 219.1 (19), 159.1 (11), 134.1 (14), 57 (12); **HRMS (EI+, $C_{20}H_{21}BrN_2O$):** calcd.: 384.0837; found: 384.0831(M+).

(*Z*/*E*)-1-(2-bromoethyl)-2-(4-(dimethylamino)benzylidene)indolin-3-one (7)

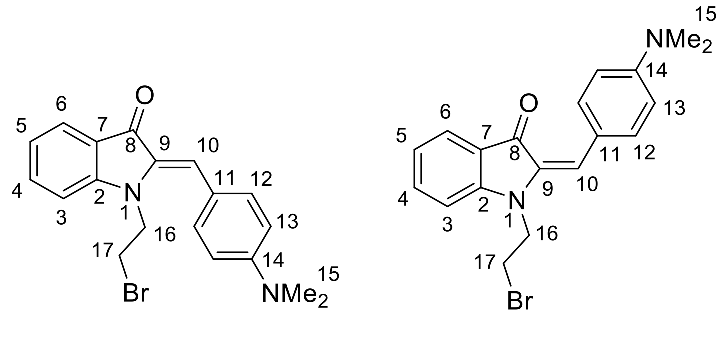

Z isomer *E* isomer

To a sealable, N_2 purged round bottom flask, equipped with a magnetic stirring bar, (*Z*)-2-(4-(dimethylamino)benzylidene)indolin-3-one **4** (0.200 g, 0.757 mmol, 1.0 equiv.) sodium hydride (60% w / w, 0.045 g, 1.135 mmol, 1.5 equiv.) and dimethylformamide (8 mL, 0.1 M) were added and the deep green solution was stirred for 15 min at 23 °C. 1,2-dibromoethane (0.711 g, 3.78 mmol, 5.0 equiv.) was added and the solution mixture was stirred for 30 min at 23 °C. The mixture was neutralized with aq. sat. ammonium chloride solution, extracted with ethyl acetate, washed ten times with water and treated with brine once. The combined organic phases were dried over sodium sulfate and the volatiles were removed *in vacuo*. Subsequent purification by flash column chromatography (aluminium(III) oxide, *Brockmann*(III), hexanes / ethyl acetate, 8 / 2, v / v) yielded (*Z*/*E*)-1-(3-bromoethyl)-2-(4-(dimethylamino)benzylidene)indolin-3-one **7** (0.042 g, 0.113 mmol, 15%) as deep red solid. Further purification was carried out via preparative HPLC (*Machery-Nagel* VP 250/21 *NUCLEODUR Sphinx* RP 5 µm column, acetonitrile / water, 9 / 1, v / v, 15 mL/min, 35 °C column temperature, retention times: 5.4 min, 38 mg (14%) of an *E*/*Z* mixture was obtained.

R$_f$ = 0.34 (silica, hexanes / ethyl acetate, 4 / 1, v / v); ***Z* isomer:** **^{1}H-NMR (400 MHz, CD_2Cl_2):** δ (ppm) = 7.70 (dd, ^3J = 7.7 Hz, ^4J = 1.4 Hz, 1H, H-C(6)), 7.54 (ddd, ^3J = 8.4 Hz, 7.2 Hz, 1.4 Hz, 1H, H-C(4)), 7.39 - 7.33 (m, 2H, H-C(12)), 7.14 (d, ^3J = 8.3 Hz, 1H, H-C(3)), 7.04 (s, 1H, H-C(10)),7.01 (ddd, ^3J =

7.4 Hz, ^3J = 6.3 Hz, ^4J = 0.9 Hz, 1H, H-C(5)), 6.78 - 6.74 (m, 2H, H-C(13)), 4.20 (t, ^3J = 7.4 Hz, 2H, H-C(16)), 3.16 (t, ^3J = 7.3 Hz, 7.1 Hz, 2H, H-C(17)), 3.05 (s, 6H, H-C(15)); **^{13}C-NMR (101 MHz, CD_2Cl_2):** δ (ppm) = 186.79 (C(8)), 155.72 (C(2)), 151.10 (C(14)), 135.93 (C(4)), 134.97 (C(9)), 132.62 (2C, C(12)), 124.93 (C(6)), 123.92 (C(7)), 121.10 (C(11)), 119.39 (C(5)), 116.76 (C(10)), 112.24 (2C, C(13)), 111.96 (C(3)), 44.71 (C(16)), 40.47 (2C, C(15)), 28.26 (C(17)); ***E* isomer: ^{1}H-NMR (400 MHz, CD_2Cl_2):** δ (ppm) = 8.21 - 8.13 (m, 2H, H-C(12)), 7.67 (dd, ^3J = 7.6 Hz, ^4J = 1.4 Hz, 1H, H-C(6)), 7.48 (ddd, ^3J = 8.4 Hz, ^3J = 7.2 Hz, ^4J = 1.4 Hz, 1H, H-C(4)), 7.00 (d, ^3J = 7.4 Hz, 1H, H-C(3)), 6.89 (ddd, ^3J = 7.8 Hz, ^3J = 7.3 Hz, ^4J = 0.8 Hz, 1H, H-C(5)), 6.74 - 6.69 (m, 2H, H-C(13)), 6.42 (s, 1H, H-C(10)), 4.23 (t, ^3J = 7.4 Hz, 2H, H-C(16)), 3.60 (t, ^3J = 7.3 Hz, 7.1 Hz, 2H, H-C(17)), 3.04 (s, 6H, H-C(15)); **^{13}C-NMR (101 MHz, CD_2Cl_2):** δ (ppm) = 183.57 (C(8)), 151.73 (C(14)), 151.39 (C(2)), 135.57 (C(4)), 133.76 (C(9)), 133.03 (2C, C(12)), 124.76 (C(6)), 122.39 (C(11)), 122.30 (C(7)), 121.08 (C(5)), 120.56 (C(10)), 111.69 (2C, C(13)), 109.30 (C(3)), 47.01 (C(16)), 40.50 (2C, C(15)), 28.28 (C(17)); **MS (EI+, 70 eV):** *m*/*z* (%) = 373.1 (23), 372.1 (100), 371.1 (29), 370.1 (95), 292.2 (16), 291.2 (27), 290.2 (60), 289.2 (21), 288.1 (29), 287.1 (12), 278.1 (10), 277.1 (53), 275.1 (10), 273.1 (11), 264.1 (16), 261.1 (12), 248.1 (26), 247.1 (21), 233.1 (18), 232.1 (15), 220.1 (20), 219 (17), 159.1 (12), 145.1 (10), 144.1 (12), 138.1 (18); **HRMS (EI+, $C_{19}H_{19}BrN_2O$):** calcd.: 372.0660; found: 372.0710(M+).

(*Z*/*E*)-1-(3-bromopropyl)-2-((2,3,6,7-tetrahydro-1*H*,5*H*-pyrido[3,2,1-ij]quinolin-9-yl)methylene)indolin-3-one (8)

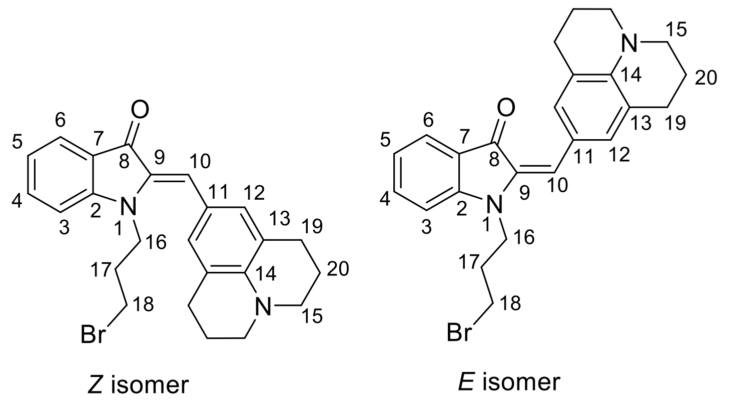

To a sealable, N_2 purged round bottom flask, equipped with a magnetic stirring bar, (*Z*)-2-((2,3,6,7-tetrahydro-1*H*,5*H*-pyrido[3,2,1-ij]quinolin-9-yl)methylene)indolin-3-one **5** (0.050 g, 0.158 mmol, 1.0 equiv.) sodium hydride (60% w / w, 0.008 g, 0.190 mmol, 1.2 equiv.) and dimethylformamide (1.6 mL, 0.1 M) were added and the deep green solution was stirred for 15 min at 23 °C. The mixture was taken up in a syringe and added via syringe pump (0.25 mL/h) to another N_2 purged round bottom flask equipped with a magnetic stirring bar filled with 1,3-dibromopropane (0.038 g, 0.190 mmol, 1.2 equiv.) and dimethylformamide (1.6 mL, 0.1 M) at 23 °C. After completion of the addition, the mixture was neutralized with aq. sat. ammonium chloride solution, extracted with ethyl acetate, washed ten times with water and treated with brine once. The combined organic phases were dried over sodium

sulfate and the volatiles were removed *in vacuo*. Subsequent purification by flash column chromatography (aluminium(III) oxide, *Brockmann*(III), hexanes / ethyl acetate, 8 / 2, v / v) yielded (*Z*/*E*)-1-(3-bromopropyl)-2-((2,3,6,7-tetrahydro-1*H*,5*H*-pyrido[3,2,1-ij]quinolin-9-yl)methylene)indolin-3-one **8** (0.032 g, 0.073 mmol, 46%) as deep violet solid. Further purification was carried out via preparative HPLC (*Machery-Nagel* VP 250/21 *NUCLEODUR Sphinx* RP 5 µm column, acetonitrile / water, 9 / 1, v / v, 15 mL/min, 35 °C column temperature, retention times: 7.6 min, 18 mg (26%) of an *E*/*Z* mixture was obtained.

***R*$_f$** = 0.29 (silica, hexanes / ethyl acetate, 8 / 2, v / v); ***Z* isomer: ^{1}H-NMR (400 MHz, CD_2Cl_2):** δ (ppm) = 7.67 (dd, ^3J = 7.6 Hz, ^4J = 1.3 Hz, 1H, H-C(6)), 7.44 (ddd, ^3J = 8.5 Hz, ^3J = 7.2 Hz, ^4J = 1.3 Hz, 1H, H-C(4)), 7.15 (d, ^3J = 8.3 Hz, 1H, H-C(3)), 6.96 (ddd, ^3J = 7.8 Hz, ^3J = 7.3 Hz, ^4J = 0.8 Hz, 1H, H-C(5)), 6.94 (s, 1H, H-C(10)), 6.91 (s, 2H, H-C(12)), 3.97 (t, ^3J = 6.7 Hz, 2H, H-C(16)), 3.24 (t, ^3J = 6.5 Hz, 4H, H-C(15)), 3.07 (t, ^3J = 6.4 Hz, 2H, H-C(18)), 2.75 (t, ^3J = 6.5 Hz, 4H, H-C(19)), 1.96 (*quin*, ^3J = 6.7 Hz, 4H, H-C(20)), 1.84 (*quin*, ^3J = 6.9 Hz, 2H, H-C(17)); **^{13}C-NMR (101 MHz, CD_2Cl_2):** δ (ppm) = 186.77 (C(8)), 155.83 (C(2)), 144.08 (C(14)), 135.59 (C(4)), 135.03 (C(9)), 130.83 (2C, C(12)), 124.67 (C(6)), 124.10 (C(7)), 121.85 (2C, C(13)), 120.56 (C(5)), 120.40 (C(11)), 117.75 (C(10)), 112.27 (C(3)), 50.46 (2C, C(15)), 44.35 (C(16)), 31.31 (C(18)), 30.40 (C(17)), 28.26 (2C, C(19)), 22.29 (2C, C(20)); ***E* isomer: ^{1}H-NMR (400 MHz, CD_2Cl_2):** δ (ppm) = 7.82 (s, 2H, H-C(12)), 7.64 (dd, ^3J = 7.6 Hz, ^4J = 1.3 Hz, 1H, H-C(6)), 7.51 (ddd, ^3J = 8.5 Hz, ^3J = 7.3 Hz, ^4J = 1.4 Hz, 1H, H-C(4)), 7.01 (d, ^3J = 8.3 Hz, 1H, H-C(3)), 6.83 (ddd, ^3J = 7.7 Hz, ^3J = 7.3 Hz, ^4J = 0.7 Hz, 1H, H-C(5)), 6.30 (s, 1H, H-C(10)), 3.99 (t, ^3J = 6.4 Hz, 2H, H-C(16)), 3.48 (t, ^3J = 6.1 Hz, 2H, H-C(18)), 3.25 (t, ^3J = 6.4 Hz, 4H, H-C(15)), 2.78 (t, ^3J = 6.5 Hz, 4H, H-C(19)), 2.27 (*quin*, ^3J = 6.6 Hz, 2H, H-C(17)), 1.97 (*quin*, ^3J = 6.6 Hz, 4H, H-C(20)); **^{13}C-NMR (101 MHz, CD_2Cl_2):** δ (ppm) = 183.25 (C(8)), 151.37 (C(2)), 144.94 (C(14)), 135.14 (C(4)), 133.58 (C(9)), 130.62 (2C, C(12)), 124.51 (C(6)), 122.22 (C(7)), 121.86 (C(10)), 121.43 (C(11)), 120.88 (2C, C(13)), 118.57 (C(5)), 109.19 (C(3)), 50.56 (2C, C(15)), 40.96 (C(16)), 31.84 (C(18)), 31.30 (C(17)), 28.31 (2C, C(19)), 22.37 (2C, C(20)); **MS (EI+, 70 eV):** *m*/*z* (%) = 439.1 (25), 438.1 (100), 437.1 (27), 436.1 (99), 357.2 (19), 356.2 (39), 355.2 (10), 329.1 (29), 315.1 (16), 288.1 (15), 287.1 (59), 186.1 (52), 173.1 (26), 164.6 (10), 164.1 (17), 150.1 (10), 57 (12), 44.0 (19); **HRMS (EI+, $C_{24}H_{25}BrN_2O$):** calcd.: 436.1150; found: 436.1146 (M+).

Synthesis of charge-tagged hemiindigos 1 - 3

1,4-Diazabicyclo[2.2.2]octane was purchased from *Sigma-Aldrich* and used as received. Hemiindigos **1** - **3** were prepared according to *B. Almarzoqui* et *al.* at elevated temperatures [3].

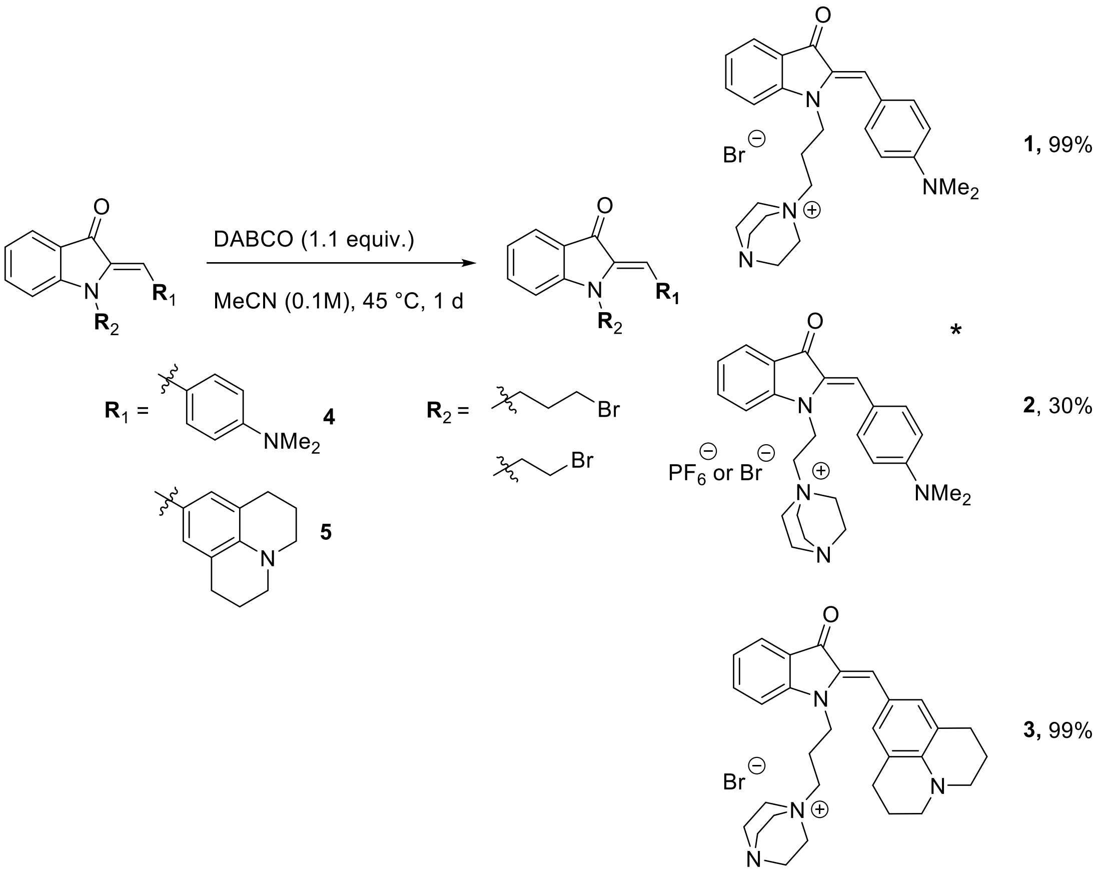

Scheme S2 Preparation of charge-tagged hemiindigos *Z/E* 1-3. 1,4-Diazabicyclo[2.2.2]octane reacts to its quaternary bromide salts with hemiindigos **6** and **8** via *Menschutkin* reaction at 45 °C within one day almost quantitatively.

* Hemiindigo **2** was prepared with 5.0 equivalents of 1,4-diazabicyclo[2.2.2]octane and showed no quantitative reaction. The hexafluorophosphate salt was prepared due to low solubility in dichloromethane with minor success, (deuterated) acetonitrile was further used for NMR and UV-Vis measurements.

(*Z*/*E*)-1-(3-(2-(4-(dimethylamino)benzylidene)-3-oxoindolin-1-yl)propyl)-1,4-diazabicyclo[2.2.2]octan-1-ium bromide (1)

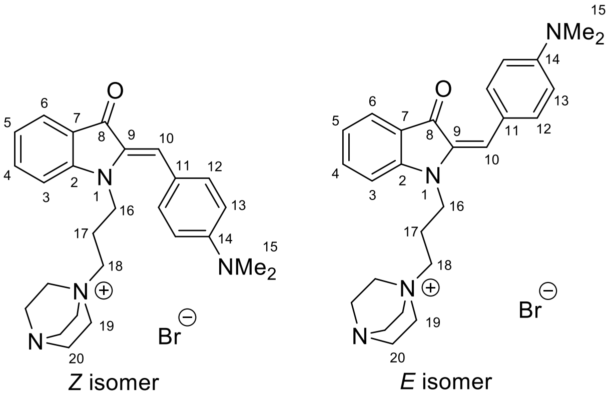

To a round bottom flask, equipped with a magnetic stirring bar and a rubber septum, (*Z*/*E*)-1-(3-bromo-propyl)-2-(4-(dimethylamino)benzylidene)indolin-3-one **6** (0.0154 g, 0.040 mmol, 1.0 equiv.), 1,4-diazabicyclo[2.2.2]octane (0.0067 g, 0.060 mmol, 1.5 equiv.) and acetonitrile (0.25 mL, 0.1 M) were added and the solution was heated to 45 °C for 1 d. The volatiles and 1,4-diazabicyclo[2.2.2]octane were removed *in vacuo* and 19.7 mg (0.0397 mmol, 99%) of (*Z*/*E*)-1-(3-(2-(4-(dimethylamino)benzylidene)-3-oxoindolin-1-yl)propyl)-1,4-diazabicyclo[2.2.2]octan-1-ium bromide XX was obtained as deep red solid.

***Z* isomer: ^{1}H-NMR (600 MHz, CD_2Cl_2):** δ (ppm) = 7.66 (ddd, ^{3}J = 7.6 Hz, ^{4}J = 1.3 Hz, ^{4}J = 0.6 Hz, 1H, H-C(6)), 7.55 (ddd, ^{3}J = 8.4 Hz, ^{3}J = 7.2 Hz, ^{4}J = 1.4 Hz, 1H, H-C(4)), 7.37 - 7.33 (m, 2H, H-C(12)), 7.34 (d, ^{3}J = 8.2 Hz, 1H, H-C(3)), 7.02 (s, 1H, H-C(10)), 6.98 (ddd, ^{3}J = 7.8, ^{3}J = 7.5, ^{4}J = 0.7 Hz, 1H, H-C(5)), 6.77 - 6.73 (m, 2H, H-C(13)), 4.02 (t, ^{3}J = 7.5 Hz, 2H, H-C(16)), 3.79 (t, ^{3}J = 7.6 Hz, 6H, H-C(19)), 3.24 - 3.20 (m, 2H, H-C(18)), 3.14 - 3.11 (m, 6H, H-C(20)), 3.02 (s, 6H, H-C(15)), 1.64 (*quin*, ^{3}J = 7.6 Hz, 2H, H-C(17)); **^{13}C-NMR (151 MHz, CD_2Cl_2):** δ (ppm) = 186.83 (C(8)), 155.42 (C(2)), 151.01 (C(14)), 136.57 (C(4)), 135.16 (C(9)), 132.71 (2C, C(12)), 124.81 (C(6)), 123.47 (C(7)), 121.12 (C(11)), 121.08 (C(5)), 116.04 (C(10)), 112.49 (C(3)), 112.20 (2C, C(13)), 62.11 (C(18)), 52.95 (3C, C(19)), 45.70 (3C, C(20)), 42.49 (C(16)), 40.55 (2C, C(15)), 19.91 (C(17)); ***E* isomer: ^{1}H-NMR (600 MHz, CD_2Cl_2):** δ (ppm) = 8.40 - 8.36 (m, 2H, H-C(12)), 7.63 (ddd, ^{3}J = 7.6 Hz, ^{4}J = 1.3 Hz, ^{4}J = 0.7 Hz, 1H, H-C(6)), 7.46 (ddd, ^{3}J = 8.3, ^{3}J = 7.1, ^{4}J = 1.4 Hz, 1H, H-C(4)), 7.20 (d, ^{3}J = 8.3 Hz, 1H, H-C(3)), 6.84 (ddd, ^{3}J = 7.8 Hz, ^{3}J = 7.2 Hz, ^{4}J = 0.6 Hz, 1H, H-C(5)), 6.78 (s, 1H, H-C(10)), 6.69 - 6.66 (m, 2H, H-C(13)), 4.06 (t, ^{3}J = 7.2 Hz, 2H, H-C(16)), 3.89 - 3.84 (m, 2H, H-C(18)), 3.50 (t, ^{3}J = 7.6 Hz, 6H, H-C(19)), 3.17 - 3.13 (m, 6H, H-C(20)), 2.99 (s, 6H, H-C(15)), 2.23 - 2.16 (m, 2H, H-C(17)). **^{13}C-NMR (151 MHz, CD_2Cl_2):** δ (ppm) = 183.75 (C(8)), 151.66 (C(14)), 151.45 (C(2)), 135.93 (C(4)), 133.90 (C(9)), 133.52 (2C, C(12)), 124.58 (C(6)), 122.81 (C(11)), 122.11 (C(7)), 121.35 (C(10)), 119.08 (C(5)), 111.60 (2C, C(13)), 109.86 (C(3)), 62.21 (C(18)), 53.25 (3C, C(19)), 45.82 (3C, C(20)), 40.44

(2C, C(15)), 39.74 (C(16)), 21.16 (C(17)); **HRMS (ESI+, $C_{26}H_{33}N_4O^+$):** calcd.: 417.26489; found: 417.26459 (M+).

(*Z*/*E*)-1-(2-(2-(4-(dimethylamino)benzylidene)-3-oxoindolin-1-yl)ethyl)-1,4-diazabicyclo-[2.2.2]octan-1-ium hexafluorophosphate (2)

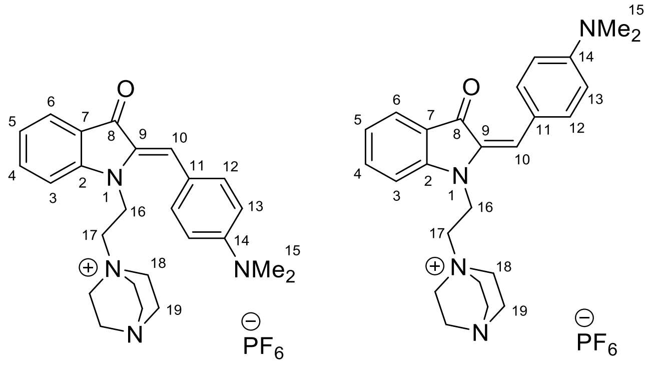

Z isomer *E* isomer

To a round bottom flask, equipped with a magnetic stirring bar and a rubber septum, (*Z*/*E*)-1-(3-bromoethyl)-2-(4-(dimethylamino)benzylidene)indolin-3-one **7** (0.036 g, 0.097 mmol, 1.0 equiv.), 1,4-diazabicyclo[2.2.2]octane (0.055 g, 0.485 mmol, 5.0 equiv.) and acetonitrile (0.1 mL, 0.1 M) were added and the solution was heated to 45 °C for 1 d. The volatiles and 1,4-diazabicyclo[2.2.2]octane were removed *in vacuo*. Water and diethyl ether were added and the extracted organic layers were combined, weighted and reused. The aqueous phase was charged onto a *Supleco* DSC-WCX ion exchange sorbent and eluted with a 30 mM water / acetonitrile (7 / 3, v / v) potassium hexafluorophosphate solution and concentrated *in vacuo*. Subtraction of the previously weighted unreacted educt yielded 0.016 g (0.029 mmol, 30% of (*Z*/*E*)-1-(2-(2-(4-(dimethylamino)benzylidene)-3-oxoindolin-1-yl)ethyl)-1,4-diazabicyclo-[2.2.2]octan-1-ium hexafluorophosphate hexafluorophosphate **2** as deep red solid with potassium hexafluorophosphate as residue.

***Z* isomer: ^{1}H-NMR (400 MHz, CD_3CN):** δ (ppm) = 7.67 (d, ^3J = 7.6 Hz, 1H, H-C(6)), 7.63 (ddd, ^3J = 8.3 Hz, ^3J = 7.2 Hz, ^4J = 1.3 Hz, 1H, H-C(4)), 7.40 - 7.35 (m, 2H, H-C(12)), 7.31 (d, ^3J = 8.3 Hz, 1H, H-C(3)), 7.07 (ddd, ^3J = 7.8, ^3J = 7.3 Hz, 0.9 Hz, 1H, H-C(5)), 7.06 (s, 1H, H-C(10)), 6.85 - 6.80 (m, 2H, H-C(13)), 4.27 (t, ^3J = 8.7 Hz, 2H, H-C(16)), 3.00 (s, 6H, H-C(15)), 2.91 (t, ^3J = 7.2 Hz, 6H, H-C(19)), 2.87 - 2.81 (m, 2H, H-C(17)), 2.77 (t, ^3J = 7.2 Hz, 6H, H-C(18)); **^{13}C-NMR (101 MHz, CD_2Cl_2):** δ (ppm) = 186.52 (C(8)), 155.15 (C(2)), 151.82 (C(14)), 136.97 (C(4)), 134.69 (C(9)), 132.93 (2C, C(12)), 124.93 (C(6)), 123.78 (C(7)), 121.93 (C(5)), 120.42 (C(11)), 117.07 (C(10)), 112.74 (2C, C(13)), 112.15 (C(3)), 59.20 (C(17)), 52.84 (3C, C(18)), 45.11 (3C, C(19)), 40.11 (2C, C(15)), 37.47 (C(16)); ***E* isomer: ^{1}H-NMR (400 MHz, CD_3CN):** δ (ppm) = 8.24 - 8.19 (m, 2H, H-C(12)), 7.64 (d, ^3J = 7.6 Hz, 1H, H-C(6)), 7.56 (ddd, ^3J = 8.4 Hz, ^3J = 7.2 Hz, ^4J = 1.3 Hz, 1H, H-C(4)), 7.11 (d, ^3J = 8.3 Hz, 1H, H-C(3)), 6.95 (ddd, ^3J = 7.8, ^3J = 7.2 Hz, ^4J = 0.6 Hz, 1H, H-C(5)), 6.79 - 6.74 (m, 2H, H-C(13)),

6.51 (s, 1H, H-C(10)), 4.29 (t, ^3J = 8.4 Hz, 2H, H-C(16)), 3.38 (t, ^3J = 7.8 Hz, 2H, H-C(17)), 3.37 (t, ^3J = 7.8 Hz, 6H, H-C(18)), 3.15 (t, ^3J = 7.1 Hz, 6H, H-C(19)), 3.03 (s, 6H, H-C(15)); **^{13}C-NMR (101 MHz, CD_2Cl_2):** δ (ppm) = 183.58 (C(8)), 152.26 (C(14)), 151.06 (C(2)), 136.25 (C(4)), 133.53 (2C, C(12)), 133.30 (C(9)), 124.77 (C(6)), 122.67 (C(7)), 122.36 (C(11)), 121.79 (C(10)), 120.06 (C(5)), 111.88 (2C, C(13)), 109.78 (C(3)), 59.58 (C(17)), 53.29 (3C, C(18)), 45.37 (3C, C(19)), 40.02 (2C, C(15)), 35.42 (C(16)); **^{19}F-NMR (376 MHz, CD_3CN):** δ (ppm) = -72.99 (d, ^1J = 706.0 Hz, 6F, F-P); **^{31}P-NMR (162 MHz, CD_3CN):** δ (ppm) = -144.67 (sept, ^1J = 706.2 Hz, 1P, P-F); **HRMS (ESI+, $C_{25}H_{31}N_4O^+$):** calcd.: 403.24924, found: 403.24925 (M+).

(*Z*/*E*)-1-(3-(3-oxo-2-((2,3,6,7-tetrahydro-1*H*,5*H*-pyrido[3,2,1-ij]quinolin-9-yl)methylene)-indolin-1-yl)propyl)-1,4-diazabicyclo[2.2.2]octan-1-ium bromide(3)

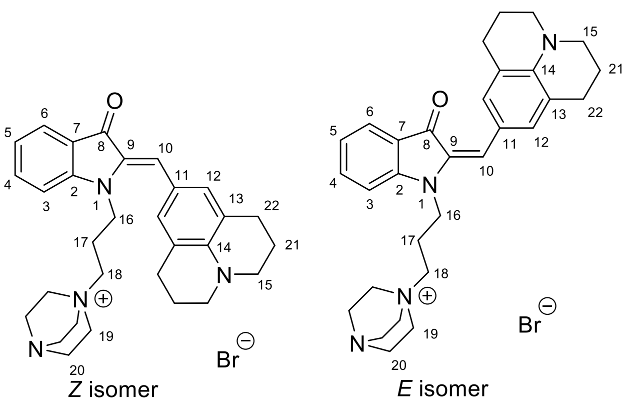

To a round bottom flask, equipped with a magnetic stirring bar and a rubber septum, (*Z*/*E*)-1-(3-bromopropyl)-2-((2,3,6,7-tetrahydro-1*H*,*5H*-pyrido[3,2,1-ij]quinolin-9-yl)methylene)indolin-3-one **8** (0.0182 g, 0.0416 mmol, 1.0 equiv.), 1,4-diazabicyclo[2.2.2]octane (0.0051 g, 0.046 mmol, 1.0 equiv.) and acetonitrile (0.42 mL, 0.1 M) were added and the solution was heated to 45 °C for 1 d. The volatiles and 1,4-diazabicyclo[2.2.2]octane were removed *in vacuo* and 22.6 mg (0.0411 mmol, 99%) of (*Z*/*E*)-1-(3-(3-oxo-2-((2,3,6,7-tetrahydro-1*H*,5*H*-pyrido[3,2,1-ij]quinolin-9-yl)methylene)-indolin-1-yl)propyl)-1,4-diazabicyclo[2.2.2]octan-1-ium bromide **3** was obtained as deep violet solid.

***Z* isomer: ^{1}H-NMR (600 MHz, CD_2Cl_2):** δ (ppm) = 7.66 (dd, ^3J = 7.6 Hz, ^4J = 1.2 Hz, 1H, H-C(6)), 7.54 (ddd, ^3J = 8.4 Hz, ^3J = 7.2 Hz, ^4J = 1.4 Hz, 1H, H-C(4)), 7.32 (d, ^3J = 8.3 Hz, 1H, H-C(3)), 6.98 (ddd, ^3J = 7.8 Hz, ^3J = 7.3, ^4J = 0.8 Hz, 1H, H-C(5)), 6.96 (s, 1H, H-C(10)), 6.91 (s, 2H, H-C(12)), 4.05 (t, ^3J = 7.5 Hz, 2H, H-C(16)), 3.26 - 3.24 (m, 4H, H-C(15)), 3.23 - 3.20 (m, 2H, H-C(18)), 3.18 (t, ^3J = 7.5 Hz, 6H, H-C(19)), 3.04 (t, ^3J = 7.7 Hz, 6H, H-C(20)), 2.75 (t, ^3J = 6.2 Hz, 4H, H-C(22)), 1.98 - 1.94 (m, 4H, H-C(21)), 1.65 (*quin*, ^3J = 8.0 Hz, 2H, H-C(17)); **^{13}C-NMR (151 MHz, CD_2Cl_2):** δ (ppm) =

186.65 (C(8)), 155.29 (C(2)), 144.32 (C(14)), 136.22 (C(4)), 134.45 (C(9)), 130.64 (2C, C(12)), 124.39 (C(6)), 123.93 (C(7)), 121.58 (2C, C(13)), 121.06 (C(5)), 119.96 (C(11)), 117.52 (C(10)), 112.71 (C(3)), 62.16 (C(18)), 52.94 (3C, C(19)), 50.43 (2C, C(15)), 45.82 (3C, C(20)), 42.60 (C(16)), 28.23 (2C, C(22)), 22.17 (2C, C(21)), 19.78 (C(17)); ***E* isomer: ^{1}H-NMR (600 MHz, CD_2Cl_2):** δ (ppm) = 7.99 (s, 2H, H-C(12)), 7.62 (dd, ^{3}J = 7.6 Hz, ^{4}J = 1.3 Hz, 1H. H-C(6)), 7.45 (ddd, ^{3}J = 8.3 Hz, ^{3}J = 7.2 Hz, ^{4}J = 1.4 Hz, 1H, H-C(4)), 7.20 (d, ^{3}J = 8.3 Hz, 1H, H-C(3)), 6.83 (^{3}J = 7.7 Hz, ^{3}J = 7.3 Hz, ^{4}J = 0.6 Hz, 1H, H-C(5)), 6.59 (s, 1H, H-C(10)), 4.03 (t, ^{3}J = 7.4 Hz, 2H, H-C(16)), 3.84 - 3.77 (m, 2H, H-C(18)), 3.50 (t, ^{3}J = 8.3 Hz, 6H, H-C(19)), 3.24 - 3.22 (m, 4H, H-C(15)), 3.13 (t, ^{3}J = 7.7 Hz, 6H, H-C(20)), 2.74 (t, ^{3}J = 6.1 Hz, 4H, H-C(22)), 2.22 - 2.15 (m, 2H, H-C(17)), 1.94 - 1.90 (m, 4H, H-C(21)); **^{13}C-NMR (151 MHz, CD_2Cl_2):** δ (ppm) = 183.06 (C(8)), 150.96 (C(2)), 145.11 (C(14)), 135.53 (C(4)), 133.09 (C(9)), 131.30 (2C, C(12)), 124.70 (C(6)), 122.49 (C(10)), 122.21 (C(7)), 121.80 (C(11)), 120.79 (2C, C(13)), 118.90 (C(5)), 109.87 (C(3)), 62.25 (C(18)), 53.23 (3C, C(19)), 50.52 (2C, C(15)), 45.75 (3C, C(20)), 39.71 (C(16)), 28.25 (2C, C(22)), 22.30 (2C, C(21)), 21.26 (C(17)); **HRMS (ESI+, $C_{30}H_{37}N_4O^+$):** calcd.: 469.29619; found: 469.29606 (M+).

Theoretical methods and calculated conformer structures and energies

To assess the potential contribution of different conformations of the alkyl chain and aniline/julolidine moiety to each ATD peak, a non-exhaustive conformer search was performed using the Force Field tool in Avogadro [5]. Conformations with relative energies <10 kcal/mol were re-optimized at the DFT ωB97X-D/cc-pVDZ level of theory using the Gaussian16 package [6]. Cartesian coordinates for these structures are given in the SI Appendix. These geometries were then used to calculate collision cross section using a version of the MOBCAL package parametrized for N_2 buffer gas [7]. Vertical excitation wavelengths were determined for the conformers were determined at the df-CC2/aug-cc-pVDZ (aug-cc-pVDZ-RI auxiliary basis set) level of theory using the MRCC program [8]. Figures S1, S2 and S3 show the optimized three-dimensional structures of *Z* and *E* conformers of hemiindigos **1**, **2** and **3**, respectively. Calculated energies, vertical excitation wavelengths and collision cross-sections for these conformers are presented in Table S1.

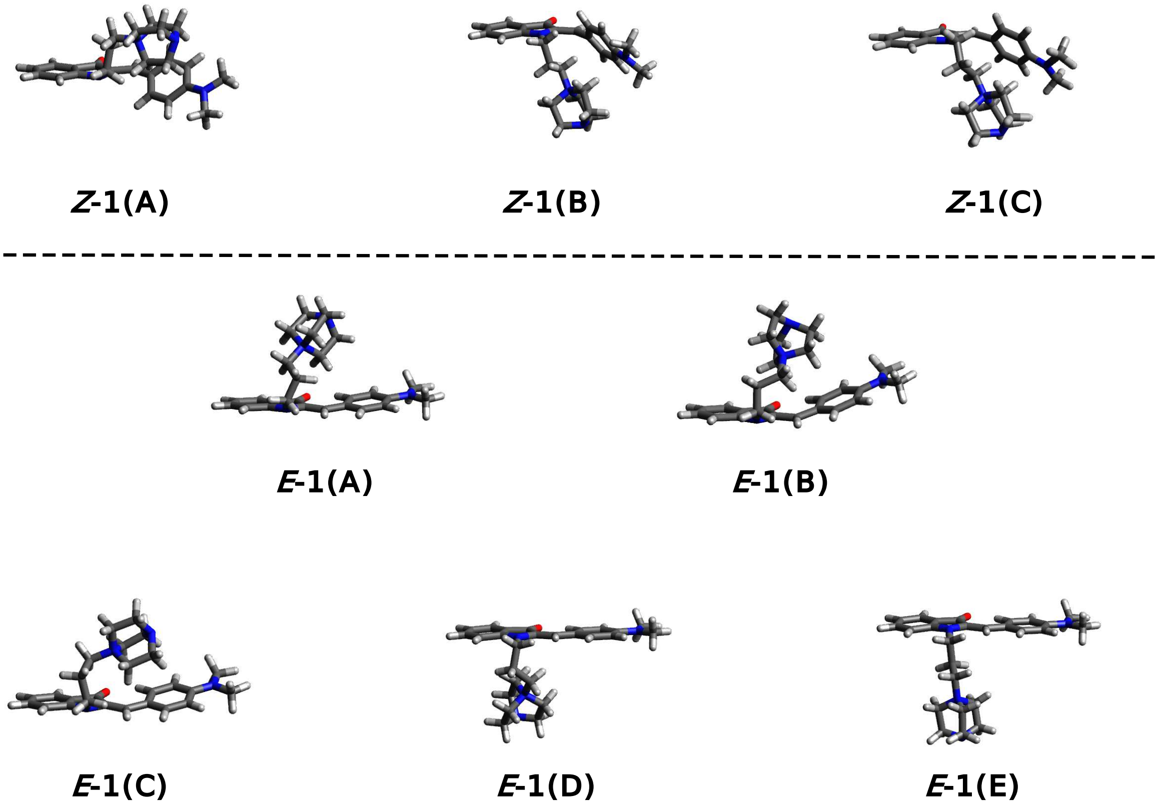

Figure S1 Optimized structures for *Z* (upper) and *E* (lower) conformers of HI **1**, computed at the ωB97X-D/cc-pVDZ level of theory.

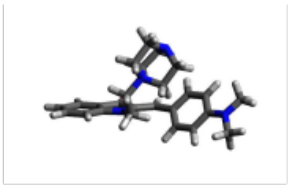

Z-2(A)

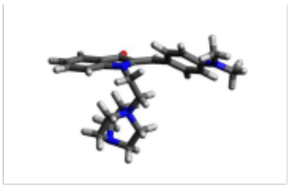

Z-2(B)

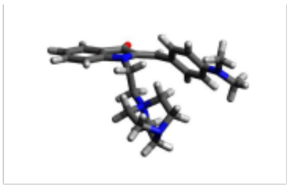

Z-2(C)

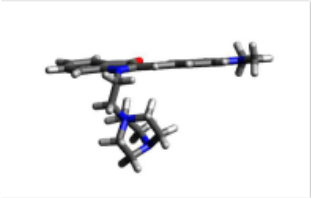

E-2(A)

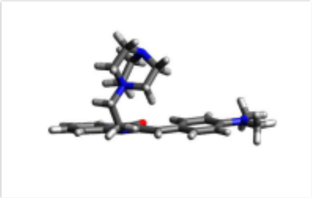

E-2(B)

Figure S2 Optimized structures for *Z* (upper) and *E* (lower) conformers of HI **2**, computed at the ωB97X-D/cc-pVDZ level of theory.

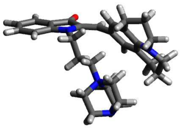

Z-3(A)

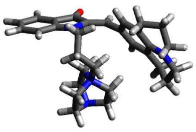

Z-3(B)

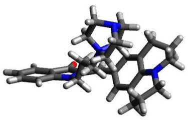

Z-3(C)

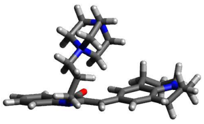

E-3(A)

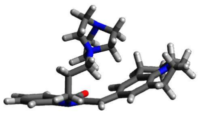

E-3(B)

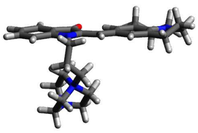

E-3(C)

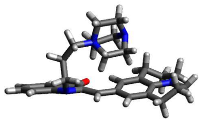

E-3(D)

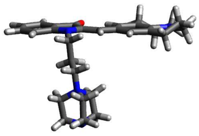

E-3(E)

Figure S3 Optimized structures for *Z* (upper) and *E* (lower) conformers of HI **3**, computed at the DFT ωB97X-D/cc-pVDZ level of theory.

Table S1 Optimized ground state energies, wavelengths and oscillator strengths for vertical $S_1 \leftarrow S_0$ and $S_2 \leftarrow S_0$ transitions, and calculated collision cross sections in N_2 buffer gas for a series of *Z/E* conformers of hemiindigos **1-3** shown in Figure S1-3. The energies were computed at the DFT ωB97X-D/cc-pVDZ level of theory. Collision cross sections were calculated using the MOBCAL program with appropriate parameter for N_2 buffer gas [7]. Vertical excitation wavelengths for $S_1 \leftarrow S_0$ and $S_2 \leftarrow S_0$ transitions were calculated at the df-CC2/aug-cc-pVDZ (aug-cc-pVDZ-RI auxiliary basis set) level of theory using the MRCC program, with the italic numbers indicated in brackets to the corresponding oscillator strengths obtained from the CIS wavefunctions [8].

Hemiindigo	Conformer	Optimized energy [kcal/mol]	Vertical excitation wavelength [nm]† $S_1 \leftarrow S_0 \Vert S_2 \leftarrow S_0$	CCS [Å^2]
	A	0.0	409 *(0.4)* $\Vert$ 364 *(0.2)*	206
1-*Z*	B	1.7	405 *(0.3)* $\Vert$ 362 *(0.0)*	214
	C	2.9	398 *(0.3)* $\Vert$ 355 *(0.0)*	211
	A	2.3	506 *(0.8)* $\Vert$ 399 *(0.4)*	216
	B	3.9	497 *(0.7)* $\Vert$ 393 *(0.4)*	211
1-*E*	C	3.9	517 *(0.8)* $\Vert$ 387 *(0.3)*	218
	D	4.0	502 *(0.7)* $\Vert$ 396 *(0.4)*	206
	E	11.3	478 *(0.8)* $\Vert$ 367 *(0.0)*	233
	A	0.0	405 *(0.5)* $\Vert$ 365 *(0.0)*	208
2-*Z*	B	1.5	440 *(0.8)* $\Vert$ 385 *(0.1)*	213
	C	1.9	398 *(0.5)* $\Vert$ 366 *(0.1)*	211
2-*E*	A	4.4	504 *(0.9)* $\Vert$ 391 *(0.2)*	213
	B	4.5	504 *(0.9)* $\Vert$ 390 *(0.2)*	213
	A	0.0	419 *(0.4)* $\Vert$ 372 *(0.0)*	222
3-*Z*	B	1.5	439 *(0.5)* $\Vert$ 374 *(0.0)*	221
	C	2.0	418 *(0.5)* $\Vert$ 371 *(0.0)*	225
	A	1.4	543 (0.9) $\Vert$ 417 *(0.3)*	225
	B	2.6	527 *(0.7)* $\Vert$ 415 *(0.3)*	220
3-*E*	C	3.5	559 *(0.9)* $\Vert$ 402 *(0.4)*	231
	D	5.3	515 *(0.9)* $\Vert$ 417 *(0.2)*	218
	E	11.2	518 *(0.9)* $\Vert$ 397 *(0.0)*	247

† oscillator strengths given in parentheses

Gas phase photoisomerization experiments

The photoisomerization of the isolated charge-tagged hemiindigos **1-3** was investigated using a homebuilt tandem ion-mobility mass spectrometer (IMS) [4]. The principle of ion-mobility spectrometry rests on the spatial and temporal separation of charged molecular isomers due to differences in their drift velocity (v_d) as they travel through buffer gas under propelled by an electric field (E).

$$v_d = K.E \qquad \text{(eq. 1)}$$

The mobility K can be expressed by the *Mason-Schamp* equation (eq. 2):

$$K = \frac{3ze}{16N}\sqrt{\frac{2\pi}{\mu k_b T}}\left(\frac{1}{\Omega}\right) = \frac{l^2}{t_d V} \qquad \text{(eq. 2)}$$

Here, z is the ion's charge number, e the electron charge, N the density of the buffer gas, μ the reduced mass of the collision partners, k_b the Boltzmann constant, l is the length of the drift region, t_d is the drift time, and V is total voltage applied across the drift region. Ω is the integral collision cross section, which depends on the interaction between ion and buffer gas molecule, and is therefore influenced by the structure of the molecular ion. Bulky, unfolded molecular ions have larger collision cross sections and therefore drift more slowly (larger t_d) than more compact molecular ions.

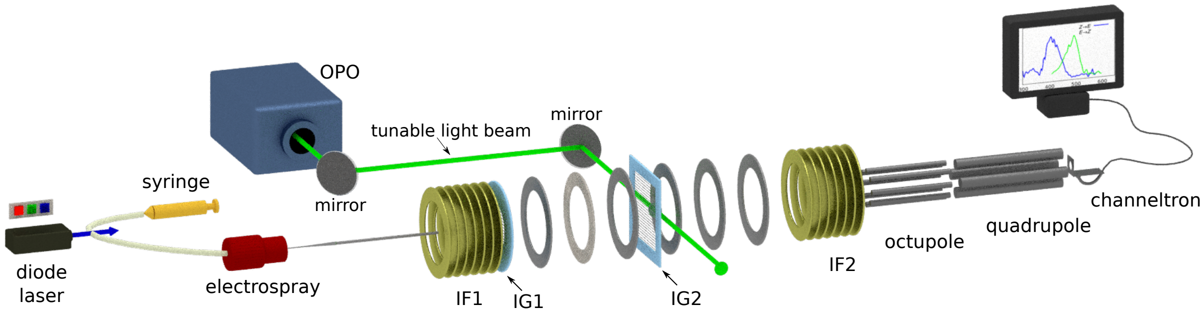

Figure S4 Tandem ion mobility mass spectrometer. Further details can be found in ref. [4].

Figure S4 shows the experimental arrangement. Hemiindigos **1 - 3** were electrosprayed from ~0.1 mM solutions of the respective precursor in acetonitrile. The ions were then transferred through a heated capillary into a first radiofrequency (RF) driven ion funnel (IF1) where they were confined radially. No significant effect of the RF drive voltage amplitude on the relative peak intensities in the arrival time distributions was observed. The absence of collision induced rearrangements suggests the existence of substantial isomerization barriers for all three compounds. After confinement by the first ion funnel, the ions were injected as packets into a two-stage drift region filled with N_2 buffer gas at ≈6.2 Torr using an electrostatic ion gate (IG1). The ion gate was pulsed open for 120 µs at a rate of 40 Hz. The drift region consists of a series of ring electrodes which established an electric field (E = 44 V/cm) that propelled the ions through the buffer gas. The ions passed through a second Bradbury-Nielsen ion gate (IG2) that

could either be held open or opened momentarily to select target isomer ions which successively passed through the second ion mobility stage, through an ion funnel, an octupole ion guide, and a quadrupole mass filter before being sensed by a Channeltron ion detector connected to a multichannel scaler. Arrival time distributions (ATDs) were obtained by plotting ion count against arrival time.

Example ATDs for hemiindigos **1-3** are shown in Figure S5. Two baseline resolved peaks were obtained for hemiindigo **2** in N_2, whereas only one broad peak was observed for compounds **1** and **3** with N_2 buffer gas (see Figure S5, upper row). Better separation was achieved by seeding the N_2 buffer gas with ≈1% 2-propanol (Figure S5, lower row). This allowed separation of the *E* and *Z* isomers for hemiindigos **1** and **3**, allowing individual isomers to be isolated and irradiated.

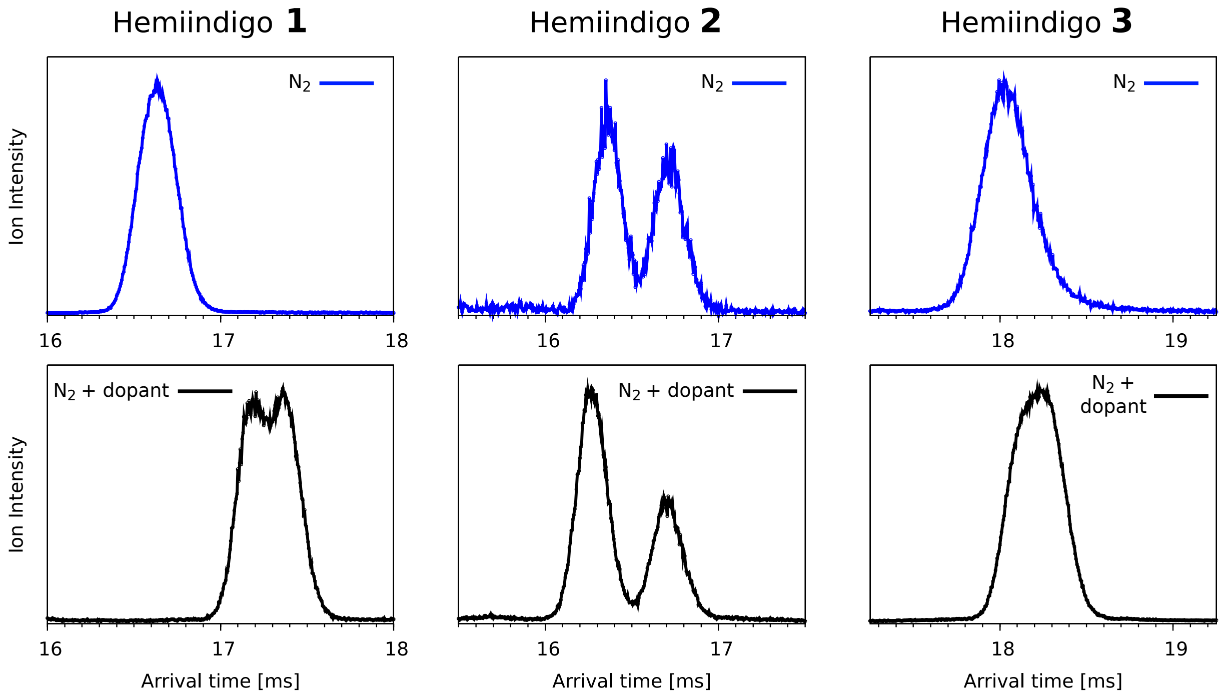

Figure S5 Arrival time distributions (ATDs) for the three investigated hemiindigo ions recorded with N_2 buffer gas (upper row) and with N_2 + 1% 2-propanol dopant (lower row).

ATD peak assignments and determination of isomer yields upon irradiation in solution

To assign the ATD peaks to specific isomers, a series of experiments were carried out in which the hemiindigo solutions in the syringe connected to the electrospray source were irradiated by visible light. ATDs were monitored after exposure of the sample in the syringe to the output of either a blue (*Laserglow LRS-473-TM-30-5*, 39.5mW, 430-473nm), green (*Thorlabs CPS533*, 4.5mW, 532nm) or red (*Melles Griot He-Ne Laser, 25-LHP-151-249*, <15mW, 632.8nm) CW laser for 5-10 minutes that served to establish a photostationary state (PSS). These ATDs are compared to ATDs obtained using solutions protected from light (see Figure 2 in manuscript). The effects of irradiating the samples on ATD peak intensities are apparent in Figure S6, where there is clear evidence for the interconversion of *Z* and *E* isomers with the relative isomer abundances depending on wavelength. The measured ATDs were fitted by the sum of two Gaussian functions having equal widths to estimate the relative isomer abundances.

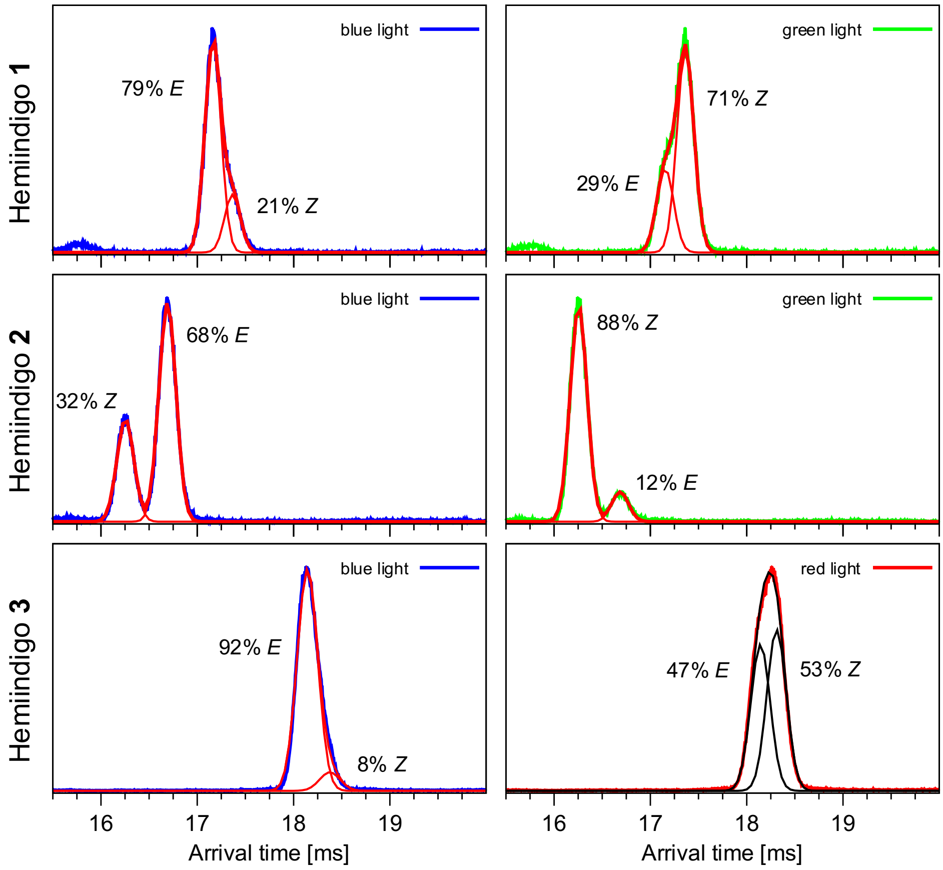

Figure S6 Fitted arrival time distributions (ATDs) for **HI 1-3** ions obtained using electrosprayed solutions exposed to light of different wavelengths. The left column shows ATDs obtained after 5 minutes exposure of the respective sample to blue light (430-480 nm) prior to electrospray ionization, whereas the right column shows ATDs obtained following exposure of the solution to green light (532 nm) or red light (632.8 nm). The fitted contributions of each isomer are given.

The isomer PSS abundances derived from the ATDs are consistent with abundances measured in solution through analysis of UV-vis spectra (see Table S2).

Photoisomerization action spectroscopy experiments

To investigate the photoisomerization of the hemiindigo ions in the gas phase, a particular isomer was selected using a pulsed Bradbury-Nielsen ion gate (IG2) situated midway along the drift region which was opened for 120 μs at an appropriate delay with respect to IG1. As shown in Figure S4, shortly after passing through the gate, the ions were exposed to a light pulse from a tuneable optical parametric oscillator (OPO, EKSPLA NT342B, 20 Hz, 5 ns pulse width). The photoisomers were separated from the parent isomers in the second stage of the drift region and were then guided through a second ion funnel (IF2) followed by a differentially pumped octupole ion guide, a quadrupole for mass selection and a Channeltron detector. The OPO operated at 20 Hz and overlapped alternate ion packets allowing 'laser on' and 'laser off' ATDs to be collected, the difference between which reflects the effect of light on the parent cation. Thus, a given photo-isomer appeared as a separate peak in the 'laser on' ATD.

Power dependence of the photoisomerization yield

To evaluate the effect of light intensity on the photoisomerization yield, the *Z* isomer of hemiindigos **1-3** were exposed to blue light (430-480 nm) over a range of fluences. Resulting power dependence plots are shown in Figure S7. The photoisomer yield is directly proportional to light fluence for hemiindigos **1** and **2**, consistent with a single-photon isomerization. For hemiindigo **3**, the linear dependence is not followed at fluences exceeding 1 mJ/pulse/cm^2, indicating the onset of saturation/multiphoton processes. Subsequent photoisomerization measurements were performed at fluences below 0.8 mJ/pulse/cm^2. Although power dependence measurements were not performed for *E*⟶*Z* photoisomerization, we expect similar power dependences to the *Z*⟶ *E* processes.

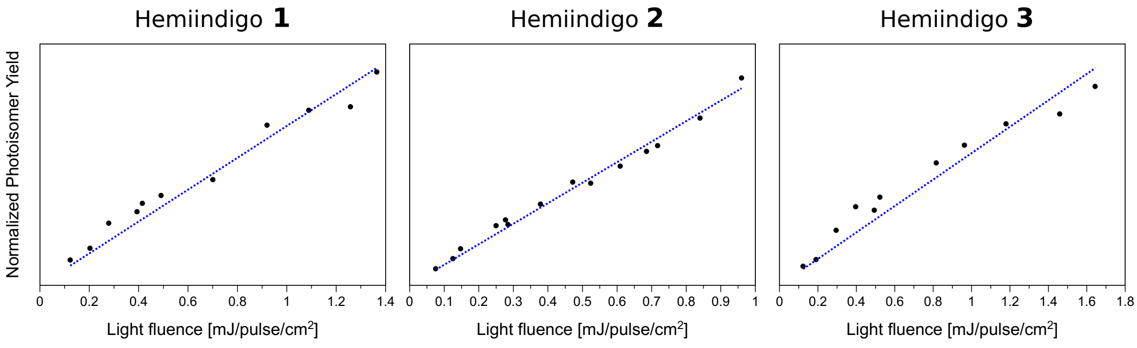

Figure S7 Normalized yield of *E* photoisomer as a function of light fluence. The experiments were performed at 450nm (hemiindigos **1** and **3**) and 430nm (hemiindigo **2**), respectively. Subsequent photoisomerization experiments were performed at a light fluence of <0.8 mJ/pulse/cm^2.

Solution photoisomerization experiments

Determination of the UV-vis absorption spectra of *Z* and *E* isomers

The UV-vis absorption spectra of pure *Z* and *E* isomers of **1** to **3** in acetonitrile were obtained by subtraction of one *E*/*Z*-mix spectrum with known isomer composition (previously determined by integration from ^{1}H NMR spectroscopy) from another *E*/*Z*-mix spectrum with different but also known isomer composition and subsequent multiplication with a weighting factor. Weighting was done by multiplying the first *E*/*Z*-mix spectrum with the *Z* (or *E*) isomer percentages of the second *E*/*Z*-mixture (determined via ^{1}H NMR spectroscopy) and *vice versa*. The obtained absorption spectrum of the respective pure isomer was multiplied by compensation factors to match the previously determined absorption values at isosbestic points.

This method of spectra determination relies on the following conditions:

- The system must consist of two chromophores, which interconvert without side reactions or decomposition

- The total chromophore concentration is constant during determination of isosbestic points, thus absorption spectra of mixtures result solely from the addition/subtraction of pure *E* and *Z* isomer spectra

- Distinct isosbestic points must be observed

Eq. 2 defines the spectrum S of the e.g. *E* isomer (*E*) as a matrix of colligated numeric values:

$$S(E) \; = \; S(w_{E1,2\ldots,}, a_{E1,2\ldots}) \qquad \text{(eq. 2)}$$

with w_{Ei} (wavelength in nm) as fixed experimental parameters value and a_{Ei} (absorption in a.u.) as experimental observables value representing the absorption spectrum. Eq. 3/4 define the measured *E/Z*-enriched mixture spectrum, $S_{mix}(E+/Z+)$, as a composite of pure S(*E*) and S(*Z*) spectra:

$$S_{mix}(E\,+) = S(E) \times f_1 + S(Z) \times f_2 \qquad \text{(eq. 3)}$$

$$S_{mix}(Z\,+) = S(E) \times f_3 + S(Z) \times f_4 \qquad \text{(eq. 4)}$$

with f_1,… being factors to account for the concentrations of each isomer in the mixture, which were determined by NMR measurements and the corresponding magnitudes of the absorption spectra. Solving the system of linear equations for S(*E*) and S(*Z*) results in eq. 5/6:

$$S(E) = \frac{S_{mix}(Z+)\times f_2 - S_{mix}(E+)\times f_4}{f_2\times f_3 - f_1\times f_4} \qquad \text{(eq. 5)}$$

$$S(Z) = \frac{S_{mix}(Z+)\times f_1 - S_{mix}(E+)\times f_3}{f_1\times f_4 - f_2\times f_3} \qquad \text{(eq. 6)}$$

Factors f_1 - f_4 were obtained from integrated indicative signals in the ^{1}H NMR spectrum (percentage divided by 100) for the *E* or *Z* isomer in the *E* or *Z* enriched mixture according to the following matrix:

	Z isomer	*E* isomer
Z enriched mixture	f_1	f_2
E enriched mixture	f_3	f_4

The hereby determined spectra consist of 100% *E* isomer S(*E*) and 100% *Z* isomer S(*Z*), respectively.

Photoisomerization experiments in solution were conducted in NMR tubes at concentrations of ~$5\cdot10^{-3}$ M in deuterated solvents (MeCN-d_3) or in 10 mm *Hellma* quartz cuvettes at concentrations of ~$2.5\cdot10^{-5}$ M. LEDs from *Roithner Lasertechnik GmbH* and *Thorlabs GmbH* 435 nm, 257 mW; 450 nm, 151 mW; 470 nm, 127 mW; 490 nm, 101 mW; 505 nm, 79 mW; 515 nm, 66 mW; 530 nm, 94 mW; 565 nm, 190 mW; 590 nm 33mW, 595nm, 160 mW; 617 nm, 188 mW; 625 nm, 130 mW) were used for irradiation of samples. LED power and quantum yields were measured with a *Coherent* PowerMax thermal photometer and the PowerMax software.

The emission spectra of each LED are shown below. In cases where spectra are not available, the spectral full width at half maximum (FWHM) is given.

Thorlabs M365D1 365nm 190 mW

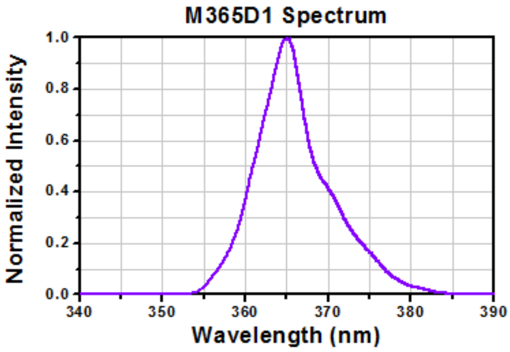

Thorlabs M385D1 385nm 270 mW

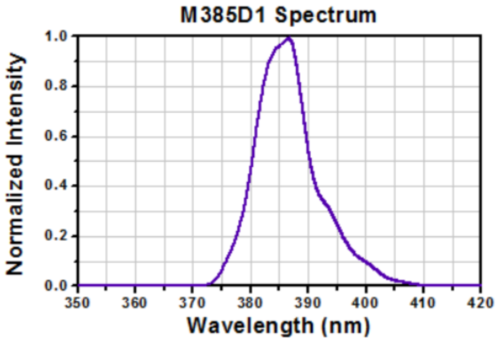

Roithner H2A1-H405 405 nm 105 mW

FWHM = 17 nm

Roithner H2A1-H420 420 nm 130 mW

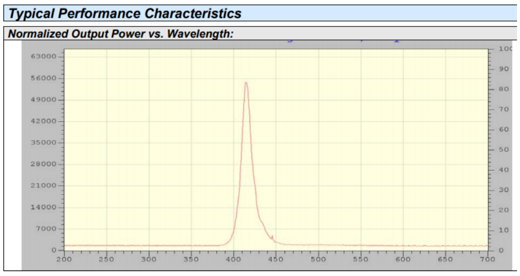

Roithner H2A1-H435 435 nm 380 mW

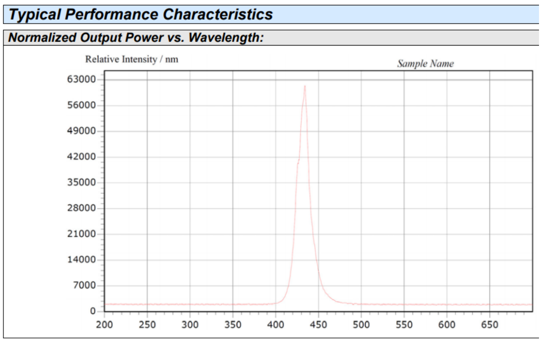

Roithner H2A1-H470 470 nm 200 mW

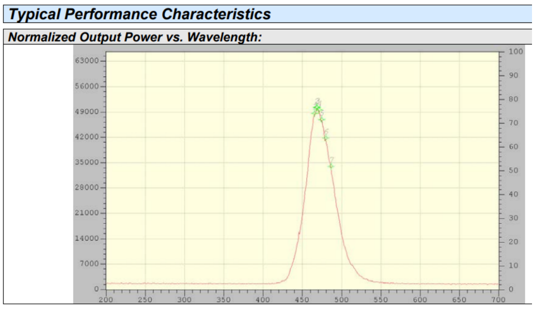

Roithner H2A1-H490 490 nm 130 mW

FWHM = 35 nm

Roithner H2A1-H505 505 nm 130 mW

FWHM = 35 nm

Roithner H2A1-H515 515 nm 150 mW

FWHM = 35 nm

Thorlabs M530D2 530nm 350 mW

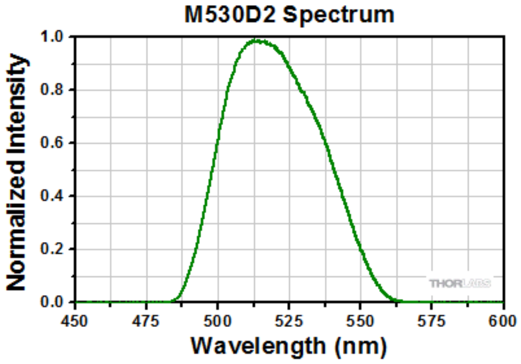

Thorlabs M565D2 565nm 880 mW

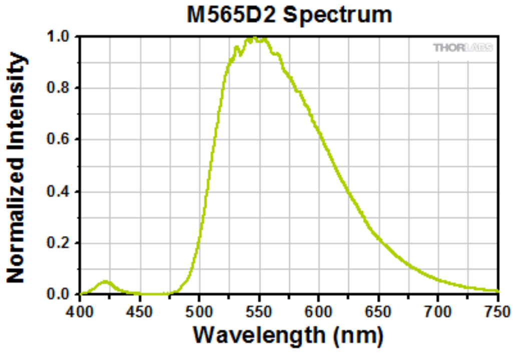

Thorlabs M590D2 590nm 160 mW

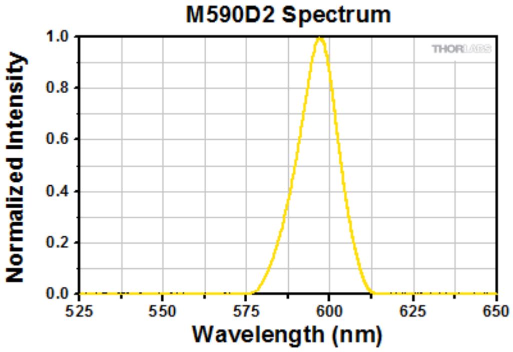

Thorlabs M595D2 595nm 445 mW

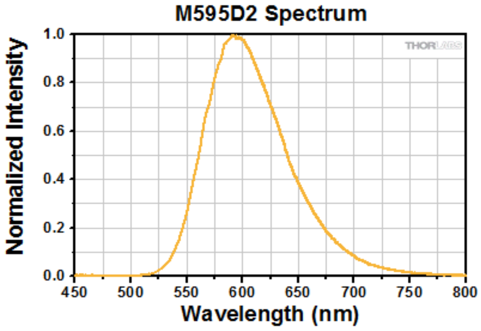

Thorlabs M617D2 617nm 600 mW

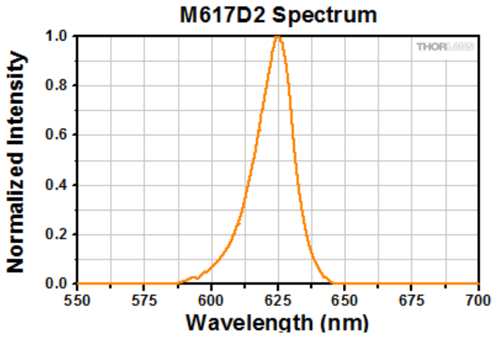

Thorlabs M625D2 625 nm, 700 mW

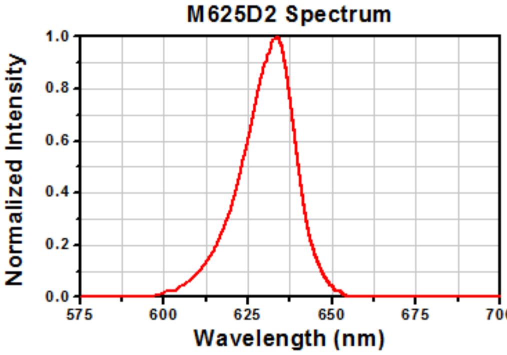

The isomeric yields obtained in the photostationary state (PSS) at different irradiation wavelengths were determined by irradiation in NMR tubes with subsequent analysis of the isomer composition by ^{1}H NMR spectroscopy or by irradiation in 10 mm quartz cuvettes followed by UV-vis measurements. In the latter case, isomer abundances were determined by first scaling obtained UV-vis spectra at the PSS to the absolute positions of previously obtained isosbestic points and then calculating the *Z*/*E*-ratio in the PSS from the known extinctions of pure isomers at distinct wavelengths aberrant to isosbestic points. Isomer abundances obtained in the PSS at different irradiation wavelengths are given in Table S2.

Table S2 Isomer yields obtained in the PSS after *Z*/*E* and *E*/*Z* photoisomerization of hemiindigos **1** - **3** in different solvents at different wavelengths. Isomer yields were determined via UV-vis measurements at ~$2.5 \cdot 10^{-5}$ M concentrations of the solutions.

Hemiindigo	Solvent	Wavelength [nm]	% *E* isomer	% *Z* isomer	Duration [min]
**1**	MeCN	450	90	10	1
		470	91	9.3	1
		490	79	21	1
		505	90	10	1
		515	67	33	1
		530	48	52	1
		565	34	66	1
		595	25	73	1
		617	1.0	99	30
**2**	MeCN	435	87	13	1
		450	95	4.7	1
		470	93	7.1	1
		490	78	22	1
		505	69	31	1
		515	44	56	1
		530	36	64	1
		565	22	78	1
		595	11	89	1
		617	1.9	98	19
**3**	MeCN	450	78	22	1
		470	93	7.1	1
		490	89	12	1
		505	83	11	1
		515	89	17	1
		530	74	26	1
		565	52	48	1
		595	18	82	1
		617	2.0	98	1
		625	1.0	99	12

PSS UV-Vis spectra

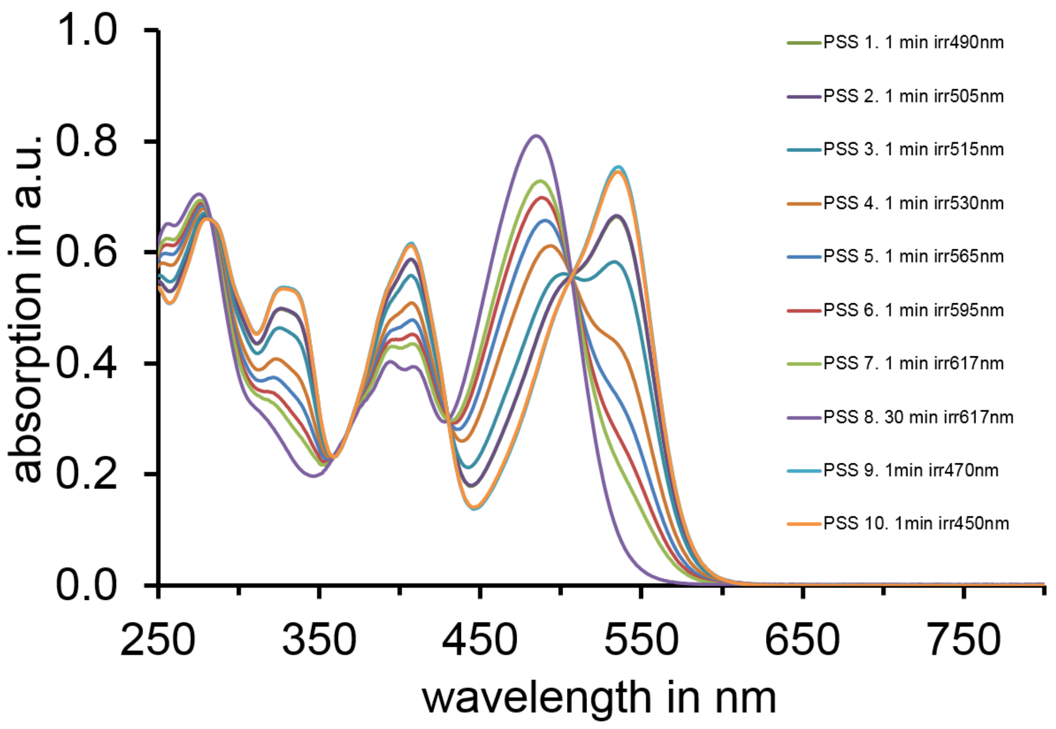

Figure S8 PSS UV-Vis spectra at different irradiation wavelengths for **1** *Z*/*E* in acetonitrile.

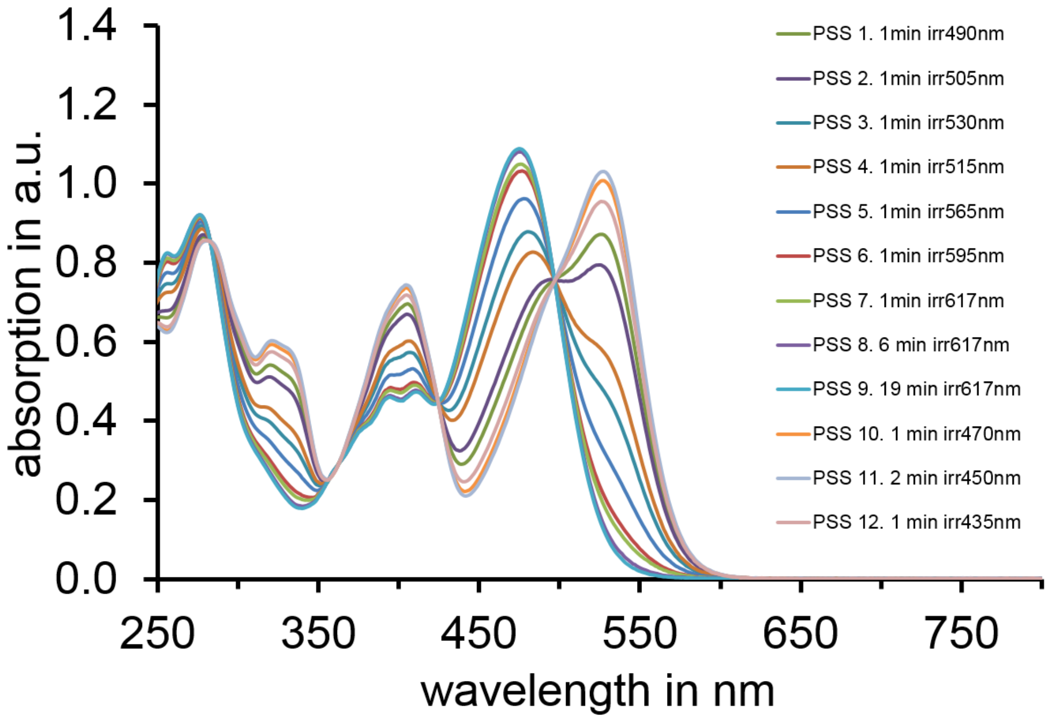

Figure S9 PSS UV-Vis spectra at different irradiation wavelengths for **2** *Z*/*E* in acetonitrile.

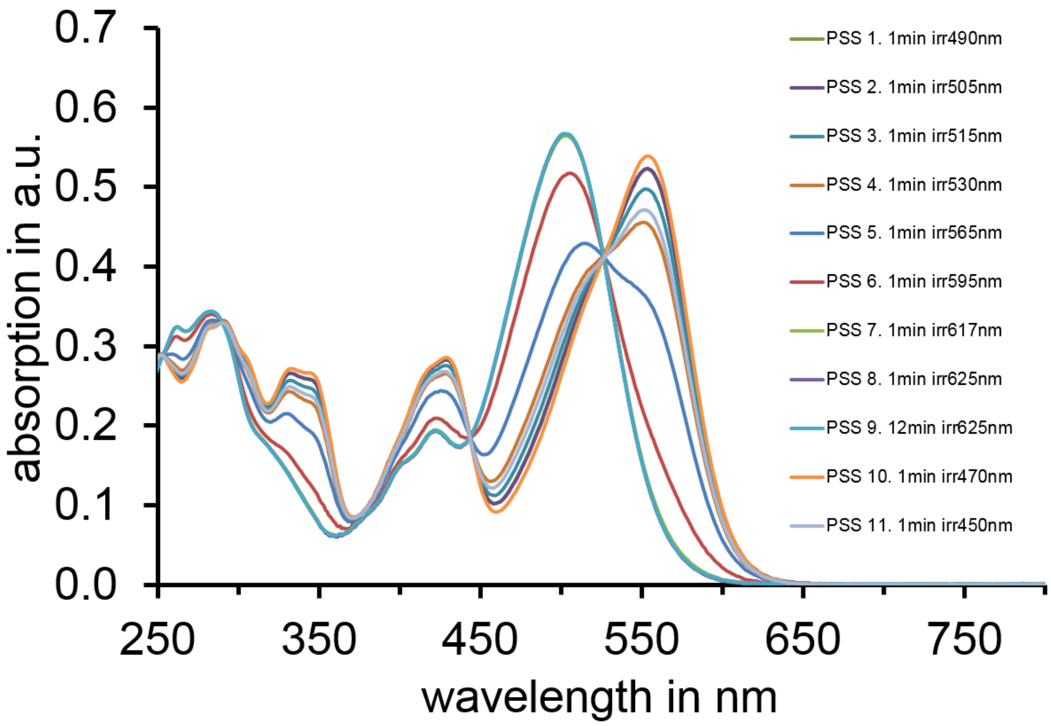

Figure S10 PSS UV-Vis spectra at different irradiation wavelengths for **3** *Z*/*E* in acetonitrile.

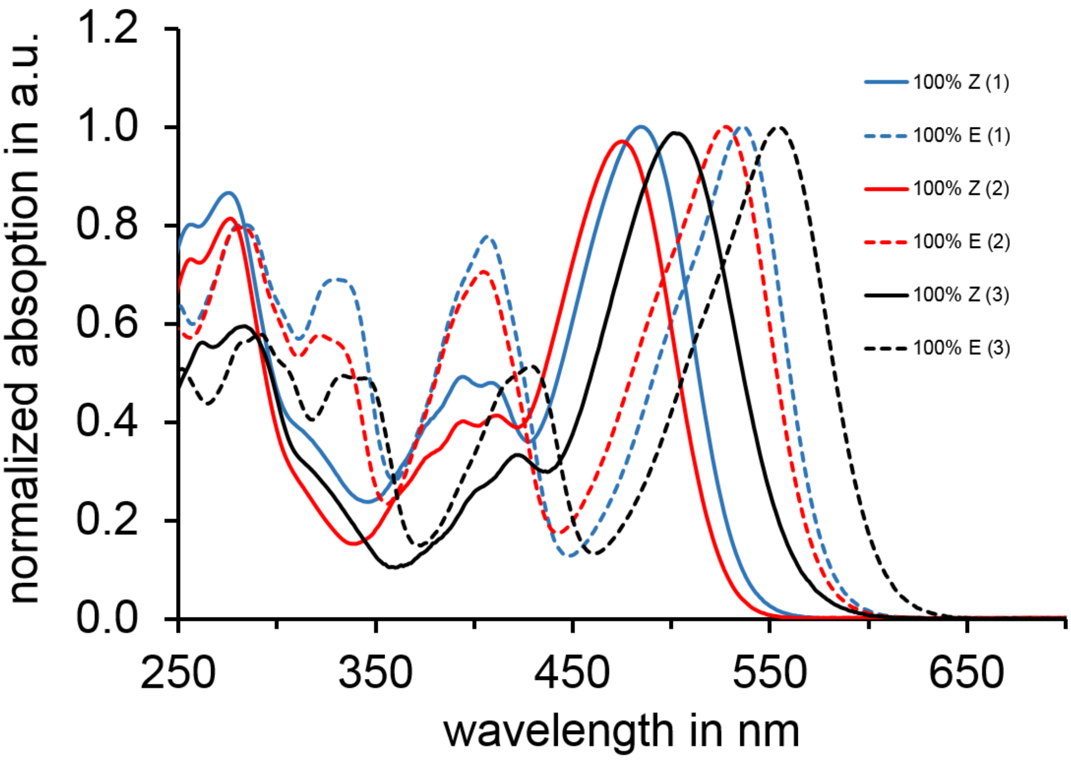

Figure S11 Absorbance normalized 100% *Z*/*E* UV-Vis spectra for **1** (blue), **2** (red) and **3** (black) in acetonitrile.

Comparison of chain length effects via NMR

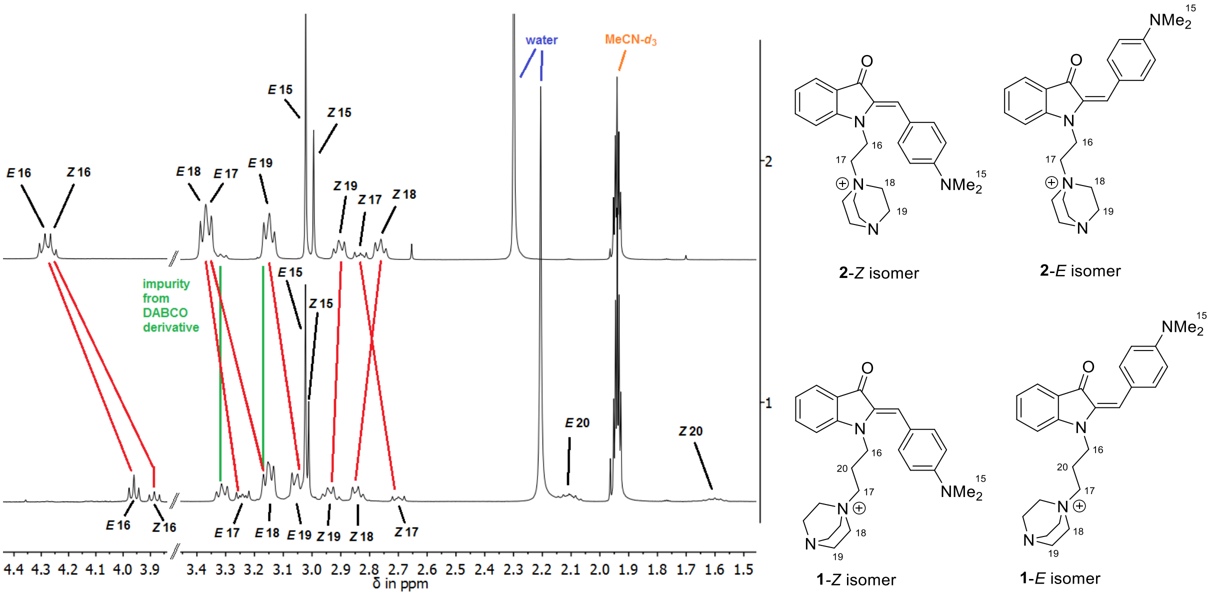

Figure S12 ^{1}H-NMR spectra of **2** (top) and **1** (bottom) in acetonitrile-d_3 (only the aliphatic section is shown). Deshielding occurs upon *Z* to *E* isomerization for both molecules with downfield shifts ranging from 0.2 to 0.7 ppm. Compared to HI **1,** the shift for signals 19 18 and 17 between *Z* and *E* isomers is significantly more pronounced for the shorter chain length (HI **2**), a fact that can be attributed to the proximity towards the electron-withdrawing hemiindigo chromophore, especially for the *E* isomer. The electron-rich stilbene moiety can donate electron density in the *Z* state towards the carbonyl function, which is impeded in the *E* state and compensated by electron-withdrawal from the substituted alkyl chain. Deshielding of all signals except for protons *Z* 19 and *Z* 18 can be observed from the HI **1** to the HI **2** spectrum. The slight shielding of DABCO protons 18 and 19 for molecule HI **2** can be explained by increasing proximity of the electron-rich stilbene part ring-current for the short chain of *Z* **2** compared to *Z* **1**. Protons *Z* 20 and *E* 20 of the three-membered chain in HI **1** seem to disengage this donor acceptor setup, keeping the chain flexible.

Conformational analysis in solution

Aromatic/aliphatic NOEs of HI 1

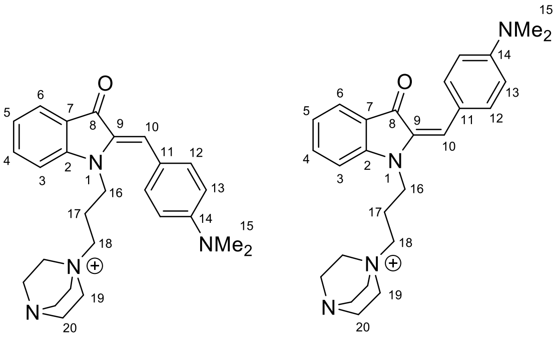

Z isomer *E* isomer

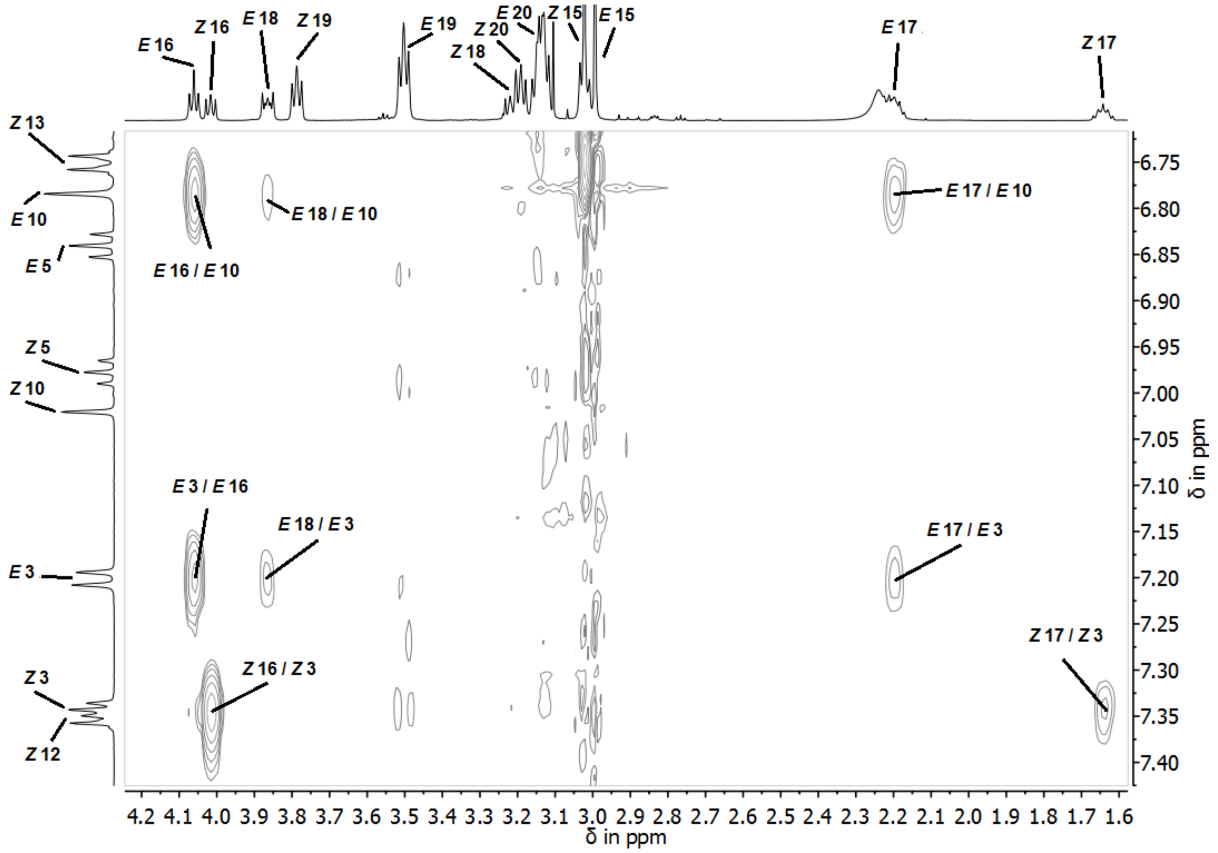

Figure S13: Section of the 2D NOESY NMR spectrum (CD_2Cl_2, 600 MHz, 27 °C) of HI **1**. The strong NOE cross signals between protons *E* 16/17 / *E* 10 and the weak signal of *E* 18 indicates their assignment to the *E* isomer to be correct. *Z* 10 shows no cross peaks with the chain protons. *Z* 16/17 show overlapping cross signals with protons *Z* 3/12, disentanglement determines a preferred conformer population in proximity of proton 3.

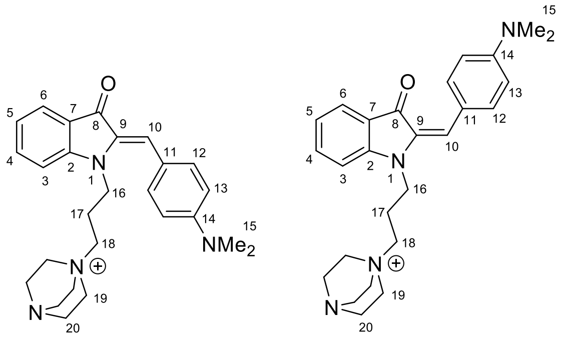

Z isomer *E* isomer

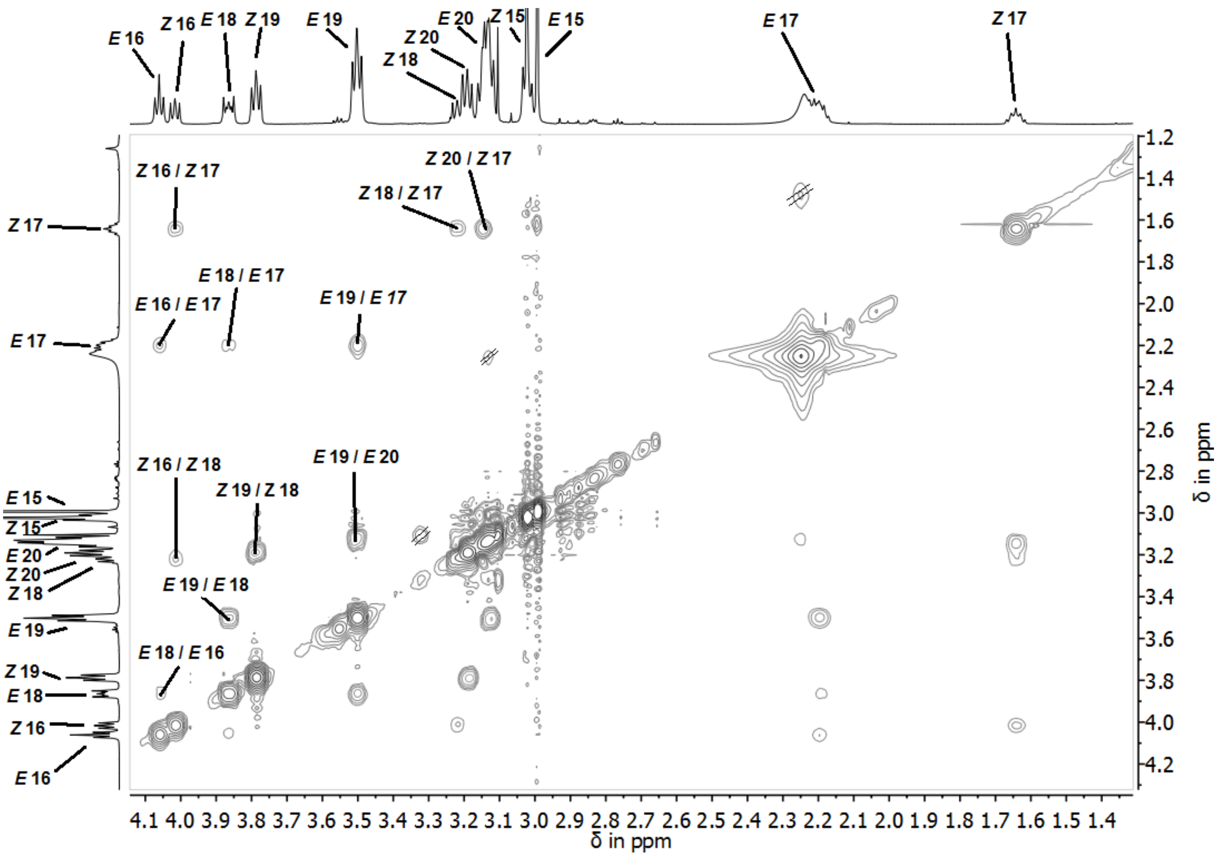

Figure S14: Section of the 2D NOESY NMR spectrum (CD_2Cl_2, 600 MHz, 27 °C) of HI **1**. The expected signals for the alkyl chain and DABCO are identified.

Aromatic/aliphatic NOEs of HI 2

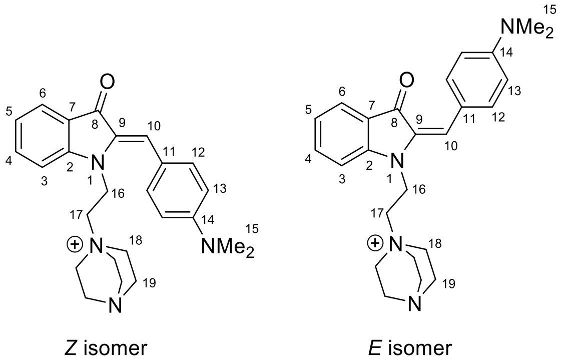

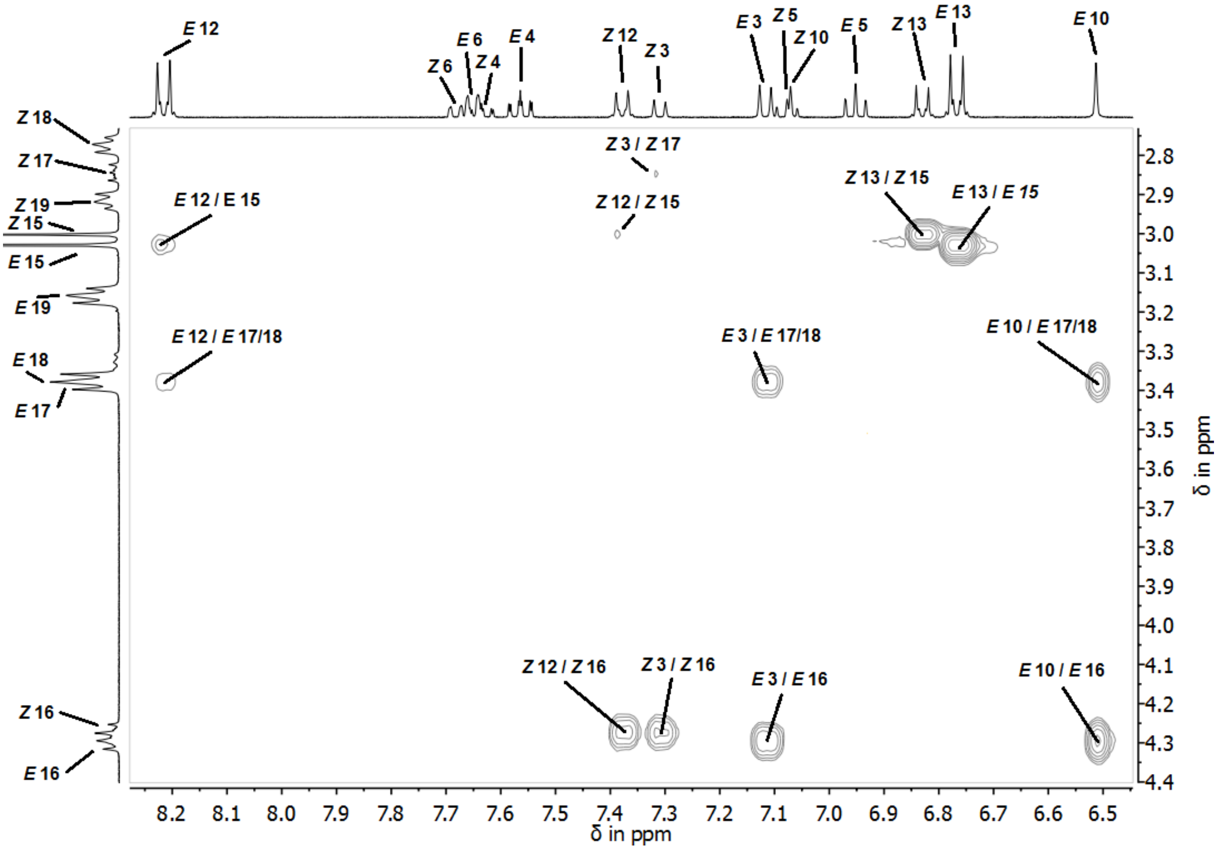

Figure S15: Section of the 2D NOESY NMR spectrum (MeCN-d_3, 400 MHz, 27 °C) of HI **2**. Strong cross signals of *E* 10 with protons *E* 16 and overlapped protons *E* 17/18 constitute to their correct assignment to the *E* isomer. *Z* 10 shows no NOEs with alkyl chain protons. *Z* 12 shows strong signals with proton Z 16 but no cross signal with Z 17/18/19 while proton *Z* 3 shows signals with *Z* 16 and *Z* 17. This suggests a preferred chain conformer population in proximity of proton 3, although in a less pronounced manner than observed for *Z*-**1**. *E* 12 exhibits weak overlapped cross signals for protons *E* 17/18 which cannot be seen for *E* 18/19 in HI *E*-**1** as the elongated chain negates NOE significant signals to the peripheral DABCO tag.

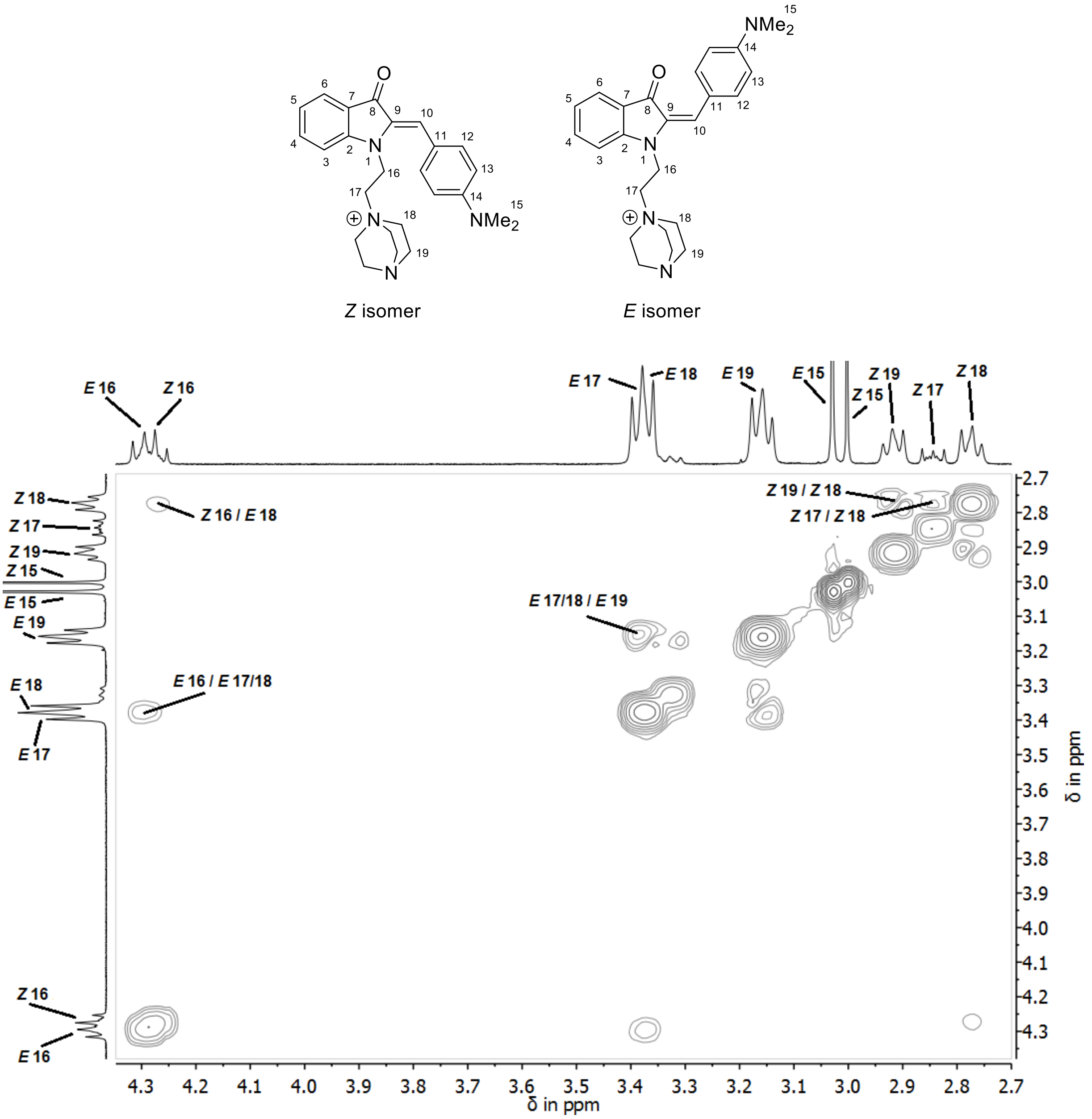

Figure S16: Section of the 2D NOESY NMR spectrum (MeCN-d_3, 400 MHz, 27 °C) of HI **2**. The expected signals for the alkyl chain and DABCO are identified.

NMR Spectra

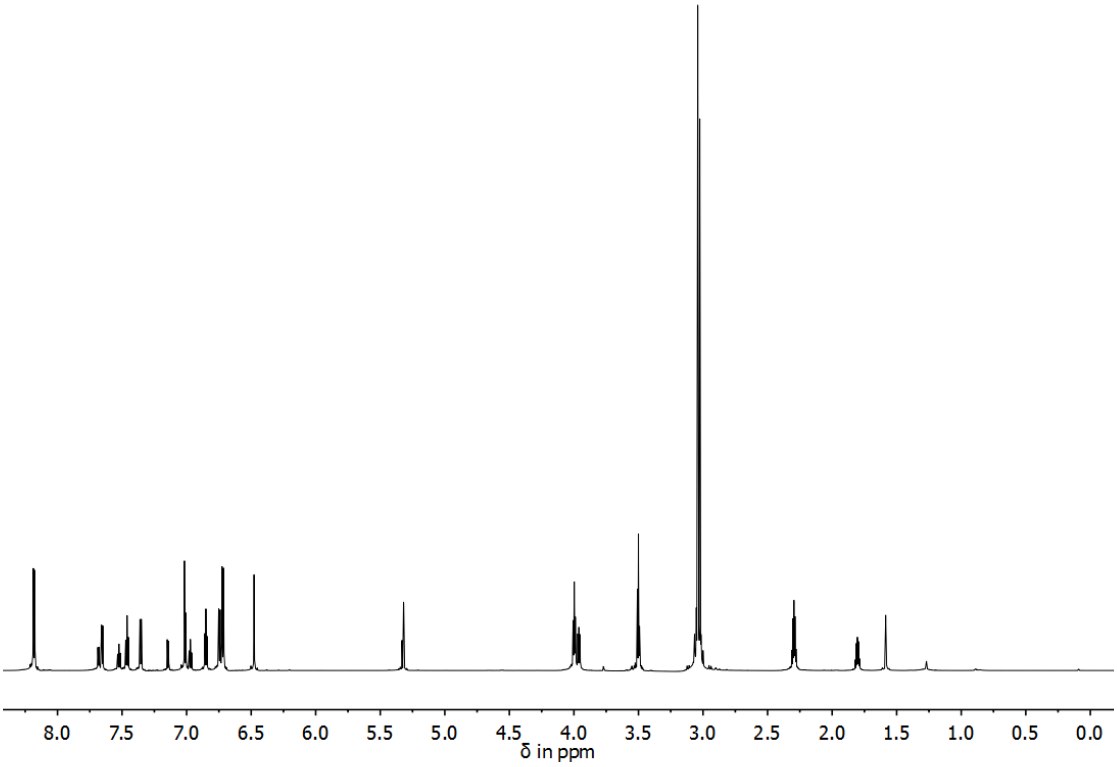

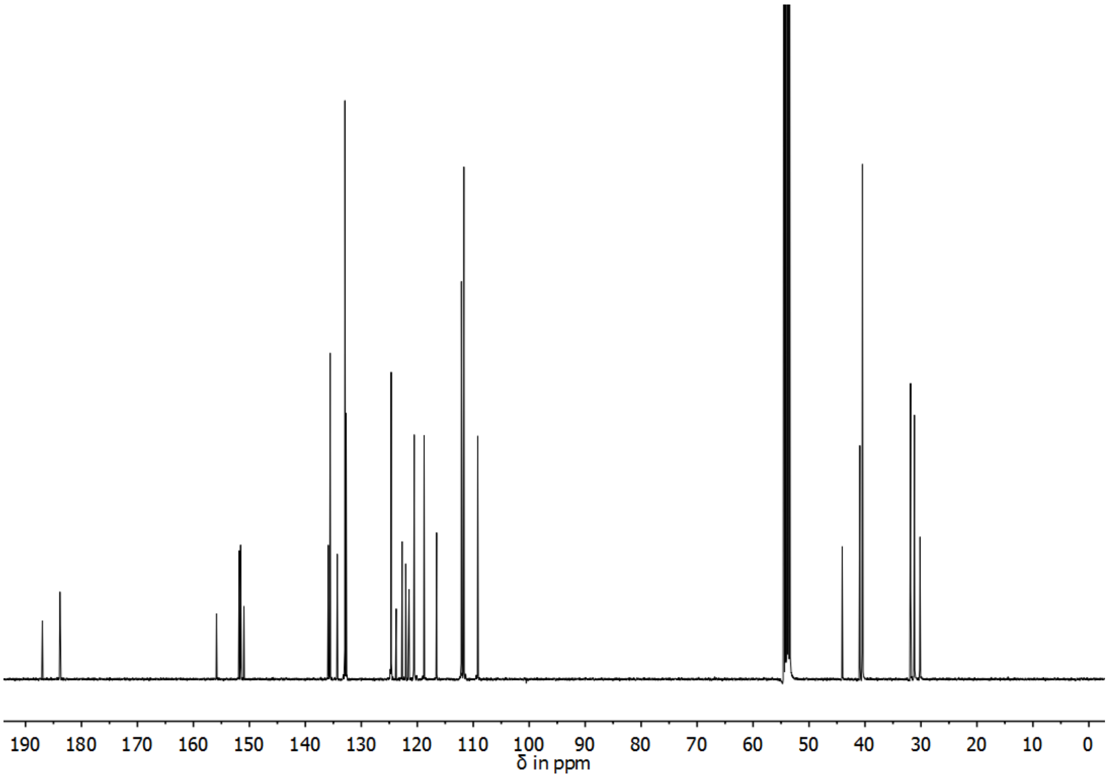

Figure S17 NMR spectra of **6** ***Z*/*E*** (37% *Z* isomer and 63% *E* isomer) (CD_2Cl_2, 400 MHz, 27 °C). Upper: ^{1}H NMR spectrum. Lower: ^{13}C NMR spectrum.

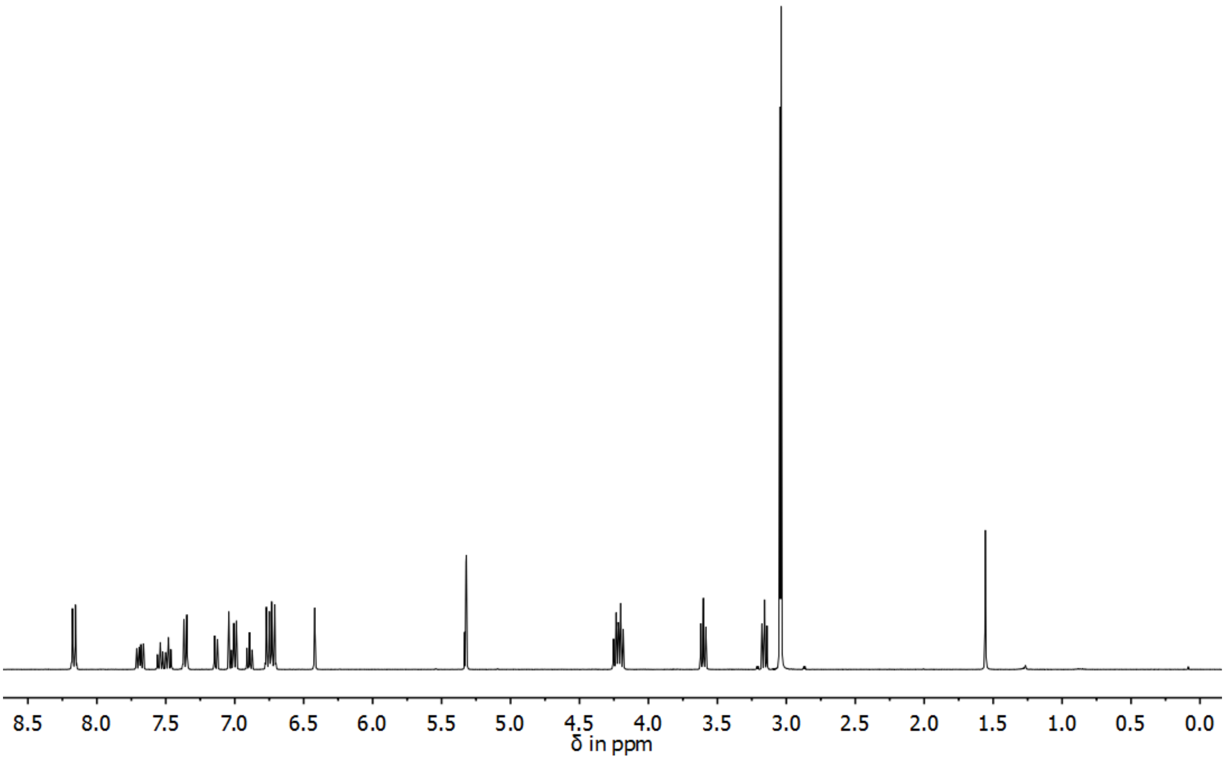

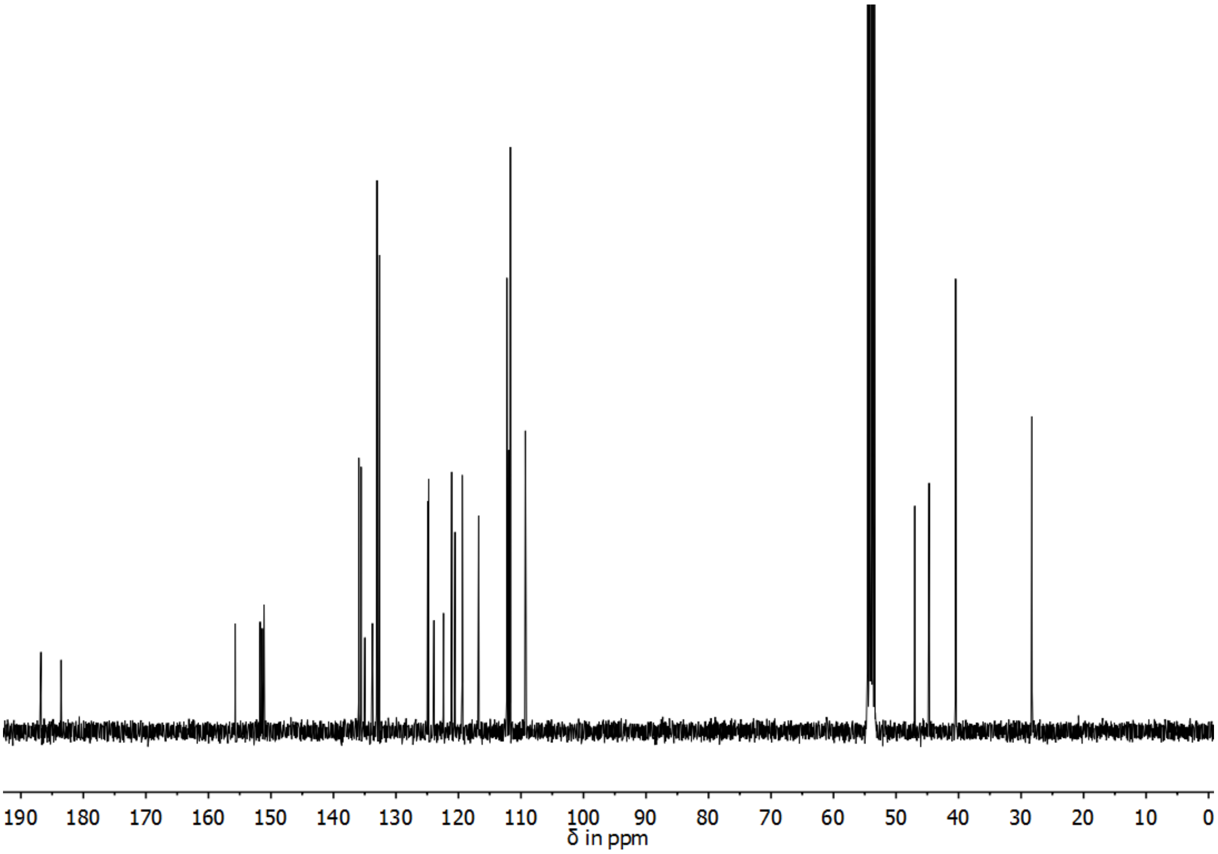

Figure S18 NMR spectra of **7** ***Z*/*E*** (43% *Z* isomer and 57% *E* isomer) (CD_2Cl_2, 400 MHz, 27 °C). Upper: ^{1}H NMR spectrum. Lower: ^{13}C NMR spectrum.

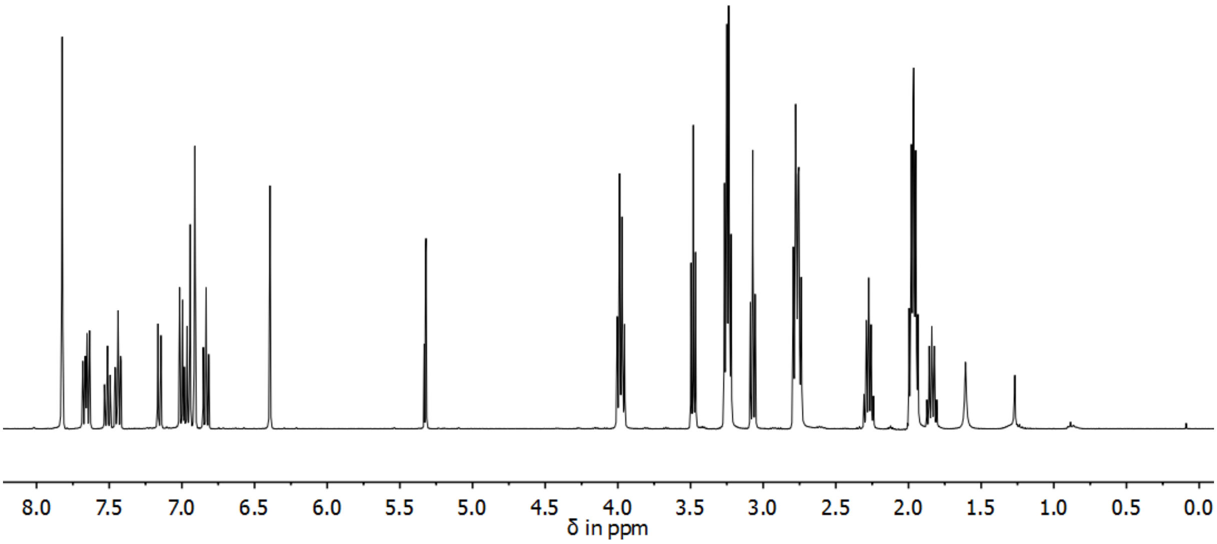

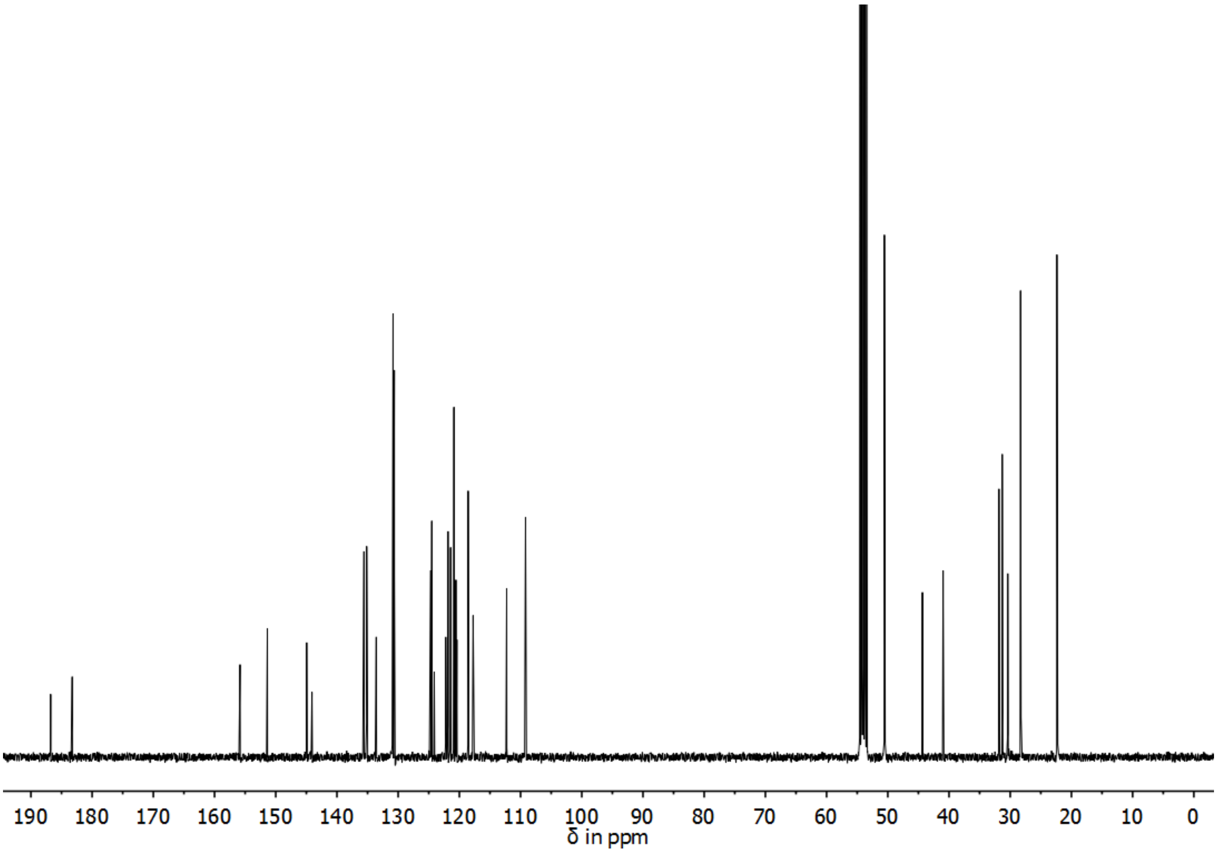

Figure S19 NMR spectra of **8 *Z*/*E*** (41% *Z* isomer and 59% *E* isomer) (CD_2Cl_2, 400 MHz, 27 °C). Upper: ^{1}H NMR spectrum. Lower: ^{13}C NMR spectrum.

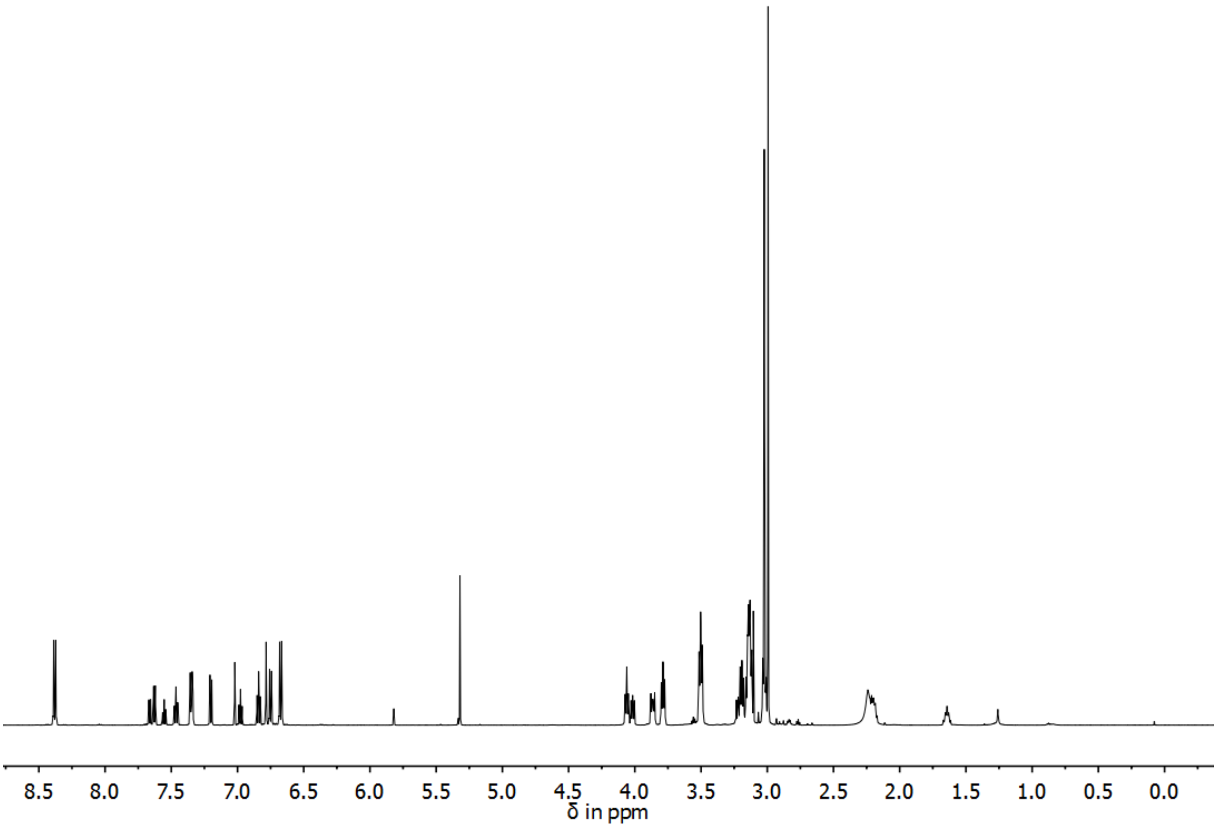

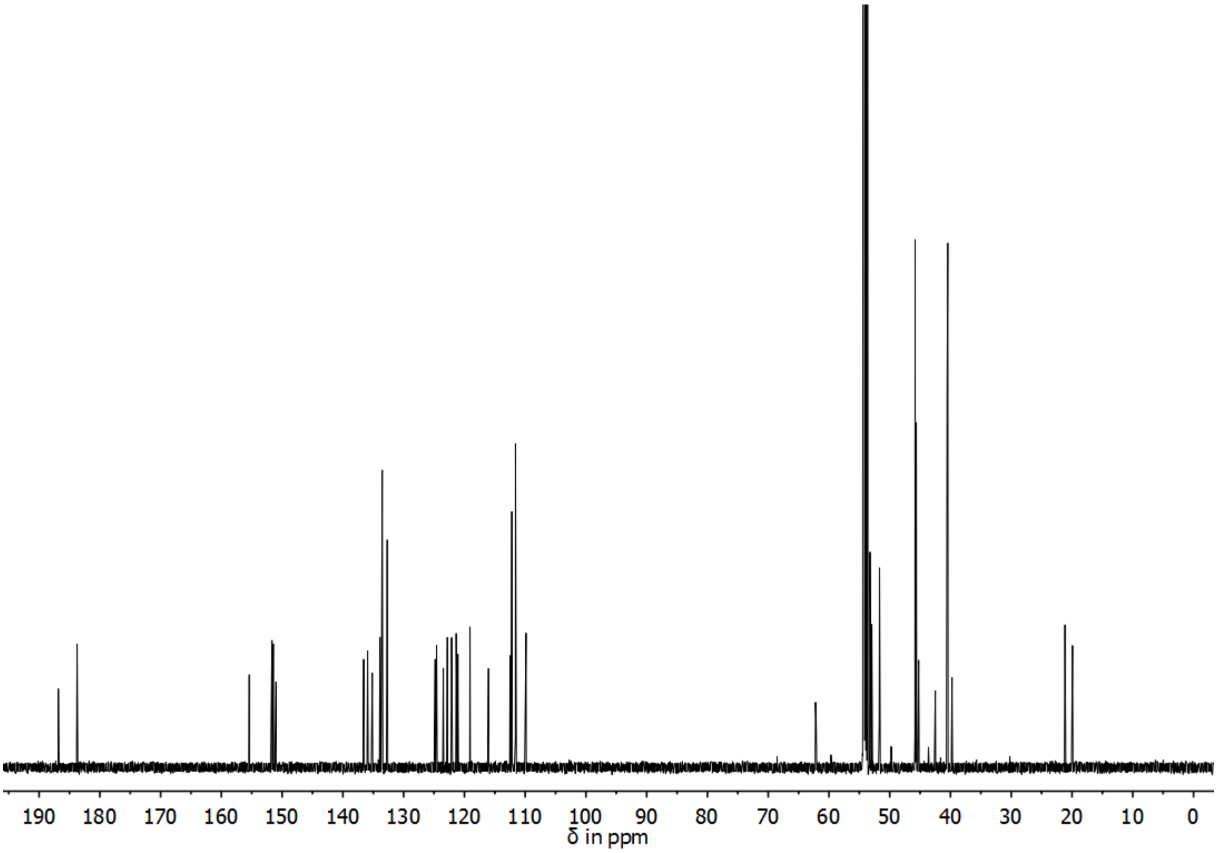

Figure S20 NMR spectra of **1** ***Z*****/*****E*** (39% *Z* isomer and 61% *E* isomer) (CD_2Cl_2, 600 MHz, 27 °C). Upper: ^{1}H NMR spectrum. Lower: ^{13}C NMR spectrum.

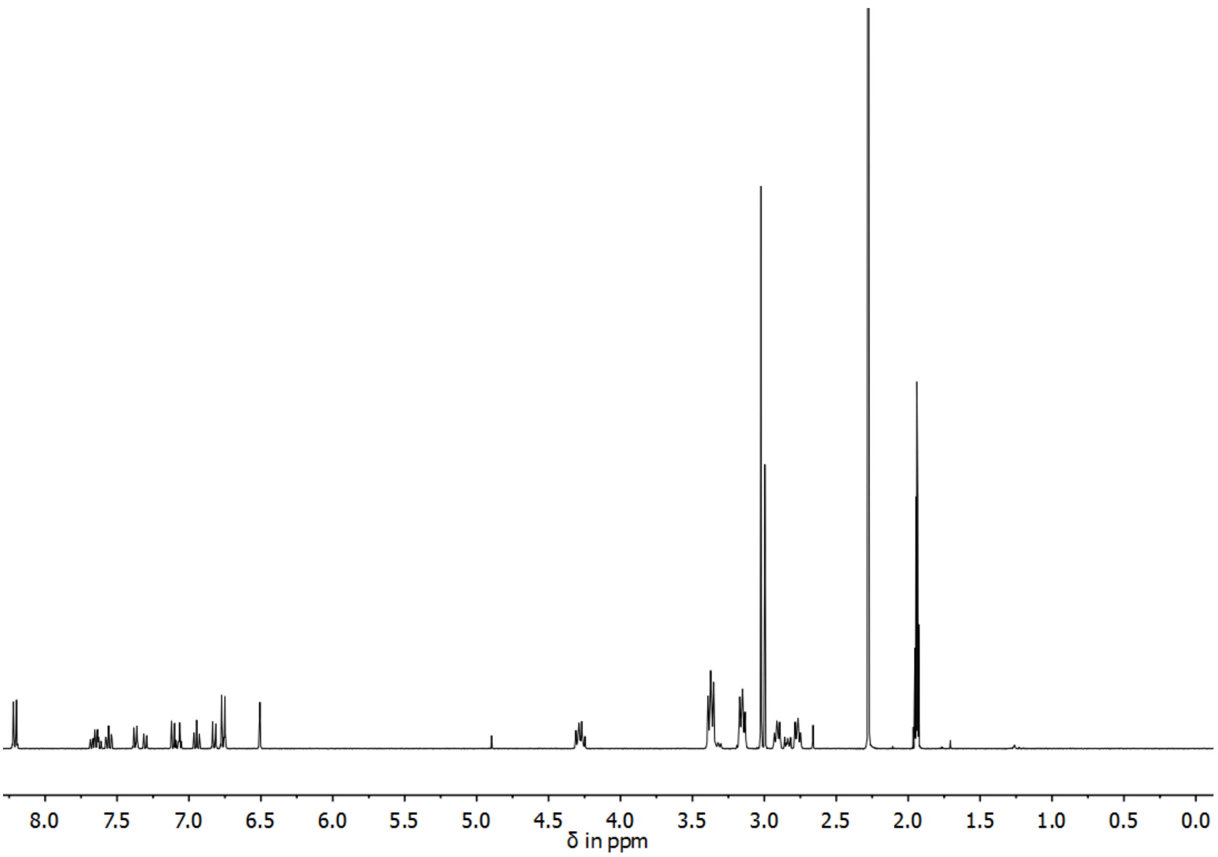

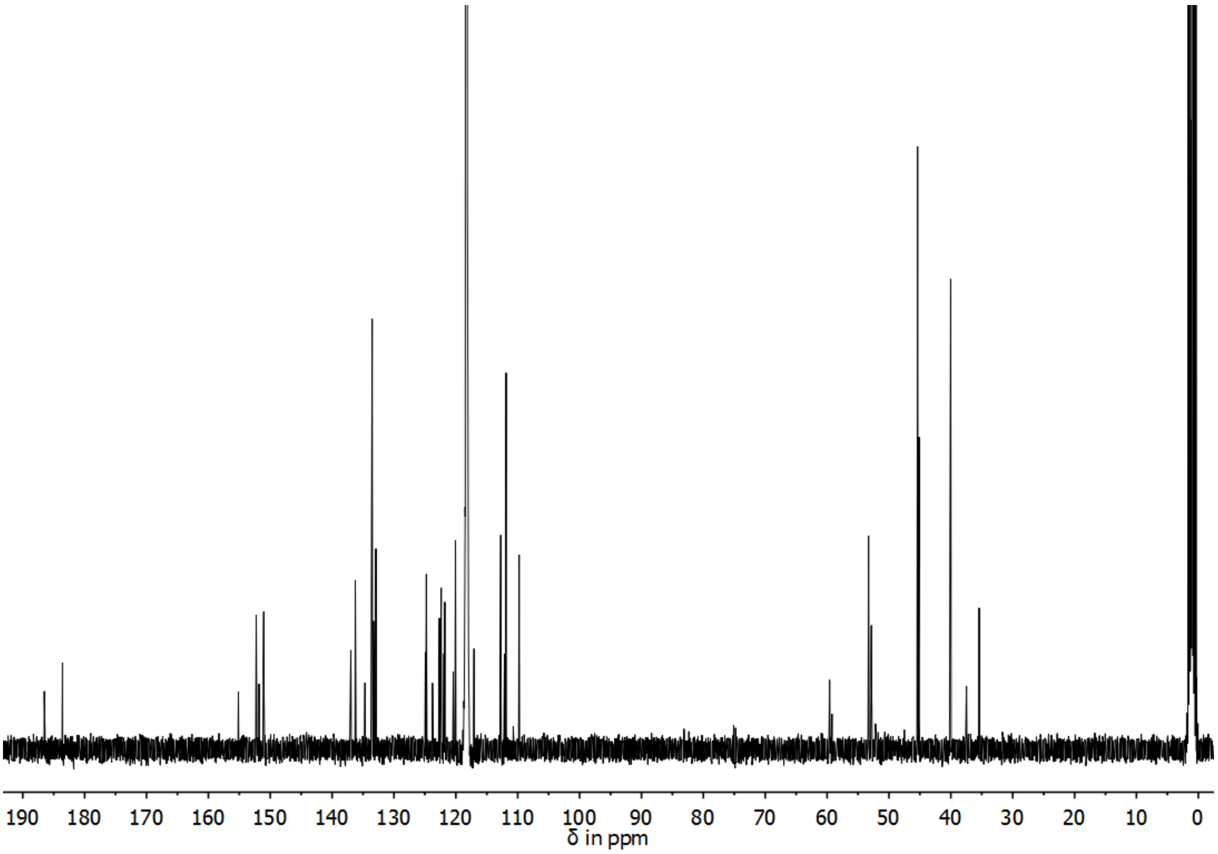

Figure S21 NMR spectra of **2** ***Z*/*E*** (34% *Z* isomer and 66% *E* isomer) (CD_3CN, 400 MHz, 27 °C). Upper: ^{1}H NMR spectrum. Lower: ^{13}C NMR spectrum.

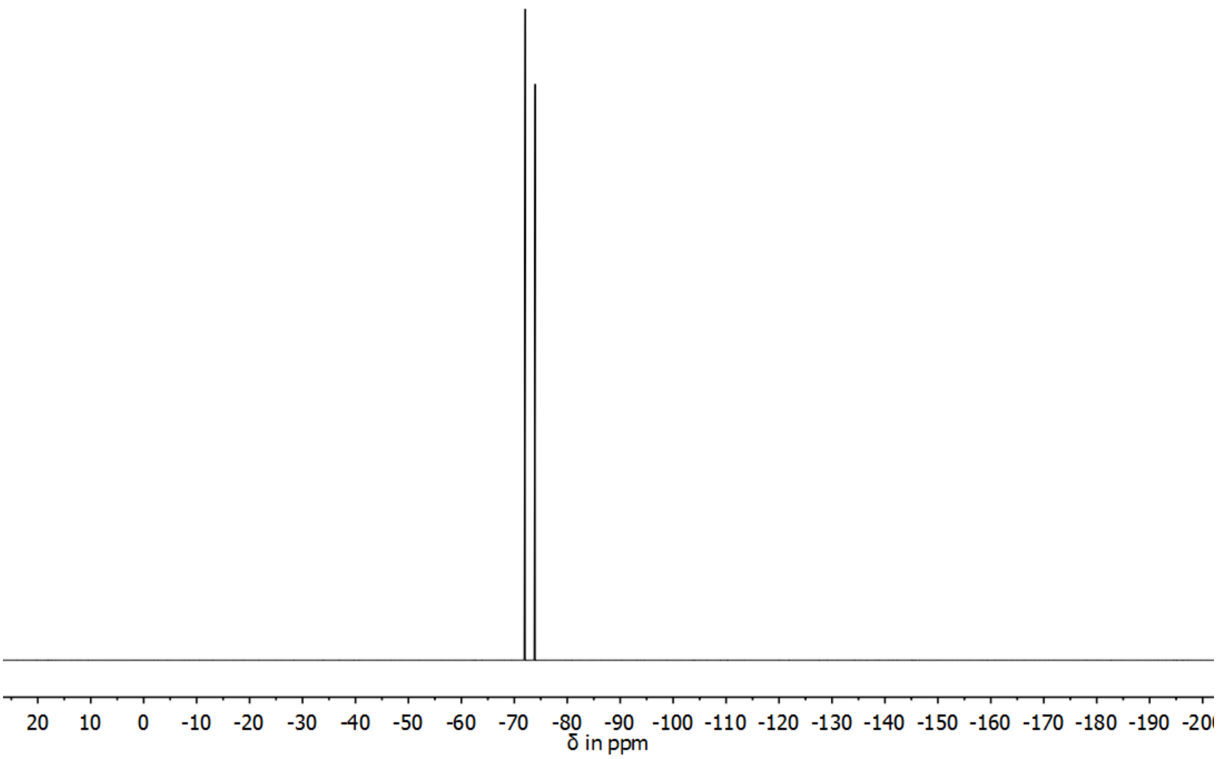

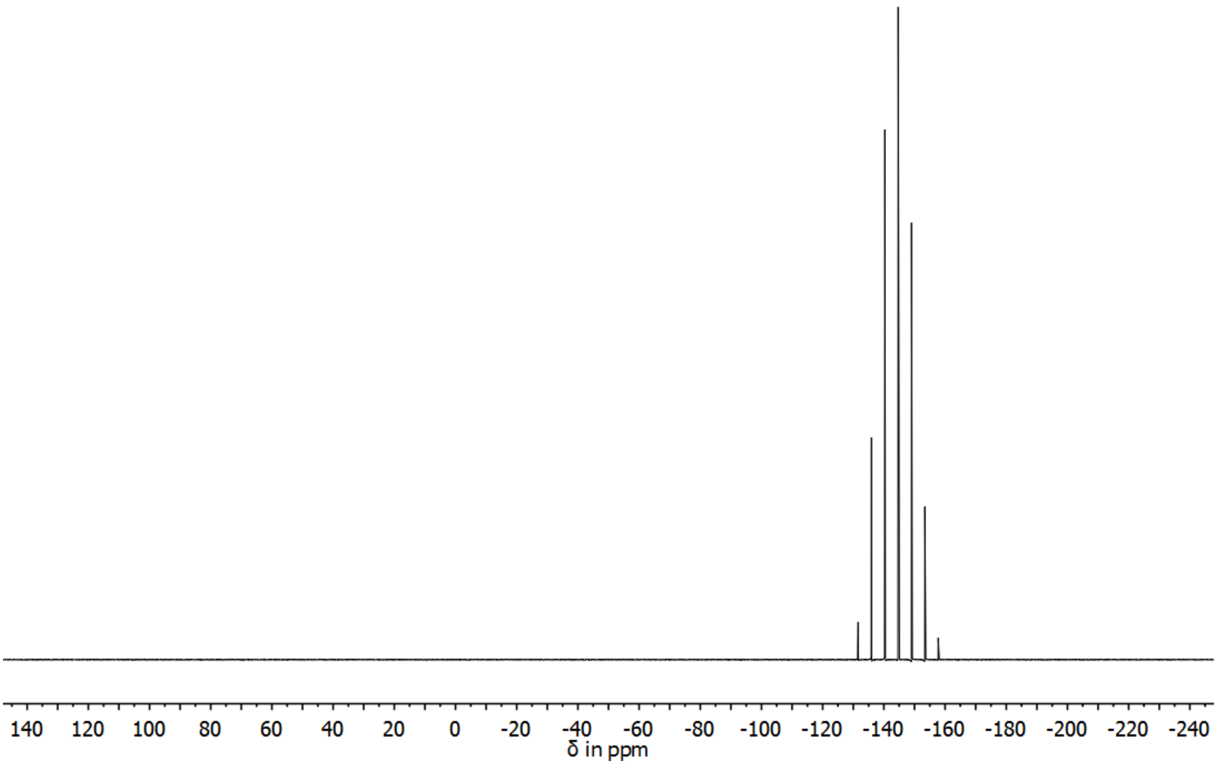

Figure S22 NMR spectra of **2** (CD_3CN, 376/162 MHz, 27 °C). Upper: ^{19}F NMR spectrum. Lower: ^{31}P NMR spectrum.

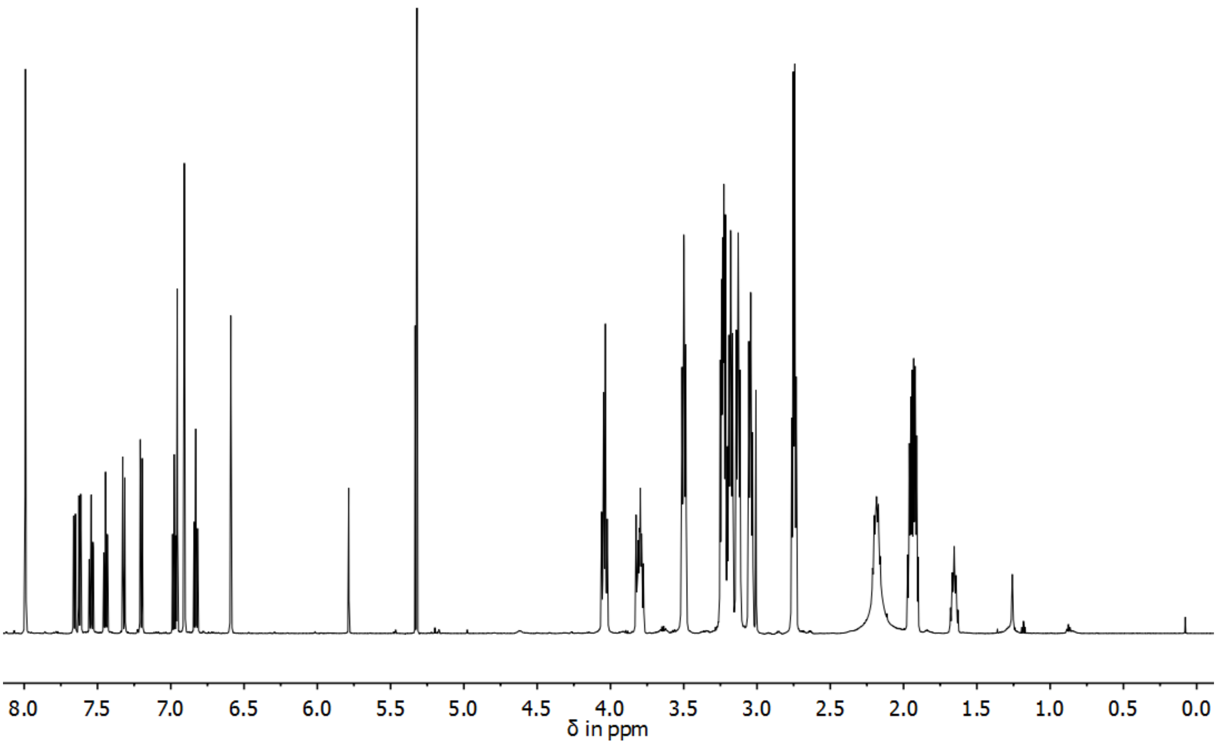

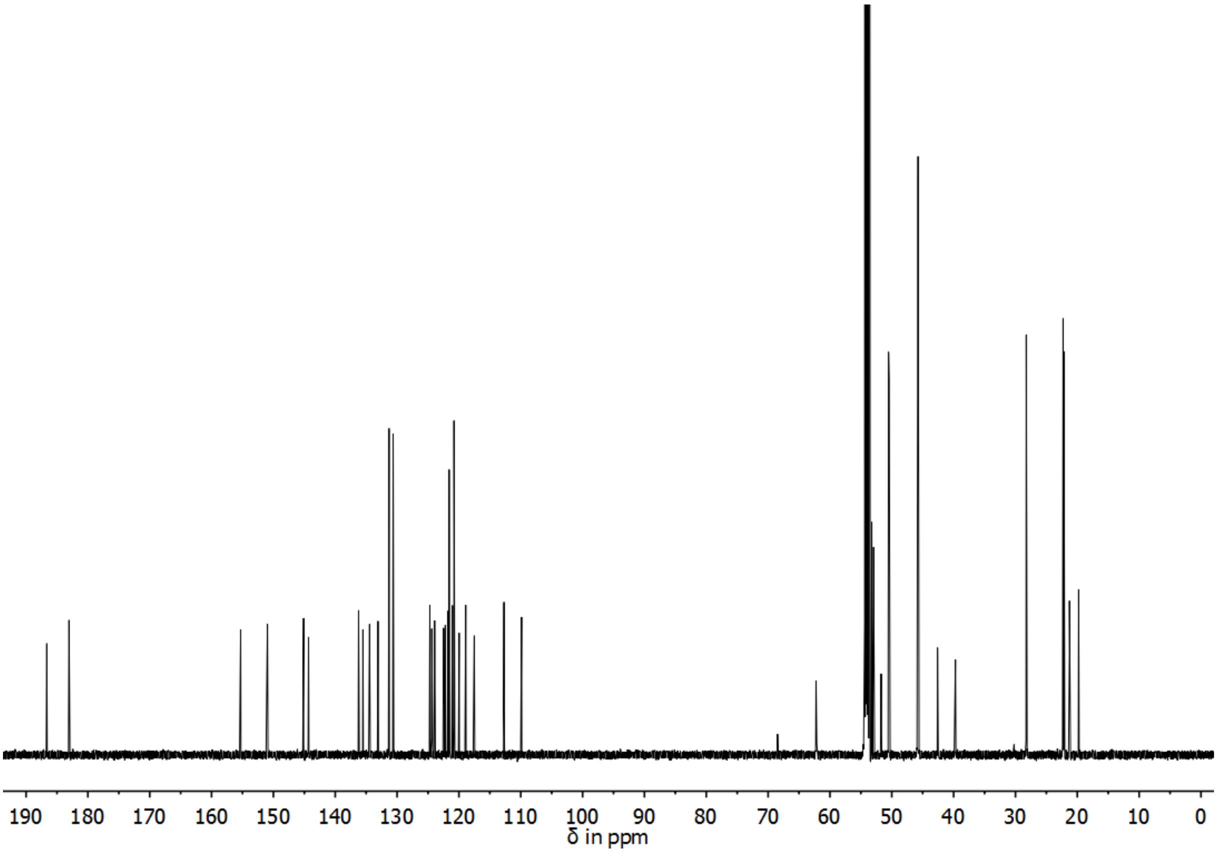

Figure S23 NMR spectra of **3** ***Z*/*E*** (45% *Z* isomer and 55% *E* isomer) (CD_2Cl_2, 600 MHz, 27 °C). Upper: ^{1}H NMR spectrum. Lower: ^{13}C NMR spectrum.

References

[1] Petermayer, C.; Thumser, S.; Kink, F.; Mayer, P.; Dube, H., Hemiindigo: Highly Bistable Photoswitching at the Biooptical Window, *J. Am. Chem. Soc.*, **2017**, *139*, 15060.

[2] Velezheva, V. S.; Brennan, P. J.; Marshakov, V. Y., Novel Pyridazino[4,3-b]indoles with Dual Inhibitory Activity Against Mycobacterium Tuberculosis and Monoamine Oxidase. *J. Med. Chem.* **2004**, *47*, 3455.

[3] B. Almarzoqi, The Quarternisation of Tertiary Amines with Dihalomethane. *Tetrahedron* **1986**, *42*, 601.

[4] B. D. Adamson; N. J. A. Coughlan; P. B. Markworth; R. E. Continetti; E. J. Bieske, An Ion Mobility Mass Spectrometer for Investigating Photoisomerization and Photodissociation of Molecular Ions. *Rev. Sci. Instrum* **2014**, *85*, 123109.

[5] Avogadro: an open-source molecular builder and visualization tool. Version 1.2.0 http://avogadro.cc/

[6] Gaussian 16, Revision B.01, M. J. Frisch, G. W. Trucks, H. B. Schlegel, G. E. Scuseria, M. A. Robb, J. R. Cheeseman, G. Scalmani, V. Barone, G. A. Petersson, H. Nakatsuji, X. Li, M. Caricato, A. V. Marenich, J. Bloino, B. G. Janesko, R. Gomperts, B. Mennucci, H. P. Hratchian, J. V. Ortiz, A. F. Izmaylov, J. L. Sonnenberg, Williams, F. Ding, F. Lipparini, F. Egidi, J. Goings, B. Peng, A. Petrone, T. Henderson, D. Ranasinghe, V. G. Zakrzewski, J. Gao, N. Rega, G. Zheng, W. Liang, M. Hada, M. Ehara, K. Toyota, R. Fukuda, J. Hasegawa, M. Ishida, T. Nakajima, Y. Honda, O. Kitao, H. Nakai, T. Vreven, K. Throssell, J. A. Montgomery Jr., J. E. Peralta, F. Ogliaro, M. J. Bearpark, J. J. Heyd, E. N. Brothers, K. N. Kudin, V. N. Staroverov, T. A. Keith, R. Kobayashi, J. Normand, K. Raghavachari, A. P. Rendell, J. C. Burant, S. S. Iyengar, J. Tomasi, M. Cossi, J. M. Millam, M. Klene, C. Adamo, R. Cammi, J. W. Ochterski, R. L. Martin, K. Morokuma, O. Farkas, J. B. Foresman, D. J. Fox, Wallingford, CT, 2016.

[7] A. A. Shvartsburg; M. F. Jarrold, An Exact Hard-Spheres Scattering Model for the Mobilities of Polyatomic Ions. *Chem. Phys. Lett.*, **1996**, *261*, 86-91

[8] MRCC, a Quantum Chemical Program Suite. Kállay, Z. Rolik, J. Csontos, P. Nagy, G. Samu, D. Mester, J. Csóka, B. Szabó, I. Ladjánszki, L. Szegedy, B. Ladóczki, K. Petrov, M. Farkas, P. D. Mezei, and B. Hégely.B., www.mrcc.hu

Appendix: Cartesian coordinates for calculated structures

Cartesian coordinates for lower energy conformations of **HI 1**, **HI 2** and **HI 3**. Structures were optimised at the DFT ωB97X-D/cc-pVDZ level of theory using the Gaussian16 package [6] and are depicted in Figures S1, S2 and S3.

E-1A

O	0.61072	1.570929	-1.962635
N	1.225247	-2.255629	0.333359
N	-0.307704	-3.923565	-0.861715
N	-5.715102	0.824359	-0.346865
N	2.130584	1.649214	1.243704
C	0.963532	-3.844263	-1.566581
C	-0.934037	-2.607414	-0.849927
C	-0.077447	-4.363846	0.506017
C	1.95228	-2.920649	-0.809711
C	0.016425	-1.548722	-0.240045
C	0.763295	-3.320244	1.290315
C	2.176052	-1.289243	0.997255
C	1.650558	-0.602555	2.258094
C	2.232072	0.819241	2.434569
C	-6.726763	0.844725	0.692046
C	-6.120352	0.72014	-1.735524
C	-4.403811	1.025278	-0.029631
C	-3.397927	1.073674	-1.029143
C	-3.985968	1.198465	1.315018
C	-2.069581	1.285992	-0.711317
C	-2.655335	1.406371	1.617479
C	-1.649815	1.455976	0.626319
C	-0.286492	1.634614	1.081438
C	0.928989	1.642966	0.468464
C	1.322426	1.685123	-0.971761
C	3.682535	1.958651	-2.017291
C	5.039905	2.003294	-1.727058
C	5.473516	1.916848	-0.393979
C	4.582911	1.791247	0.66986
C	3.217187	1.760016	0.371894
C	2.782131	1.833585	-0.958398
H	1.389792	-4.851532	-1.66434
H	0.781562	-3.462561	-2.579845
H	-1.866516	-2.663722	-0.272749
H	-1.200827	-2.320271	-1.87529
H	0.43841	-5.333219	0.483613
H	-1.043613	-4.51637	1.004767
H	2.793877	-3.471052	-0.368258
H	2.355557	-2.116375	-1.438885
H	-0.46028	-0.980806	0.563947
H	0.388719	-0.829395	-0.981412
H	1.66245	-3.752143	1.750529
H	0.182327	-2.815796	2.071716
H	3.09082	-1.861149	1.20712
H	2.422455	-0.5587	0.217735
H	0.556076	-0.524359	2.222214
H	1.892823	-1.192315	3.154602
H	3.293069	0.762168	2.709909
H	1.723945	1.295558	3.286581
H	-6.763815	1.817043	1.2126
H	-6.552085	0.056456	1.442958

H	-7.708824	0.664369	0.241627
H	-5.637566	-0.135998	-2.235385
H	-5.87929	1.63395	-2.304962
H	-7.203919	0.566878	-1.783322
H	-0.225865	1.727119	2.172481
H	-3.662635	0.965054	-2.079528
H	-4.710097	1.182975	2.127308
H	-1.329526	1.340575	-1.508411
H	-2.378651	1.545038	2.666226
H	3.308201	2.019196	-3.040411
H	5.772399	2.112687	-2.52703
H	6.542981	1.960271	-0.179702
H	4.953393	1.752698	1.694865

E-1B

O	0.965664	1.800354	-1.919043
N	0.208232	-2.081243	0.649082
N	-0.628297	-3.340945	-1.42032
N	-5.349475	0.981761	-0.333859
N	2.474539	1.342842	1.264017
C	-1.766353	-2.844517	-0.657542
C	0.163213	-4.234369	-0.586505
C	0.199667	-2.214068	-1.831287
C	-1.298637	-2.210349	0.675569
C	0.808606	-3.459628	0.593281
C	0.567282	-1.334756	-0.613053
C	0.632569	-1.348846	1.899032
C	2.132102	-1.033926	2.056603
C	2.435358	0.441724	2.407347
C	-5.766189	0.958328	-1.72296
C	-6.354011	0.9452	0.711549
C	-4.041102	1.208804	-0.016708
C	-3.046716	1.347711	-1.018566
C	-3.61448	1.325331	1.330961
C	-1.719755	1.580279	-0.699802
C	-2.285318	1.556451	1.633799
C	-1.288422	1.675772	0.640717
C	0.087178	1.759717	1.104585
C	1.293643	1.602548	0.501207
C	1.688031	1.655326	-0.943393
C	4.040254	1.420587	-2.00982
C	5.370757	1.132239	-1.737252
C	5.775148	0.877842	-0.416615
C	4.882969	0.912496	0.652689
C	3.548884	1.225127	0.37418
C	3.139419	1.455373	-0.944333
H	-2.29737	-2.097954	-1.263418
H	-2.462673	-3.670011	-0.457946
H	0.945305	-4.705757	-1.196273
H	-0.487879	-5.03499	-0.209985
H	-0.339461	-1.604715	-2.568417
H	1.102475	-2.602137	-2.321847
H	-1.705513	-1.20086	0.828357
H	-1.542339	-2.827667	1.550947
H	1.891589	-3.330153	0.471538
H	0.624306	-3.934605	1.56618
H	-0.009051	-0.404332	-0.590284
H	1.635006	-1.092142	-0.577578
H	0.032195	-0.43547	1.900578
H	0.27669	-1.976338	2.727779
H	2.541496	-1.672747	2.852701

H	2.707364	-1.287903	1.152705
H	3.415407	0.490584	2.898218
H	1.721807	0.818621	3.153942
H	-5.253403	0.160303	-2.285125
H	-5.571344	1.9185	-2.230884
H	-6.842435	0.760341	-1.772135
H	-6.410564	1.898173	1.265439
H	-6.156143	0.136358	1.434376
H	-7.334724	0.755943	0.261818
H	0.145269	1.807241	2.198191
H	-3.31929	1.305339	-2.071898
H	-4.330557	1.254687	2.147687
H	-0.99006	1.707083	-1.4987
H	-2.003745	1.646114	2.686871
H	3.686966	1.615369	-3.023511
H	6.105791	1.104138	-2.541844
H	6.824274	0.652288	-0.216742
H	5.235718	0.724578	1.667062

E-1C

O	1.054423	2.256521	-1.487272
N	0.589935	-2.278684	-0.329176
N	-1.835884	-3.050642	-0.669082
N	-5.338433	1.421121	-0.155577
N	2.520665	0.702143	1.349318
C	-1.684044	-2.478639	0.660204
C	-0.935896	-4.18557	-0.809716
C	-1.507065	-2.034373	-1.66055
C	-0.199773	-2.134898	0.945477
C	0.546387	-3.730635	-0.727523
C	-0.088508	-1.468393	-1.412481
C	2.036376	-1.8092	-0.225631
C	2.73129	-1.808469	1.135322
C	2.51481	-0.574768	2.047243
C	-6.352159	1.160035	0.848059
C	-5.739267	1.80409	-1.49576
C	-4.017739	1.39072	0.181664
C	-3.002336	1.688191	-0.764082
C	-3.595999	1.066111	1.496908
C	-1.661795	1.665653	-0.421895
C	-2.252754	1.042767	1.820984
C	-1.239074	1.328613	0.881131
C	0.133866	1.152182	1.321432
C	1.342901	1.190648	0.700961
C	1.764063	1.80801	-0.600144
C	4.163367	2.20087	-1.515805
C	5.508214	1.939104	-1.285486
C	5.898218	1.220373	-0.144181
C	4.974328	0.756925	0.790582
C	3.626054	1.042181	0.561959
C	3.231053	1.741837	-0.584966
H	-2.297826	-1.56943	0.72824
H	-2.053343	-3.191309	1.410032
H	-1.119072	-4.68025	-1.772912
H	-1.160462	-4.913312	-0.018303
H	-2.239401	-1.217506	-1.59771
H	-1.580587	-2.479168	-2.662125
H	-0.083204	-1.103034	1.278461
H	0.257213	-2.810935	1.678382
H	1.060077	-3.80552	-1.695089
H	1.128771	-4.288307	0.018363

H	-0.106303	-0.430132	-1.059088
H	0.558134	-1.524315	-2.298134
H	2.588536	-2.457595	-0.918028
H	2.06294	-0.800762	-0.650166
H	2.540043	-2.733004	1.702948
H	3.801116	-1.845009	0.878152
H	3.314325	-0.571011	2.8025
H	1.582864	-0.65461	2.618709
H	-6.309351	1.889133	1.674982
H	-6.251215	0.147295	1.273075
H	-7.342745	1.231343	0.386203
H	-5.330022	1.115813	-2.254275
H	-5.413496	2.827959	-1.745678
H	-6.831647	1.769668	-1.567993
H	0.190515	0.827484	2.367722
H	-3.266362	1.971509	-1.781622
H	-4.324996	0.845758	2.274479
H	-0.913603	1.93187	-1.168132
H	-1.973441	0.790841	2.848048
H	3.824875	2.74826	-2.396696
H	6.265694	2.290781	-1.98615
H	6.958742	1.023179	0.022673
H	5.306763	0.214402	1.676693

E-1D

O	-0.390568	-1.998543	-1.760785
N	2.030714	2.02897	0.483857
N	2.885923	3.076544	-1.690127
N	-6.37821	0.328054	-0.3941
N	1.281158	-1.600784	1.329023
C	1.601861	3.66725	-1.344246
C	2.725147	1.640518	-1.874574
C	3.840777	3.326034	-0.621391
C	1.025399	3.016122	-0.059407
C	2.240779	0.965723	-0.566274
C	3.325933	2.75491	0.727941
C	1.473852	1.447787	1.761909
C	2.322587	0.367714	2.451278
C	1.598753	-0.982802	2.593032
C	-7.294794	0.836496	0.606912
C	-6.860758	0.057493	-1.734417
C	-5.094383	0.016471	-0.052331
C	-4.609298	0.221207	1.264536
C	-4.18772	-0.528674	-0.996432
C	-3.309584	-0.107159	1.59248
C	-2.889433	-0.853018	-0.653102
C	-2.402956	-0.65305	0.656687
C	-1.071271	-0.955052	1.142121
C	0.06654	-1.458213	0.588002
C	0.350999	-1.976962	-0.79059
C	2.551812	-2.955642	-1.760205
C	3.885799	-3.227046	-1.473421
C	4.393903	-2.957115	-0.193733
C	3.604263	-2.415965	0.820874
C	2.261185	-2.153368	0.524006
C	1.751917	-2.416725	-0.755218
H	1.734301	4.747726	-1.19971
H	0.904867	3.528736	-2.180981
H	3.683828	1.208372	-2.189364
H	2.003249	1.462717	-2.682277
H	4.00922	4.407452	-0.532234

H	4.799981	2.863878	-0.889426
H	0.830053	3.745524	0.737551
H	0.103351	2.448955	-0.244392
H	2.967472	0.246751	-0.172795
H	1.277538	0.454698	-0.682327
H	3.112006	3.537252	1.468834
H	4.025049	2.038673	1.175172
H	1.318023	2.310127	2.423845
H	0.486887	1.056783	1.485091
H	3.266033	0.19447	1.912165
H	2.614539	0.720363	3.451506
H	0.682687	-0.868419	3.187114
H	2.237834	-1.666747	3.171231
H	-6.938726	1.787102	1.038144
H	-7.445096	0.117883	1.430949
H	-8.268357	1.024939	0.141375
H	-6.810554	-1.016845	-1.980906
H	-6.284777	0.613089	-2.493029
H	-7.906835	0.373784	-1.81052
H	-0.97512	-0.705337	2.205141
H	-5.257512	0.628757	2.037995
H	-4.508387	-0.714518	-2.019981
H	-2.981493	0.055405	2.623194
H	-2.228921	-1.280805	-1.404233
H	2.121739	-3.152318	-2.743558
H	4.537947	-3.65568	-2.234533
H	5.440357	-3.182446	0.020887
H	4.028427	-2.233086	1.80915

E-1E

O	2.006259	2.963857	-1.119174
N	-3.437087	-1.879711	0.304233
N	-4.490487	-3.602656	-1.269242
N	6.212588	-2.0212	-0.304179
N	-0.55795	2.136119	1.17585
C	-4.830063	-3.934671	0.106721
C	-3.042548	-3.618736	-1.425321
C	-4.999612	-2.275703	-1.585528
C	-4.077695	-3.000876	1.087815
C	-2.390085	-2.474995	-0.605337
C	-4.500568	-1.234596	-0.548742
C	-2.836515	-0.892721	1.27749
C	-2.00285	0.214219	0.66116
C	-1.438982	1.153109	1.744904
C	7.144769	-1.471262	-1.267824
C	6.574619	-3.206013	0.444725
C	5.053323	-1.354752	-0.014887
C	4.150722	-1.832918	0.966767
C	4.704201	-0.1542	-0.680375
C	2.985907	-1.142891	1.244862
C	3.535774	0.52533	-0.389697
C	2.632821	0.051611	0.583671
C	1.381537	0.675493	0.97757
C	0.700142	1.786184	0.59099
C	1.014888	2.850053	-0.41977
C	-0.409369	4.892456	-1.153954
C	-1.626443	5.541623	-0.972819
C	-2.556011	5.037575	-0.052422
C	-2.302189	3.89582	0.707811
C	-1.070786	3.259634	0.528095
C	-0.149538	3.749038	-0.403779

H	-5.916467	-3.839246	0.235814
H	-4.565601	-4.980952	0.30895
H	-2.786362	-3.50609	-2.486938
H	-2.663987	-4.596349	-1.098209
H	-6.097366	-2.296171	-1.595104
H	-4.667321	-2.00283	-2.596052
H	-4.73828	-2.537963	1.832536
H	-3.262963	-3.511036	1.618986
H	-2.025815	-1.66324	-1.245364
H	-1.560326	-2.814103	0.029359
H	-5.291141	-0.903954	0.138487
H	-4.057786	-0.350231	-1.020458
H	-3.683972	-0.483153	1.847564
H	-2.227885	-1.49297	1.97039
H	-1.146262	-0.200086	0.109181
H	-2.592383	0.824906	-0.039858
H	-0.905731	0.577495	2.516936
H	-2.252926	1.673254	2.269701
H	7.52335	-0.481148	-0.95969
H	6.683991	-1.368015	-2.264241
H	8.002096	-2.146706	-1.364878
H	5.823288	-4.005666	0.329289
H	6.69078	-2.996411	1.522728
H	7.529912	-3.591218	0.071076
H	0.887872	0.103281	1.771977
H	4.366389	-2.742824	1.523983
H	5.364802	0.266106	-1.436688
H	2.323378	-1.541101	2.019411
H	3.310087	1.450448	-0.915966
H	0.335624	5.250266	-1.866126
H	-1.86203	6.441218	-1.541766
H	-3.50732	5.556283	0.081178
H	-3.041841	3.542635	1.427803

Z-1A

O	-4.109182	-2.246952	-1.899992
N	1.332926	2.348585	-0.261529
N	3.876101	2.025666	-0.313949
N	4.075191	-2.121841	0.73613
N	-2.524041	-0.307503	0.586429
C	3.286282	1.519888	-1.546107
C	3.309609	1.308146	0.822745
C	3.569998	3.442371	-0.184555
C	1.774392	1.856696	-1.616415
C	1.761231	1.323966	0.75904
C	2.050875	3.645481	0.029287
C	-0.153276	2.624138	-0.224606
C	-1.104079	1.44821	-0.410136
C	-1.451308	0.632149	0.843799
C	4.380312	-2.516146	2.100811
C	5.11194	-2.315846	-0.262177
C	2.752138	-2.114365	0.327863
C	2.403119	-2.010047	-1.03754
C	1.692368	-2.178545	1.259722
C	1.072539	-1.93647	-1.433179
C	0.367979	-2.094853	0.846441
C	0.020435	-1.947513	-0.50528
C	-1.376445	-1.919692	-0.958796
C	-2.432981	-1.261806	-0.448165
C	-3.820415	-1.47923	-1.009327
C	-6.050197	-0.331538	-0.316413

C	-6.605768	0.627899	0.524616
C	-5.787279	1.328369	1.419139
C	-4.41337	1.094986	1.504921
C	-3.866472	0.126956	0.663916
C	-4.680709	-0.567188	-0.238823
H	3.806719	1.954275	-2.410112
H	3.425133	0.431453	-1.57732
H	3.666358	1.784666	1.74623
H	3.66843	0.269855	0.821488
H	3.907328	3.962384	-1.091374
H	4.125471	3.865285	0.663088
H	1.543127	2.657563	-2.331988
H	1.172853	0.973118	-1.859913
H	1.293928	1.596362	1.714767
H	1.357552	0.360768	0.425344
H	1.624636	4.409703	-0.633417
H	1.800526	3.909381	1.065655
H	-0.336584	3.130888	0.735044
H	-0.317848	3.361372	-1.022482
H	-2.042866	1.888592	-0.783434
H	-0.759852	0.775127	-1.209056
H	-1.775078	1.304878	1.650439
H	-0.581071	0.084207	1.223531
H	4.030977	-3.537449	2.337874
H	3.927164	-1.819392	2.823019
H	5.465527	-2.478338	2.249436
H	5.110145	-1.495744	-0.997417
H	5.008253	-3.271805	-0.807159
H	6.090246	-2.303655	0.23161
H	-1.63368	-2.532463	-1.829772
H	3.173326	-2.026988	-1.807453
H	1.895463	-2.321797	2.31965
H	0.842369	-1.889239	-2.500797
H	-0.425005	-2.184584	1.592542
H	-6.655396	-0.892783	-1.029644
H	-7.675392	0.835797	0.493108
H	-6.234278	2.076289	2.076576
H	-3.811764	1.647447	2.227605

Z-1B

O	4.425445	2.112086	1.816668
N	-1.887652	-1.765259	-0.192236
N	-3.82537	-2.909899	1.02558
N	-3.830791	2.179065	-0.576761
N	2.702695	0.235229	-0.596934
C	-3.178829	-1.952605	1.914564
C	-2.852093	-3.907566	0.601277
C	-4.334701	-2.204907	-0.143619
C	-1.896311	-1.378996	1.26427
C	-1.755754	-3.261241	-0.286474
C	-3.21277	-1.364208	-0.799641
C	-0.792341	-1.063184	-0.952122
C	0.587408	-1.051507	-0.313296
C	1.547833	-0.303513	-1.260305
C	-4.250085	2.619401	-1.897997
C	-4.808003	2.339171	0.488114
C	-2.481426	2.296513	-0.247673
C	-2.039172	2.179618	1.088145
C	-1.492258	2.499654	-1.231979
C	-0.6832	2.149189	1.393165
C	-0.137721	2.466339	-0.906858

C	0.304476	2.228391	0.400628
C	1.723522	2.106214	0.782929
C	2.68575	1.271793	0.3441
C	4.068437	1.322098	0.972537
C	6.111623	-0.230069	0.54338
C	6.550771	-1.336058	-0.179822
C	5.682677	-1.969678	-1.076118
C	4.373094	-1.528357	-1.281146
C	3.946486	-0.41493	-0.55962
C	4.809375	0.216445	0.346449
H	-3.892133	-1.147554	2.139111
H	-2.929059	-2.44375	2.864482
H	-3.361977	-4.704327	0.043579
H	-2.40686	-4.365605	1.494782
H	-5.163339	-1.54952	0.156458
H	-4.738439	-2.942444	-0.850478
H	-1.838019	-0.283935	1.306723
H	-0.984387	-1.797716	1.706808
H	-1.865604	-3.518305	-1.348732
H	-0.739943	-3.525536	0.031703
H	-3.329985	-0.283669	-0.626694
H	-3.13026	-1.533003	-1.881667
H	-0.768007	-1.537785	-1.945032
H	-1.13718	-0.027419	-1.078068
H	0.555837	-0.502721	0.638034
H	0.973905	-2.062433	-0.11097
H	1.005868	0.516557	-1.751213
H	1.887799	-0.970045	-2.064139
H	-3.752439	2.03004	-2.682521
H	-4.039287	3.689022	-2.081577
H	-5.329406	2.456473	-2.002755
H	-4.735391	3.318096	0.996594
H	-4.696406	1.54996	1.248034
H	-5.815238	2.242996	0.065536
H	2.072317	2.744759	1.602639
H	-2.754401	2.119666	1.907612
H	-1.769403	2.698411	-2.265956
H	-0.380184	2.046489	2.438043
H	0.596003	2.654789	-1.693432
H	6.757514	0.286282	1.254801
H	7.565928	-1.711555	-0.051237
H	6.036278	-2.8367	-1.637152
H	3.729418	-2.048198	-1.991517

Z-1C

O	4.23365	2.335899	1.704572
N	-1.448276	-1.727605	0.166656
N	-3.501877	-3.168826	0.67394
N	-4.008216	1.931491	-0.468582
N	2.481567	0.30252	-0.566127
C	-3.302656	-2.205155	1.749501
C	-2.320289	-4.014264	0.562787
C	-3.700797	-2.452357	-0.579623
C	-1.970469	-1.442794	1.554128
C	-1.096735	-3.187583	0.086339
C	-2.559545	-1.431818	-0.811144
C	-0.263146	-0.831341	-0.087649
C	0.583315	-1.170041	-1.304152
C	1.551713	-0.018873	-1.624641
C	-4.946609	1.984815	0.639456
C	-4.538832	2.27749	-1.777614

C	-2.669913	2.210151	-0.208722
C	-2.155984	2.196661	1.107044
C	-1.755783	2.482815	-1.248113
C	-0.789952	2.307088	1.341766
C	-0.392676	2.5997	-0.99141
C	0.133699	2.43466	0.295076
C	1.573245	2.356366	0.612549
C	2.496807	1.443723	0.256783
C	3.869155	1.49761	0.912
C	5.878319	-0.133317	0.633509
C	6.309524	-1.280828	-0.028217
C	5.448957	-1.939207	-0.913479
C	4.149104	-1.488362	-1.158762
C	3.726576	-0.341625	-0.48776
C	4.58724	0.323742	0.393729
H	-4.151925	-1.508124	1.755995
H	-3.298838	-2.727817	2.715313
H	-2.517937	-4.829376	-0.145956
H	-2.122175	-4.471615	1.541523
H	-4.665039	-1.927041	-0.553427
H	-3.747237	-3.184611	-1.397252
H	-2.074587	-0.353042	1.633408
H	-1.1874	-1.770317	2.251129
H	-0.834692	-3.396843	-0.957893
H	-0.203419	-3.342754	0.706274
H	-2.877844	-0.396487	-0.622538
H	-2.137892	-1.488983	-1.821542
H	-0.68399	0.175587	-0.178441
H	0.352317	-0.856222	0.822506
H	1.15368	-2.096837	-1.138549
H	-0.042076	-1.32265	-2.198307
H	0.977495	0.877994	-1.874709
H	2.124121	-0.291145	-2.525329
H	-4.982243	2.975682	1.12858
H	-4.694543	1.235129	1.405886
H	-5.950777	1.746416	0.269162
H	-4.013677	1.725355	-2.5713
H	-4.464299	3.357941	-1.999328
H	-5.595299	1.987243	-1.82261
H	1.963526	3.067781	1.349735
H	-2.822868	2.103994	1.963455
H	-2.101927	2.617431	-2.271366
H	-0.425435	2.261223	2.370684
H	0.278477	2.831761	-1.820858
H	6.525661	0.411777	1.321788
H	7.315778	-1.666238	0.136212
H	5.80032	-2.833195	-1.431955
H	3.507563	-2.025806	-1.858345

E-2A

O	-0.018313	2.513519	1.338534
N	2.155075	-2.048977	-0.31304
N	1.017897	-3.446661	1.510527
N	-6.090797	0.273231	0.234161
N	1.795878	1.023565	-1.314004
C	2.883207	-1.271806	-1.382223
C	2.144236	-0.129658	-2.100236
C	0.186022	-3.427799	0.31535
C	1.211876	-2.082429	1.985265
C	2.311241	-4.030494	1.187194
C	0.769718	-2.457926	-0.744525

C	2.052616	-1.264059	0.975257
C	2.947126	-3.302947	-0.024319
C	-7.011622	-0.353396	-0.69407
C	-6.597076	0.844581	1.467754
C	-4.770975	0.391687	-0.085209
C	-4.255777	-0.123296	-1.302959
C	-3.853049	1.036623	0.783712
C	-2.917972	0.01298	-1.611917
C	-2.517336	1.16739	0.459855
C	-2.001454	0.658743	-0.752148
C	-0.624189	0.720144	-1.192687
C	0.529507	1.220528	-0.670502
C	0.77789	2.122362	0.501454
C	2.989176	3.251088	1.26608
C	4.351581	3.350133	1.00543
C	4.912793	2.6442	-0.068851
C	4.144812	1.832137	-0.903133
C	2.77448	1.747782	-0.638082
C	2.212387	2.442129	0.439203
H	3.180874	-2.010054	-2.140909
H	3.795193	-0.890882	-0.901947
H	1.25608	-0.507025	-2.61988
H	2.833623	0.17686	-2.90411
H	0.119307	-4.449083	-0.083577
H	-0.830354	-3.108037	0.580589
H	1.712927	-2.100055	2.962164
H	0.228008	-1.615096	2.126635
H	2.187762	-5.09725	0.957409
H	2.962076	-3.956837	2.068708
H	0.859493	-2.9119	-1.74105
H	0.178903	-1.538418	-0.818956
H	3.083002	-1.086001	1.310632
H	1.587867	-0.304096	0.742428
H	2.918378	-3.906659	-0.94151
H	3.985485	-2.995558	0.156069
H	-6.736123	-1.402388	-0.89431
H	-7.053179	0.183946	-1.65684
H	-8.017866	-0.34877	-0.261363
H	-6.111738	0.397805	2.351252
H	-6.447618	1.936853	1.505504
H	-7.672133	0.647393	1.540197
H	-0.494716	0.226596	-2.164006
H	-4.909689	-0.623294	-2.014843
H	-4.194099	1.456597	1.72817
H	-2.565045	-0.388852	-2.56617
H	-1.849563	1.687831	1.143585
H	2.519661	3.785166	2.093422
H	4.987374	3.979084	1.628659
H	5.98229	2.737428	-0.266235
H	4.608636	1.316243	-1.745651

E-2B

O	0.059964	2.497289	1.338101
N	-2.188451	-2.010868	-0.347045
N	-1.168091	-3.387356	1.561683
N	6.119242	0.208824	0.248279
N	-1.755358	1.037648	-1.328865
C	-2.858442	-1.249078	-1.464662
C	-2.100017	-0.095882	-2.143098
C	-1.583068	-2.08556	2.067433
C	-0.185919	-3.208932	0.501558

C	-2.329874	-4.084004	1.032385
C	-2.069603	-1.177577	0.91098
C	-0.814181	-2.497616	-0.726561
C	-3.04692	-3.214613	-0.031354
C	6.627768	0.777559	1.482132
C	7.035491	-0.433417	-0.673777
C	4.80218	0.342443	-0.076554
C	3.888818	1.001042	0.786854
C	4.285459	-0.169721	-1.294791
C	2.555636	1.14556	0.458304
C	2.950251	-0.019833	-1.608344
C	2.037802	0.637933	-0.753282
C	0.66275	0.711704	-1.198841
C	-0.488794	1.22359	-0.682897
C	-0.73655	2.115116	0.497286
C	-2.948059	3.231064	1.278871
C	-4.311141	3.331831	1.021193
C	-4.872164	2.640995	-0.062802
C	-4.103752	1.841475	-0.909117
C	-2.73333	1.753625	-0.645286
C	-2.171164	2.434447	0.440515
H	-3.806249	-0.887223	-1.043407
H	-3.094257	-1.992826	-2.239201
H	-2.773666	0.229225	-2.953488
H	-1.206115	-0.469187	-2.655354
H	-2.37809	-2.234711	2.810322
H	-0.738727	-1.605405	2.579152
H	0.212954	-4.187905	0.20453
H	0.650912	-2.613639	0.891069
H	-3.021323	-4.31934	1.852222
H	-2.002976	-5.036172	0.593267
H	-3.0498	-0.71825	1.092958
H	-1.345757	-0.388298	0.691112
H	-0.943921	-3.162456	-1.591944
H	-0.225764	-1.624766	-1.026693
H	-4.012193	-2.822647	0.316025
H	-3.210927	-3.751779	-0.97436
H	6.133177	0.339796	2.36502
H	6.492203	1.871767	1.515914
H	7.69993	0.566928	1.559556
H	6.744913	-1.478412	-0.873515
H	7.090468	0.101249	-1.6374
H	8.039295	-0.443413	-0.235533
H	0.534807	0.22061	-2.17151
H	4.231502	1.420509	1.73095
H	4.936041	-0.678771	-2.003281
H	1.891542	1.675266	1.138391
H	2.596205	-0.420755	-2.562584
H	-2.478342	3.754588	2.112847
H	-4.947086	3.951209	1.653774
H	-5.941727	2.736573	-0.258769
H	-4.567198	1.339288	-1.76009

Z-2A

O	-3.047644	-2.338681	-2.29514
N	-0.022579	2.036199	0.426757
N	1.568085	3.137464	-1.250837
N	4.940875	-1.246572	0.612085
N	-1.942398	-0.713256	0.614518
C	-0.969869	1.456437	1.444187
C	-1.060111	-0.071517	1.552701

C	2.335502	2.358051	-0.289332
C	0.819839	4.16887	-0.549346
C	0.639533	2.262784	-1.954459
C	1.396712	1.581274	0.668527
C	-0.065385	3.540669	0.556607
C	-0.416526	1.675219	-0.983545
C	5.947281	-0.873314	-0.36263
C	5.369159	-1.739467	1.906878
C	3.645013	-1.44206	0.206885
C	2.66554	-1.972907	1.08144
C	3.227662	-1.138415	-1.112397
C	1.348306	-2.134838	0.671397
C	1.906566	-1.310114	-1.501683
C	0.920341	-1.768585	-0.613759
C	-0.47069	-1.892645	-1.075339
C	-1.62643	-1.462339	-0.533002
C	-2.935517	-1.740119	-1.249581
C	-5.354778	-1.078855	-0.551618
C	-6.103183	-0.421214	0.422647
C	-5.459827	0.16951	1.515006
C	-4.070587	0.130666	1.66855
C	-3.331889	-0.529961	0.687301
C	-3.972937	-1.124375	-0.407134
H	-0.640167	1.862481	2.411077
H	-1.959226	1.879349	1.219734
H	-1.43105	-0.267423	2.572586
H	-0.069366	-0.531377	1.503738
H	2.972105	1.641696	-0.824495
H	2.990225	3.03779	0.272608
H	0.20071	4.712947	-1.275029
H	1.518487	4.890456	-0.105527
H	1.211677	1.450291	-2.420869
H	0.143921	2.826006	-2.75618
H	1.430227	0.503584	0.476312
H	1.615333	1.763746	1.72918
H	-1.118816	3.839766	0.48123
H	0.289634	3.776528	1.568691
H	-0.468459	0.584343	-1.048391
H	-1.424514	2.083564	-1.139069
H	5.703645	0.089488	-0.84107
H	6.069286	-1.631844	-1.15651
H	6.910991	-0.752132	0.144397
H	5.265083	-2.835617	1.996858
H	4.795656	-1.270368	2.722727
H	6.42383	-1.483544	2.057642
H	-0.632205	-2.381753	-2.043244
H	2.938898	-2.298267	2.083646
H	3.946572	-0.792339	-1.853495
H	0.636633	-2.59219	1.362922
H	1.630186	-1.085539	-2.535475
H	-5.824231	-1.556485	-1.412617
H	-7.188884	-0.36995	0.341372
H	-6.056994	0.673706	2.276849
H	-3.608266	0.58916	2.544056

Z-2B

O	-1.542517	-2.610402	2.577424
N	-1.245125	1.880555	-0.223219
N	-3.26472	3.287277	0.494658
N	5.941749	0.254283	-0.522549
N	-0.70375	-1.320503	-0.600098

C	-0.001553	1.123132	-0.628047
C	-0.153392	-0.216852	-1.35313
C	-3.387696	2.933759	-0.911939
C	-2.047266	4.057343	0.696341
C	-3.203436	2.075251	1.299443
C	-2.239617	1.98402	-1.348915
C	-0.817929	3.275866	0.169527
C	-1.935755	1.248889	0.961894
C	6.820005	0.998797	0.358191
C	6.46137	-0.25205	-1.777177
C	4.720207	-0.166921	-0.071011
C	3.894378	-1.011988	-0.852419
C	4.235539	0.206914	1.207415
C	2.650464	-1.417072	-0.394438
C	2.997675	-0.223044	1.654249
C	2.153774	-1.016392	0.857724
C	0.858591	-1.458466	1.369399
C	-0.31515	-1.631195	0.727002
C	-1.496939	-2.25183	1.419954
C	-3.857777	-2.782129	0.452069
C	-4.660516	-2.675392	-0.67976
C	-4.143328	-2.103214	-1.849144
C	-2.833746	-1.623694	-1.9237
C	-2.035972	-1.729449	-0.781541
C	-2.551423	-2.303998	0.388975
H	0.550777	1.796743	-1.298432
H	0.595632	0.997572	0.284928
H	0.872901	-0.449797	-1.674173
H	-0.735568	-0.101414	-2.275086
H	-4.355792	2.443576	-1.078955
H	-3.376328	3.85514	-1.509549
H	-1.938422	4.2724	1.767595
H	-2.132097	5.019518	0.173945
H	-4.10482	1.477746	1.108824
H	-3.206419	2.345451	2.363533
H	-2.602871	0.967759	-1.545311
H	-1.695158	2.345627	-2.232033
H	-0.02343	3.165307	0.918874
H	-0.382624	3.731614	-0.729726
H	-2.189859	0.22356	0.683847
H	-1.200554	1.220059	1.776222
H	7.09688	0.422299	1.258633
H	6.35646	1.944787	0.682637
H	7.740302	1.25004	-0.180592
H	5.793785	-0.002177	-2.61796
H	6.602827	-1.34746	-1.761406
H	7.432852	0.213099	-1.977908
H	0.795895	-1.709048	2.434789
H	4.240542	-1.384261	-1.814952
H	4.844454	0.819365	1.869923
H	2.068074	-2.114646	-1.001564
H	2.672028	0.064633	2.657271
H	-4.224172	-3.227384	1.378287
H	-5.685291	-3.046719	-0.66638
H	-4.775049	-2.042021	-2.737231
H	-2.454936	-1.221151	-2.864761

Z-2C

O	-3.699573	-2.767034	1.608596
N	0.508054	2.021044	0.334825
N	2.611056	3.479953	0.335214

N	4.555676	-1.746247	-0.590008
N	-2.174251	-0.5018	-0.616404
C	-0.726934	1.166796	0.401813
C	-1.110563	0.4319	-0.888849
C	2.002763	3.505824	1.658356
C	1.657445	3.988931	-0.641112
C	2.96581	2.105515	-0.000169
C	0.830622	2.497045	1.73172
C	0.309148	3.225166	-0.545363
C	1.696065	1.240724	-0.177583
C	5.555482	-1.86967	0.454028
C	4.978713	-1.90596	-1.968253
C	3.226412	-1.904602	-0.271873
C	2.78923	-1.964196	1.074227
C	2.232632	-2.018097	-1.273064
C	1.440453	-2.080893	1.385025
C	0.887383	-2.123067	-0.940882
C	0.448544	-2.121053	0.391746
C	-0.969136	-2.228798	0.766837
C	-2.045333	-1.564951	0.304084
C	-3.429951	-1.889617	0.819927
C	-5.689968	-0.721015	0.272184
C	-6.271914	0.321728	-0.443482
C	-5.474665	1.14832	-1.243547
C	-4.094903	0.962048	-1.357836
C	-3.523834	-0.08989	-0.644139
C	-4.31589	-0.911535	0.166356
H	-0.537927	0.424095	1.188918
H	-1.549327	1.819169	0.728953
H	-0.250618	-0.112456	-1.293993
H	-1.453792	1.129039	-1.663521
H	2.757861	3.25663	2.415593
H	1.652922	4.52598	1.86655
H	2.090942	3.881088	-1.644492
H	1.491923	5.060617	-0.468306
H	3.599926	1.69942	0.799661
H	3.558042	2.092633	-0.924632
H	1.079498	1.596251	2.308619
H	-0.087772	2.932005	2.147163
H	-0.041569	2.86934	-1.521539
H	-0.489522	3.823092	-0.08548
H	1.731336	0.298047	0.381605
H	1.499596	1.011549	-1.230598
H	5.538325	-2.859895	0.94408
H	5.419023	-1.099634	1.230333
H	6.549688	-1.720613	0.017871
H	4.485835	-1.169492	-2.622988
H	4.766551	-2.91553	-2.364611
H	6.058597	-1.731514	-2.034792
H	-1.226216	-2.94541	1.555308
H	3.509241	-1.947058	1.890701
H	2.510317	-2.05733	-2.325064
H	1.146782	-2.143421	2.435955
H	0.155565	-2.249167	-1.742697
H	-6.279135	-1.381177	0.910094
H	-7.346131	0.497468	-0.385764
H	-5.942158	1.961039	-1.802308
H	-3.510616	1.616938	-2.005682

E-3A

O	-1.142226	1.549787	1.912176

N	-1.852025	-2.262532	-0.323453
N	-0.375712	-3.946809	0.919858
N	5.138534	0.467321	0.347707
N	-2.623374	1.652754	-1.314331
C	4.631735	1.04796	2.699192
C	3.218969	0.530103	2.45318
C	5.629102	-0.046654	-2.019383
C	4.538337	0.94627	-2.405246
C	0.286559	-2.648201	0.885385
C	-0.607206	-4.408328	-0.440805
C	-1.650894	-3.816749	1.610584
C	-0.635114	-1.575999	0.257429
C	-1.401915	-3.35296	-1.256297
C	-2.610618	-2.891823	0.81885
C	-2.768651	-1.283472	-1.016959
C	-2.207568	-0.633168	-2.28201
C	-2.742453	0.802599	-2.489835
C	5.593689	0.381468	1.730154
C	6.186158	0.316357	-0.653204
C	3.840188	0.737105	0.015575
C	2.829733	0.780194	1.017189
C	3.473038	0.978855	-1.337993
C	1.524522	1.082289	0.675531
C	2.151542	1.239575	-1.642504
C	1.136521	1.310776	-0.662405
C	-0.209707	1.554141	-1.126686
C	-1.431114	1.607824	-0.523014
C	-1.83667	1.679456	0.909272
C	-4.196165	2.054975	1.925562
C	-5.547886	2.146266	1.62044
C	-5.970995	2.060593	0.283759
C	-5.07466	1.89051	-0.768692
C	-3.713737	1.812916	-0.455942
C	-3.289729	1.884953	0.878067
H	4.654123	2.139935	2.553245
H	4.959834	0.845424	3.728265
H	2.495729	1.010896	3.126924
H	3.189943	-0.553343	2.671087
H	6.44691	-0.046494	-2.753424
H	5.211877	-1.06684	-1.989365
H	4.094358	0.689088	-3.378131
H	4.983939	1.950253	-2.517188
H	0.560959	-2.349537	1.9056
H	1.21728	-2.74006	0.309688
H	-1.157212	-5.35832	-0.403075
H	0.357809	-4.605637	-0.925993
H	-1.467809	-3.410625	2.61415
H	-2.102756	-4.81007	1.733293
H	-1.000559	-0.845243	0.990644
H	-0.137767	-1.022649	-0.543852
H	-2.303135	-3.766183	-1.729443
H	-0.79082	-2.876006	-2.031932
H	-3.010695	-2.068812	1.425386
H	-3.453771	-3.436767	0.373459
H	-3.694727	-1.835771	-1.229956
H	-3.006856	-0.533255	-0.253805
H	-1.111698	-0.587175	-2.233494
H	-2.456752	-1.230489	-3.171677
H	-3.802539	0.773681	-2.773373
H	-2.212227	1.245142	-3.34662
H	5.73988	-0.680505	2.006059

H	6.582756	0.86375	1.789076
H	6.775647	1.250808	-0.718919
H	6.874127	-0.470154	-0.303472
H	-0.258401	1.655496	-2.217659
H	0.772349	1.155521	1.461012
H	1.891719	1.414543	-2.691257

E-3B

O	1.534739	1.960241	-1.665127
N	0.644171	-2.165311	0.397484
N	-0.275729	-3.116825	-1.798211
N	-4.722831	0.650231	-0.260387
N	3.009133	1.12485	1.461157
C	-4.300281	1.691898	-2.464768
C	-2.853837	1.227837	-2.335706
C	-5.141883	-0.271715	1.993076
C	-4.099293	0.70307	2.529825
C	0.511072	-4.131712	-1.111662
C	0.571964	-1.965033	-2.078607
C	-1.381776	-2.698438	-0.946696
C	1.195335	-3.54236	0.151562
C	1.010938	-1.275795	-0.765409
C	-0.866122	-2.249007	0.441421
C	1.111154	-1.61337	1.723543
C	2.617838	-1.340721	1.887538
C	2.946096	0.061424	2.456386
C	-5.199912	0.795142	-1.630836
C	-5.742767	0.280508	0.711236
C	-3.43529	0.940397	0.103617
C	-2.449356	1.222355	-0.881637
C	-3.055815	0.969792	1.474184
C	-1.151005	1.522865	-0.503902
C	-1.739992	1.240776	1.808128
C	-0.74274	1.510422	0.846031
C	0.625907	1.572026	1.32793
C	1.837202	1.476301	0.71798
C	2.244768	1.694726	-0.704726
C	4.608359	1.584874	-1.766509
C	5.936324	1.267304	-1.515633
C	6.327913	0.861131	-0.229346
C	5.424623	0.770848	0.827008
C	4.092728	1.112527	0.573004
C	3.696379	1.494147	-0.713704
H	-4.384582	2.7328	-2.113427
H	-4.63664	1.668043	-3.510495
H	-2.1746	1.872439	-2.910905
H	-2.763781	0.211294	-2.762518
H	-5.944418	-0.440568	2.724386
H	-4.670823	-1.248556	1.79006
H	-3.626939	0.316241	3.444756
H	-4.595737	1.648945	2.808258
H	-0.150274	-4.964292	-0.835795
H	1.270906	-4.5286	-1.797816
H	0.024279	-1.244052	-2.699911
H	1.443647	-2.302289	-2.655392
H	-2.09267	-3.528252	-0.835456
H	-1.909088	-1.867992	-1.435946
H	1.003126	-4.136478	1.054986
H	2.281198	-3.434104	0.03359
H	0.481413	-0.332607	-0.604644
H	2.089796	-1.090759	-0.729325

H	-1.112938	-2.961847	1.240066
H	-1.237073	-1.254546	0.725203
H	0.525224	-0.701332	1.862499
H	0.763539	-2.341701	2.469245
H	3.038982	-2.098999	2.563555
H	3.167807	-1.454938	0.940617
H	3.923903	0.018157	2.95193
H	2.234165	0.334939	3.248468
H	-5.286853	-0.203139	-2.101588
H	-6.217461	1.215776	-1.590571
H	-6.378552	1.157508	0.938476
H	-6.396417	-0.473471	0.243789
H	0.678326	1.505655	2.420976
H	-0.417105	1.76962	-1.271666
H	-1.470102	1.238025	2.869125
H	4.265102	1.896628	-2.754096
H	6.679684	1.333695	-2.310321
H	7.375463	0.615023	-0.046124
H	5.767202	0.467868	1.816769
H	-3.830414	2.112755	2.951968
H	-6.284034	2.291474	2.411362
H	-7.035926	2.140744	0.057693
H	-5.43537	1.854015	-1.79731

E-3C

O	0.304359	-2.163292	-1.562851
N	2.590971	2.086567	0.34233
N	3.399879	2.973447	-1.919015
N	-5.709497	0.179279	-0.38729
N	1.958853	-1.48537	1.489999
C	-6.317685	0.413623	1.998848
C	-4.900636	0.91989	2.245806
C	-5.080354	-0.101387	-2.760741
C	-4.014965	-1.090267	-2.301558
C	3.278511	1.522932	-1.982068
C	4.35478	3.336666	-0.883144
C	2.101855	3.554334	-1.610295
C	2.840682	0.945171	-0.612766
C	3.857105	2.880287	0.515138
C	1.546406	2.981977	-0.279815
C	2.059829	1.598712	1.66942
C	2.943452	0.603062	2.437563
C	2.261012	-0.752295	2.694706
C	-6.70571	0.674756	0.553041
C	-6.20301	-0.052606	-1.738147
C	-4.418629	-0.08591	-0.020853
C	-3.951273	0.259239	1.277858
C	-3.5192	-0.711839	-0.927886
C	-2.650477	-0.042832	1.627611
C	-2.215593	-0.971098	-0.54853
C	-1.737282	-0.65602	0.74063
C	-0.406077	-0.901281	1.248246
C	0.74307	-1.427471	0.735513
C	1.041965	-2.045555	-0.59472
C	3.269673	-3.039386	-1.485404
C	4.610181	-3.251093	-1.179877
C	5.109632	-2.868348	0.074515
C	4.304283	-2.272257	1.044531
C	2.954178	-2.070895	0.729924
C	2.453628	-2.445966	-0.524872
H	-6.365046	-0.667691	2.206043

H	-7.041264	0.909649	2.660876
H	-4.585668	0.721944	3.280828
H	-4.879087	2.016065	2.10935
H	-5.496023	-0.385613	-3.737684
H	-4.636094	0.90189	-2.869355
H	-3.173831	-1.127833	-3.008276
H	-4.44847	-2.105546	-2.273861
H	2.546443	1.259716	-2.756684
H	4.242898	1.092547	-2.281445
H	4.503085	4.42468	-0.89211
H	5.321288	2.870235	-1.115344
H	1.405568	3.338569	-2.431164
H	2.207422	4.64546	-1.544449
H	1.902348	0.381164	-0.669559
H	3.605469	0.299317	-0.167936
H	3.610915	3.722624	1.175977
H	4.58063	2.237911	1.030738
H	0.652038	2.360453	-0.421765
H	1.313049	3.762662	0.456015
H	1.885353	2.508865	2.258757
H	1.082106	1.1597	1.435463
H	3.888888	0.414337	1.906986
H	3.229722	1.044569	3.403613
H	1.343857	-0.615749	3.282481
H	2.922365	-1.365325	3.325033
H	-6.863826	1.758387	0.39041
H	-7.661873	0.176734	0.324489
H	-6.78331	-0.994784	-1.765215
H	-6.904875	0.760948	-1.983629
H	-0.315492	-0.568243	2.288733
H	-2.322845	0.207545	2.641933
H	-1.543198	-1.451118	-1.257712
H	2.846368	-3.323078	-2.450318
H	5.274689	-3.719343	-1.906203
H	6.161827	-3.046947	0.304586
H	4.722147	-2.000681	2.015005

E-3D

O	1.563246	2.21194	-1.360266
N	1.064284	-2.820302	-0.299873
N	-1.349032	-2.791721	-1.176297
N	-4.785305	1.113324	-0.103568
N	3.061263	0.594289	1.445398
C	-4.210918	2.420045	-2.123891
C	-2.873874	1.687581	-2.140281
C	-5.330315	-0.150941	1.948959
C	-4.125443	0.510412	2.609212
C	-1.330972	-2.376496	0.218556
C	-0.81405	-4.138705	-1.284603
C	-0.529184	-1.878783	-1.961451
C	0.098078	-2.501655	0.81347
C	0.680906	-4.160786	-0.883157
C	0.903334	-1.771511	-1.373909
C	2.496696	-2.94032	0.19756
C	3.320427	-1.672475	0.399637
C	3.143679	-0.842948	1.70064
C	-5.247719	1.570658	-1.408327
C	-5.842016	0.733459	0.824321
C	-3.462577	1.088751	0.240745
C	-2.455765	1.361387	-0.727858
C	-3.065252	0.788829	1.573941

C	-1.120459	1.353843	-0.363834
C	-1.718764	0.752507	1.88667
C	-0.706929	1.03125	0.944199
C	0.66527	0.870608	1.37415
C	1.871139	1.032368	0.767362
C	2.27141	1.78082	-0.462828
C	4.649486	2.542394	-1.188015
C	6.002132	2.42492	-0.89488
C	6.42056	1.657043	0.203979
C	5.513036	1.004026	1.035218
C	4.154888	1.146152	0.747364
C	3.735715	1.889821	-0.359307
H	-4.096112	3.384211	-1.603057
H	-4.562118	2.634738	-3.142701
H	-2.096177	2.289005	-2.631757
H	-2.980991	0.757266	-2.728203
H	-6.140244	-0.318863	2.672379
H	-5.040391	-1.134487	1.542749
H	-3.716829	-0.119475	3.413061
H	-4.442883	1.456708	3.081004
H	-1.677444	-1.338096	0.283756
H	-2.028924	-3.000496	0.792903
H	-0.927334	-4.498499	-2.316067
H	-1.398618	-4.805462	-0.636526
H	-0.994379	-0.884786	-1.957759
H	-0.497149	-2.236817	-2.99937
H	0.43059	-1.564694	1.269621
H	0.188851	-3.31539	1.545916
H	1.343341	-4.322982	-1.743736
H	0.910874	-4.918838	-0.122668
H	1.073316	-0.798713	-0.897778
H	1.689194	-1.946388	-2.120931
H	2.43975	-3.540608	1.117769
H	2.996449	-3.554619	-0.563729
H	4.363776	-2.018118	0.3468
H	3.21493	-1.013619	-0.474493
H	3.990683	-1.025126	2.376148
H	2.253059	-1.148194	2.265931
H	-5.522302	0.697429	-2.030643
H	-6.170075	2.153053	-1.252725
H	-6.315967	1.642338	1.241461
H	-6.619285	0.203676	0.250391
H	0.748051	0.486489	2.399407
H	-0.364026	1.609357	-1.107342
H	-1.435896	0.502069	2.91402
H	4.291892	3.121357	-2.040736
H	6.745081	2.928108	-1.514286
H	7.487436	1.574882	0.419597
H	5.86033	0.425405	1.892233

E-3E

O	-0.978726	3.334482	0.942272
N	3.800181	-2.173071	-0.164769
N	4.597637	-3.923629	1.525121
N	-5.735338	-1.145894	0.276548
N	1.468633	2.11727	-1.310467
C	-5.661308	-2.475188	-1.806852
C	-4.146956	-2.540441	-1.637914
C	-5.791314	0.230611	2.327347
C	-4.929909	1.253968	1.59599
C	5.280866	-2.660992	1.768917

C	4.898042	-4.382233	0.176606
C	3.159907	-3.73304	1.659358
C	4.935342	-1.62722	0.664578
C	4.287493	-3.416437	-0.869919
C	2.675349	-2.563992	0.762331
C	3.346565	-1.174462	-1.203871
C	2.666055	0.068995	-0.665465
C	2.23206	1.00567	-1.809364
C	-6.315457	-2.264246	-0.451142
C	-6.583202	-0.583406	1.317589
C	-4.498982	-0.632982	-0.022529
C	-3.657911	-1.281842	-0.965642
C	-4.035888	0.556409	0.600794
C	-2.426666	-0.728609	-1.26397
C	-2.785271	1.062877	0.294693
C	-1.942537	0.445245	-0.650204
C	-0.629841	0.90393	-1.059765
C	0.177284	1.943433	-0.713936
C	-0.00931	3.075312	0.248927
C	1.643668	4.969583	0.898492
C	2.927298	5.465245	0.693849
C	3.79121	4.817832	-0.200855
C	3.405484	3.681641	-0.911872
C	2.108718	3.20028	-0.709335
C	1.251645	3.83239	0.197684
H	-5.921284	-1.640338	-2.477615
H	-6.055142	-3.397402	-2.257228
H	-3.647782	-2.670082	-2.609811
H	-3.890325	-3.423987	-1.025584
H	-6.48909	0.718377	3.022676
H	-5.148304	-0.440707	2.920215
H	-4.322618	1.839892	2.30029
H	-5.58437	1.971728	1.070427
H	4.982193	-2.284251	2.756284
H	6.36545	-2.829776	1.796098
H	4.494184	-5.393128	0.033263
H	5.988157	-4.44386	0.058618
H	2.914046	-3.521471	2.708256
H	2.653816	-4.668527	1.385833
H	4.613329	-0.663886	1.075604
H	5.768427	-1.449067	-0.028866
H	3.414821	-3.842697	-1.382672
H	5.010622	-3.093844	-1.630373
H	2.420331	-1.672701	1.34691
H	1.811527	-2.825462	0.136404
H	4.245871	-0.918682	-1.784147
H	2.666649	-1.727785	-1.869018
H	1.760195	-0.194113	-0.099604
H	3.328969	0.638307	0.004142
H	1.641808	0.454519	-2.557424
H	3.108342	1.394079	-2.347506
H	-6.23709	-3.187542	0.155839
H	-7.391358	-2.062539	-0.581861
H	-7.371571	0.044594	0.858956
H	-7.096041	-1.418336	1.823074
H	-0.204552	0.247817	-1.828627
H	-1.808933	-1.232039	-2.015567
H	-2.448703	1.975666	0.784091
H	0.946148	5.441525	1.592052
H	3.266169	6.355446	1.22421
H	4.795872	5.21695	-0.353482

H	4.097631	3.214738	-1.614194

Z-3A

O	4.487265	1.925973	2.272093
N	-1.288728	-2.162057	-0.398826
N	-3.046357	-3.712144	0.626523
N	-3.667899	1.71457	-0.400014
N	3.03334	0.383013	-0.528595
C	-4.246026	0.653299	1.744846
C	-2.988611	1.236804	2.377699
C	-3.143695	3.263688	-2.280274
C	-1.762499	2.62198	-2.327054
C	-2.008441	-4.508085	-0.012788
C	-3.67941	-2.852087	-0.363729
C	-2.451214	-2.890216	1.671341
C	-0.976735	-3.596159	-0.729988
C	-2.661664	-1.834776	-0.937085
C	-1.313851	-2.0055	1.099849
C	-0.300954	-1.221234	-1.039586
C	1.094757	-1.181322	-0.438373
C	1.948892	-0.201832	-1.270116
C	-4.70937	1.54053	0.60251
C	-4.138262	2.294486	-1.661915
C	-2.339536	1.845866	-0.001239
C	-1.947086	1.553093	1.328264
C	-1.345733	2.226892	-0.933019
C	-0.595304	1.569375	1.661589
C	-0.007489	2.261004	-0.545237
C	0.40933	1.879644	0.735153
C	1.820221	1.821505	1.153041
C	2.882083	1.204054	0.598267
C	4.237134	1.28411	1.276566
C	6.456888	0.091687	0.63072
C	7.035483	-0.792863	-0.275407
C	6.262334	-1.332114	-1.310079
C	4.913212	-1.010722	-1.475487
C	4.345171	-0.117557	-0.568249
C	5.113564	0.416748	0.474782
H	-4.041907	-0.35864	1.353452
H	-5.051663	0.554464	2.485781
H	-2.572751	0.55362	3.135032
H	-3.252555	2.161811	2.917973
H	-3.490653	3.542873	-3.284803
H	-3.096316	4.185289	-1.67837
H	-1.020238	3.306356	-2.761959
H	-1.796101	1.733097	-2.984854
H	-1.513485	-5.121202	0.751996
H	-2.470293	-5.193636	-0.735574
H	-4.518389	-2.319972	0.104963
H	-4.094376	-3.480188	-1.163336
H	-2.058727	-3.542281	2.462781
H	-3.236159	-2.266924	2.119847
H	0.054249	-3.789459	-0.410584
H	-1.023386	-3.682278	-1.823803
H	-2.878494	-0.796056	-0.647637
H	-2.587996	-1.877042	-2.031936
H	-0.327922	-2.312707	1.467391
H	-1.442815	-0.933816	1.300254
H	-0.269092	-1.504928	-2.102691
H	-0.75223	-0.221827	-0.965559
H	1.04708	-0.798546	0.590293

H	1.578533	-2.169889	-0.412589
H	1.305353	0.601091	-1.655038
H	2.364451	-0.710158	-2.149816
H	-5.035927	2.521751	1.001797
H	-5.583167	1.091995	0.105531
H	-4.364096	1.481513	-2.377153
H	-5.0884	2.816584	-1.468022
H	2.077198	2.312087	2.098879
H	-0.30948	1.322005	2.688271
H	0.734822	2.614283	-1.265366
H	7.025538	0.528153	1.452968
H	8.086253	-1.067837	-0.183885
H	6.725161	-2.024939	-2.01528
H	4.348223	-1.447893	-2.299341

Z-3B

O	-3.0488	1.644577	-2.998837
N	-0.486797	-1.967793	0.810456
N	-1.280001	-4.083089	-0.398035
N	4.746164	0.495197	0.246991
N	-2.04097	1.498216	0.364555
C	4.941588	2.819543	1.068299
C	3.432104	2.80732	1.284149
C	4.57969	-1.752731	-0.759612
C	3.674283	-1.152867	-1.830635
C	0.140328	-3.88467	-0.65007
C	-2.020932	-2.929252	-0.89141
C	-1.501614	-4.223936	1.033629
C	0.67677	-2.684851	0.1674
C	-1.484138	-1.618853	-0.263867
C	-1.136333	-2.913781	1.783886
C	0.056221	-0.73468	1.493821
C	-0.960208	0.162968	2.210672
C	-1.211999	1.527332	1.546656
C	5.478682	1.398713	1.124694
C	5.489855	-0.671061	-0.200523
C	3.494212	0.796687	-0.237685
C	2.77723	1.915511	0.258661
C	2.900109	0.007437	-1.255824
C	1.506073	2.193972	-0.231593
C	1.62005	0.315276	-1.702773
C	0.872816	1.381308	-1.180183
C	-0.507543	1.565105	-1.654528
C	-1.679942	1.597815	-0.990175
C	-2.969488	1.591108	-1.791219
C	-5.4211	1.378699	-0.940172
C	-6.205289	1.251027	0.204116
C	-5.59685	1.206104	1.46368
C	-4.210436	1.280145	1.623284
C	-3.433704	1.409384	0.470236
C	-4.040303	1.452554	-0.792734
H	5.168786	3.263486	0.08576
H	5.44778	3.427712	1.830646
H	3.015509	3.822964	1.220792
H	3.214455	2.437535	2.302616
H	5.193596	-2.568672	-1.165819
H	3.967445	-2.174908	0.056478
H	2.986554	-1.908626	-2.242374
H	4.2921	-0.807711	-2.67747
H	0.689245	-4.798238	-0.385156
H	0.285224	-3.712999	-1.725251

H	-3.083876	-3.064445	-0.650942
H	-1.936286	-2.882736	-1.98507
H	-0.894431	-5.060761	1.404417
H	-2.554789	-4.476552	1.2139
H	1.340019	-2.992548	0.987098
H	1.204403	-1.941649	-0.442995
H	-2.276044	-1.023455	0.204233
H	-0.955989	-0.978191	-0.980608
H	-0.424123	-3.073963	2.604286
H	-2.021641	-2.408156	2.186651
H	0.805441	-1.116354	2.200136
H	0.592489	-0.177359	0.713198
H	-1.920974	-0.34893	2.37207
H	-0.568284	0.362061	3.219322
H	-0.254761	1.994906	1.300015
H	-1.699517	2.180592	2.287624
H	5.445945	1.020737	2.16496
H	6.536872	1.387827	0.817174
H	6.234597	-0.377458	-0.965816
H	6.057076	-1.064115	0.658829
H	-0.660469	1.577232	-2.740582
H	1.000755	3.096118	0.122598
H	1.181877	-0.304131	-2.49273
H	-5.86117	1.425758	-1.937269
H	-7.290818	1.192223	0.124631
H	-6.221918	1.114637	2.353916
H	-3.774244	1.255903	2.622591

Z-3C

O	-4.566401	-2.393725	-1.79342
N	1.289554	2.302944	0.156744
N	3.14195	3.488926	-1.154172
N	3.647992	-1.606069	0.493568
N	-3.017833	-0.276488	0.541315
C	3.06119	-1.185256	2.851607
C	1.807633	-2.037623	2.68598
C	4.179242	-2.528537	-1.762124
C	2.903722	-1.833632	-2.225743
C	3.50487	3.423146	0.25522
C	1.891797	4.223979	-1.292459
C	2.960649	2.135222	-1.666019
C	2.322372	2.877104	1.093758
C	0.718267	3.427267	-0.666139
C	1.996601	1.334675	-0.759211
C	0.230393	1.604442	0.971315
C	-1.020688	1.174744	0.221447
C	-1.926891	0.395334	1.190426
C	4.123734	-1.646684	1.868706
C	4.698582	-1.864374	-0.496368
C	2.308672	-1.878226	0.211733
C	1.357274	-2.023698	1.244391
C	1.871033	-1.921724	-1.132557
C	0.005485	-2.132159	0.918898
C	0.516174	-2.041747	-1.413465
C	-0.454334	-2.092846	-0.401461
C	-1.877204	-2.108328	-0.774827
C	-2.908151	-1.345276	-0.36134
C	-4.279301	-1.544782	-0.979359
C	-6.458062	-0.177219	-0.596109
C	-6.999519	0.907413	0.088808
C	-6.193843	1.651836	0.957839

C	-4.848289	1.344071	1.172053
C	-4.317528	0.250527	0.488667
C	-5.119224	-0.49111	-0.38964
H	2.822053	-0.121742	2.664453
H	3.455946	-1.250367	3.875154
H	0.998217	-1.683374	3.342081
H	2.029181	-3.071837	3.000125
H	4.961806	-2.494163	-2.532698
H	3.957288	-3.589281	-1.565247
H	2.516926	-2.281807	-3.151716
H	3.134515	-0.776157	-2.461157
H	4.3862	2.776284	0.361447
H	3.785144	4.423488	0.611202
H	1.688765	4.408344	-2.355699
H	2.002793	5.2022	-0.80526
H	3.932929	1.625847	-1.710119
H	2.575809	2.198154	-2.693105
H	2.618537	2.070977	1.777745
H	1.81721	3.660152	1.675437
H	0.076593	2.966993	-1.426753
H	0.089895	4.0356	-0.00155
H	2.526382	0.623289	-0.110683
H	1.23499	0.778608	-1.317198
H	0.726317	0.721184	1.401821
H	-0.023058	2.295088	1.789839
H	-1.578039	2.034977	-0.178233
H	-0.761535	0.512069	-0.616812
H	-2.343896	1.076883	1.944472
H	-1.320336	-0.338313	1.737856
H	4.450848	-2.671734	2.135884
H	5.015913	-1.003692	1.936075
H	5.199014	-0.910182	-0.747753
H	5.466834	-2.506664	-0.033581
H	-2.173502	-2.800031	-1.571737
H	-0.71225	-2.286186	1.728224
H	0.19709	-2.070017	-2.459147
H	-7.05257	-0.778318	-1.28548
H	-8.04592	1.1802	-0.048089
H	-6.627124	2.500882	1.489806
H	-4.257333	1.948005	1.861421
